# Nilotinib modulates LPS-induced cognitive impairment and neuroinflammatory responses by regulating P38/STAT3 signaling

**DOI:** 10.1186/s12974-022-02549-0

**Published:** 2022-07-15

**Authors:** Jieun Kim, Hyun-ju Lee, Jin-Hee Park, Byung-Yoon Cha, Hyang-Sook Hoe

**Affiliations:** 1 Department of Neural Development and Disease, Korea Brain Research Institute (KBRI), 61, Cheomdan-ro, Dong-gu, Daegu, 41062 Korea; 2 Department of Brain and Cognitive Sciences, Daegu Gyeongbuk Institute of Science & Technology, Daegu, 42988 Korea; 3 PharmacoRex Co., Ltd., 20 Techno 1-ro, Yuseong-gu, Daejeon, 34016 Korea

**Keywords:** Nilotinib, LPS, SOD2, STAT3, p38, Microglia, Cognitive function

## Abstract

**Background:**

In chronic myelogenous leukemia, reciprocal translocation between chromosome 9 and chromosome 22 generates a chimeric protein, Bcr-Abl, that leads to hyperactivity of tyrosine kinase-linked signaling transduction. The therapeutic agent nilotinib inhibits Bcr-Abl/DDR1 and can cross the blood–brain barrier, but its potential impact on neuroinflammatory responses and cognitive function has not been studied in detail.

**Methods:**

The effects of nilotinib in vitro and in vivo were assessed by a combination of RT-PCR, real-time PCR, western blotting, ELISA, immunostaining, and/or subcellular fractionation. In the in vitro experiments, the effects of 200 ng/mL LPS or PBS on BV2 microglial cells, primary microglia or primary astrocytes pre- or post-treated with 5 µM nilotinib or vehicle were evaluated. The in vivo experiments involved wild-type mice administered a 7-day course of daily injections with 20 mg/kg nilotinib (i.p.) or vehicle before injection with 10 mg/kg LPS (i.p.) or PBS.

**Results:**

In BV2 microglial cells, pre- and post-treatment with nilotinib altered LPS-induced proinflammatory/anti-inflammatory cytokine mRNA levels by suppressing AKT/P38/SOD2 signaling. Nilotinib treatment also significantly downregulated LPS-stimulated proinflammatory cytokine levels in primary microglia and primary astrocytes by altering P38/STAT3 signaling. Experiments in wild-type mice showed that nilotinib administration affected LPS-mediated microglial/astroglial activation in a brain region-specific manner in vivo. In addition, nilotinib significantly reduced proinflammatory cytokine IL-1β, IL-6 and COX-2 levels and P38/STAT3 signaling in the brain in LPS-treated wild-type mice. Importantly, nilotinib treatment rescued LPS-mediated spatial working memory impairment and cortical dendritic spine number in wild-type mice.

**Conclusions:**

Our results indicate that nilotinib can modulate neuroinflammatory responses and cognitive function in LPS-stimulated wild-type mice.

**Supplementary Information:**

The online version contains supplementary material available at 10.1186/s12974-022-02549-0.

## Background

Chronic myelogenous leukemia (CML) arises from reciprocal translocation between chromosomes 22 and 9. This translocation produces a fusion gene encoding a chimera of Bcr (breakpoint cluster region protein) and Abl (ABL proto-oncogene 1, non-receptor tyrosine kinase) that activates tyrosine kinase signaling. Among therapeutic agents for CML, the Bcr-Abl tyrosine kinase inhibitor nilotinib crosses the blood–brain barrier (BBB) and works by interfering with signaling within leukemia cancer cells [1, 2]. Nilotinib inhibits Bcr-Abl tyrosine kinase activity and the proliferation of Bcr-Abl-expressing cells 10- to 30-fold more potently than the structurally related inhibitor imatinib [3–6].

Bcr is an essential substrate of discoidin domain receptor 1 (DDR1) for cancer cell invasion signaling [7]. DDR1 is a receptor tyrosine kinase activated by collagen that is widely expressed in neurons and glia and may modulate cell division in myeloid-derived glia [8–10]. Several recent studies have shown that the multi-tyrosine kinase inhibitor nilotinib binds DDR1 and affects DDR1 activity. Specifically, nilotinib inhibits DDR1 activity [IC_50_ = 1–8 nM] more potently than it inhibits c-Abl [IC_50_ = 20 nM] in vitro and in vivo [7, 11]. Furthermore, in AD and PD patients, a nilotinib concentration of 4.7 nM in the CSF suppresses only DDR1 activity and not Abl activity [1, 12–14]. Interestingly, nilotinib attenuates the collagen-induced increase in proinflammatory cytokine production in BV2 microglial cells, suggesting that nilotinib might modulate inflammatory responses by regulating DDR1 activity [10]. Moreover, a recent study found that ischemia/reperfusion injury-induced overexpression of DDR1 leads to blood–brain barrier (BBB) degeneration in the central nervous system (CNS) [15]. However, it is unclear whether nilotinib modulates neuroinflammatory responses and neuroinflammation-linked changes in learning and memory behavior and synaptic function.

Pathogens or molecules that induce inflammation (e.g., LPS and collagen) activate microglia to regulate neuroinflammatory responses in the CNS. Neuroinflammation is accompanied by oxidative stress in the CNS [16]. Superoxide dismutase 2 (SOD2), a major antioxidant enzyme in mitochondria, is reportedly induced in the CNS under various neuroinflammatory conditions [17]. SOD2 efficiently eliminates superoxide generated from molecular oxygen in the respiratory chain [17]. In BV2 microglial cells and primary microglia, LPS significantly increases SOD2 gene and protein levels [17], suggesting that targeting SOD2 may provide additional potential therapeutic benefits by reducing LPS-mediated neuroinflammation.

Based on the literature and our preliminary findings, we hypothesized that nilotinib ameliorates neuroinflammatory responses by regulating neuroinflammation-associated factors, thereby altering neuroinflammation-linked changes in learning and memory. To test our hypothesis, the impact of nilotinib on LPS-induced neuroinflammatory responses and cognitive function was evaluated in vitro and in vivo in the present study. Pre- and post-treatment of BV2 microglial cells with nilotinib suppressed the LPS-mediated increases in the proinflammatory cytokines COX-2, IL-1β, and IL-6 by modulating AKT/P38/STAT3 signaling. In addition, Sod2 knockdown specifically reduced the effects of nilotinib on LPS-stimulated IL-6 gene expression, indicating that nilotinib affects LPS-mediated IL-6 levels in a Sod2-dependent manner. In primary microglia and primary astrocytes, nilotinib treatment significantly downregulated LPS-evoked proinflammatory cytokines and p-P38/p-STAT3 levels. In LPS-treated wild-type mice, nilotinib treatment suppressed microglial activation/morphology, astrocyte morphology, and proinflammatory cytokines by inhibiting P38 and STAT3 signaling. Importantly, nilotinib rescued LPS-induced spatial memory impairment and cortical dendritic spine number in wild-type mice. Taken together, these data suggest that nilotinib may be a new therapeutic drug for neuroinflammation and neuroinflammation-related memory impairment diseases.

## Methods

### Ethics statement

All experiments were approved by the institutional biosafety committee (IBC) and performed in accordance with approved animal protocols of the Korea Brain Research Institute (KBRI, approval no. IACUC-21-00021).

### In vivo LPS and nilotinib treatments

LPS (Sigma, Cat No. L2630, *Escherichia coli*) was dissolved in PBS for in vivo experiments. To induce the CNS immune response including neuroinflammation in vivo, an LPS dose of 10 mg/kg and a time interval of 8 h were chosen because of clear-cut evidence in the literature that these conditions are associated with proinflammatory cytokine activation after acute LPS administration [18, 19]. Nilotinib (Cayman Chemical, Cat No. 10010422, Ann Arbor, MI, USA) was dissolved in dimethyl sulfoxide (DMSO) for in vitro experiments or vehicle (5% DMSO, 10% PEG and 20% Tween 80 in deionized water) for in vivo experiments.

### BV2 microglial cells

The microglial cell line BV2 was a generous gift from Dr. Kyung-Ho Suk. The cells were cultured in high-glucose Dulbecco’s modified Eagle’s medium (DMEM, Invitrogen, Carlsbad, CA, USA). The medium was supplemented with 5% fetal bovine serum (FBS, Invitrogen), 100 U/mL penicillin G, and 100 μg/mL streptomycin. Cell culture was performed at 5% CO_2_ and 37 °C in a humidified environment.

### Primary astrocyte culture

Primary glial cells were isolated from postnatal day 1 C57BL6 mice as previously described [20]. Briefly, whole brains were filtered through 70-μm nylon mesh, followed by culture in low-glucose DMEM with 10% FBS, 100 U/mL penicillin, and 100 μg/mL streptomycin under the conditions described for BV2 microglial cells above. On day 14, the mixed glial cells were agitated at 200 rpm at room temperature for 12 h to remove primary microglial cells. Trypsin–EDTA (0.25%) was subsequently used to detach the primary astrocytes, which were centrifuged 3 times at 2000 rpm for 10 min before use. The following formula was used to calculate primary astrocyte purity: [astrocyte purity (%) = (GFAP- and DAPI-positive cells/DAPI-positive cells) × 100].

### Primary microglial culture

Primary glial cells were isolated from postnatal day 1 Sprague Dawley rats as previously described [20]. Briefly, the rat cortex was isolated and filtered through a 70-μm nylon mesh, followed by culture in low-glucose DMEM with 10% FBS, 100 U/mL penicillin, and 100 μg/mL streptomycin in a 5% CO_2_ incubator. On day 14, the cells were subjected to mild trypsin digestion (0.5 M EDTA, 1 M CaCl_2_, and 0.25% Trypsin EDTA in low-glucose DMEM with 1% penicillin and streptomycin) for 50 min in the incubator. After washing (to remove the astrocyte layer), the primary microglia were detached by trypsin–EDTA (0.25%) digestion and centrifuged twice at 2000 rpm for 10 min before use. The following formula was used to calculate primary microglial purity: [primary microglial purity (%) = (CD11b- and DAPI-positive cells/DAPI-positive cells) × 100].

### MTT assay

The MTT assay was performed to assess the cytotoxicity of nilotinib in microglial cells. BV2 microglial cells were incubated in FBS-free medium in 96-well plates (1 × 10^4^ cells/well) for 1 h before treatment with 0.1, 1, 5, 10, 25 or 50 μM nilotinib or 1% DMSO (vehicle) for 24 h. Next, the cells were incubated with MTT solution for 2 h, followed by the addition of DMSO to dissolve the formazan product. Twenty minutes later, a SPECTROstar Nano microplate reader (BMG Labtech, Germany) was used to quantify the absorbance at 570 nm (reference wavelength 660 nm).

### Reverse transcription-polymerase chain reaction (RT-PCR)

Proinflammatory cytokine IL-1β levels in BV2 microglial cells were assessed by RT-PCR. cDNA was reverse-transcribed from total RNA extracted using QIAzol Lysis Reagent (Qiagen, Cat No. 79306) and used as the template in 30-cycle RT-PCR using Prime Taq Premix (GeNetBio). The primer sequences are provided in Table 1. After separation of the amplicons by electrophoresis on a 2% agarose gel stained with EcoDye (1:5000, Biofact, Daejeon, Korea), gel images were analyzed using the software Fusion Capt Advance (Vilber Lourmat, Eberhardzell, Germany).

**Table 1:** Sequences of primers used for RT-PCR

Gene name	Sequence
*il-1β*	
Sense	5′-AGC TGG AGA GTG TGG ATC CC-3′
Antisense	5′-CCT GTC TTG GCC GAG GAC TA-3′
*gapdh*	
Sense	5′-CAG GAG CGA GAC CCC ACT AA-3′
Antisense	5′-ATC ACG CCA CAG CTT TCC AG-3′

### Real-time PCR

The mRNA levels of proinflammatory cytokines, Sod1, Sod2, Nlrp3, Nrf2, and Sirt3 in BV2 microglial cells and/or primary microglia/astrocytes were evaluated by real-time PCR as previously described [20, 21]. In brief, cDNA synthesized with Superscript cDNA Premix Kit II (GeNetBio) was analyzed by 40-cycle real-time PCR using Fast SYBR Green Master Mix (Thermo Fisher Scientific, CA, USA) and a QuantStudio™ 5 system (Thermo Fisher Scientific). The GAPDH cycle threshold (Ct) value was used for normalization. The fold change in cells treated with LPS or LPS + nilotinib was calculated relative to the control (vehicle treatment). The primer sequences are provided in Table 2.

**Table 2:** Sequences of primers used for real time-PCR

Gene name	Sequence
*il-1β*	
Sense	5′-TTG ACG GAC CCC AAA AGA TG-3′
Antisense	5′-AGG ACA GCC CAG GTC AAA G-3′
*il-6*	
Sense	5′-CCA CGG CCT TCC CTA CTT C-3′
Antisense	5′-TTG GGA GTG GTA TCC TCT GTG A-3′
*cox-2*	
Sense	5′-CCA CTT CAA GGG AGT CTG GA-3′
Antisense	5′-AGT CAT CTG CTA CGG GAG GA-3′
*inos*	
Sense	5′-GGA TCT TCC CAG GCA ACC A-3′
Antisense	5′-TCC ACA ACT CGC TCC AAG ATT-3′
*pro-il-1β*	
Sense	5′-TCT TTG AAG TTG ACG GAC CC-3′
Antisense	5′-TGA GTG ATA CTG CCT GCC TG-3′
*nlrp3*	
Sense	5′-TCC ACA ATT CTG ACC CAC AA-3′
Antisense	5′-ACC TCA CAG AGG GTC ACC AC-3′
*Sod1*	
Sense	5′-CCA GTG CAG GAC CTC ATT TT-3′
Antisense	5′-CAC CTT TGC CCA AGT CAT CT-3′
*Sod2*	
Sense	5′-GGC CAA GGG AGA TGT TAC AA-3′
Antisense	5′-GAA CCT TGG ACT CCC ACA-3′
*Nrf2*	
Sense	5′-CAG CAT AGA GCA GGA CAT GGA G-3′
Antisense	5′-GAA CAG CGG TAG TAT CAG CCA G-3′
*Sirt3*	
Sense	5′-TCC GGG AGG TGG GAG AAG-3′
Antisense	5′-ATC CCC TAG CTG GAC CAC AT-3′
*gapdh*	
Sense	5′-TGG GCT ACA CTG AGG ACC ACT-3′
Antisense	5′-GGG AGT GTC TGT TGA AGT CG-3′

### Immunocytochemistry

For immunocytochemistry, cells fixed for 10 min in 4% paraformaldehyde were washed three times with PBS before incubation overnight with the appropriate primary antibody in GDB buffer as described previously [20, 21]. The cells were subsequently washed with PBS and incubated at room temperature with the appropriate Alexa Fluor 488- or 555-conjugated secondary antibody for 2 h. Details of the antibodies are provided in Table 3. Finally, the cells were mounted in DAPI (Vector Laboratories, CA, USA), and a DM8 microscope (Leica Microsystems, Wetzlar, Germany) was used to capture images. ImageJ software was used for image analysis.

**Table 3:** Antibodies used for immunocytochemistry

Primary antibodies					
Immunogen	Host species	Dilution	Manufacturer	Catalog no.	Application
CD11b	Rat	1:200	Abcam	AB8878	ICC
p-AKT^S473^	Rabbit	1:500	Cell Signaling	9271	ICC
Bcr-Abl	Mouse	1:150	Santa Cruz	Sc-56887	ICC
p-STAT3^S727^	Rabbit	1:500	Abcam	AB86430	ICC
p-P38^T180/Y182^	Rabbit	1:200	Cell Signaling	9211	ICC

Secondary antibodies					
Antibody	Dilution	Manufacturer	Catalog no.	Application	
Goat anti-rabbit IgG, 555	1:200	Invitrogen	A21428	ICC	
Goat anti-rat IgG, 488	1:200	Invitrogen	A11006	ICC	
Goat anti-mouse IgG, 555	1:200	Invitrogen	A21422	ICC	

### Nuclear fractionation

Nuclear p-STAT3 levels were assessed in BV2 microglial cells treated with 200 ng/mL LPS or PBS for 30 min followed by 5 μM nilotinib or 1% DMSO (vehicle) for 5.5 h. To lyse the cells, cytosolic fractionation buffer (10 mM HEPES pH 8.0, 1.5 mM MgCl_2_, 10 mM KCl, 0.5 mM DTT, 300 mM sucrose, 0.5 mM PMSF and 0.1% NP-40) was added, and after 5 min, the cells were centrifuged for 1 min at 10,000 rpm and 4 °C. After removal of the supernatant (cytosolic fraction), nuclear fractionation buffer (10 mM HEPES pH 8.0, 100 mM KCl, 100 mM NaCl, 0.2 mM EDTA, 0.5 mM DTT, 0.5 mM PMSF and 20% glycerol) was added and incubated for 15 min on ice. Finally, the nuclear lysate was centrifuged for 15 min at 10,000 rpm and 4 °C. The nuclear fraction was subsequently used in western blotting to detect nuclear p-STAT3 levels.

### Western blotting

Western blotting was performed on BV2 microglial cells, primary microglia, primary astrocytes, or cortical/hippocampal tissue from mice treated with LPS or PBS followed by nilotinib or DMSO (vehicle). The treated cells or tissues were lysed in ProPrep lysis buffer (iNtRON Biotechnology, Inc., Seongnam, Korea) and centrifuged for 15 min at 12,000 rpm. After quantification of the protein concentration in the supernatant relative to a standard BSA solution, 20 μg of protein was separated by electrophoresis on an 8% SDS gel. The proteins were then transferred to a polyvinylidene difluoride (PVDF) membrane, which was blocked with 5% skim milk or 5% BSA at room temperature for 1 h before incubation overnight with anti-c-Abl (1:500, Santa Cruz), anti-p-AKTser473 (1:1000, Cell Signaling), anti-AKT (1:1000, Cell Signaling), anti-p-P38 (1:1000, Cell Signaling), anti-P38 (1:1000, Cell Signaling), anti-pSTAT3^s727^ (1:1000, Cell Signaling), anti-β-actin (l:1000, Santa Cruz) or anti-PCNA (l:1000, Santa Cruz) at 4 °C. The membrane was subsequently incubated with HRP-conjugated goat anti-mouse or anti-rabbit IgG (both 1:1000, Enzo Life Sciences, Farmingdale, NY, USA) for 1 h. ECL Western Blotting Detection Reagent (GE Healthcare, Chicago, IL, USA) was used for detection. The membranes were then stripped, incubated with anti-P38 or anti-AKT (both 1:1000, Cell Signaling) and developed accordingly. For image acquisition and analysis, Fusion Capt Advance software (Vilber Lourmat) was used.

### Sod2-siRNA transfection

To determine whether nilotinib affects LPS-evoked inflammatory responses in a Sod2-dependent manner, Sod2 was knocked down in BV2 microglial cells. Opti-MEM medium (Thermo Scientific, Waltham, MA, USA) was used to dilute Sod2 siRNA or control (scramble) siRNA (Dharmacon, Lafayette, CO, USA), which was subsequently incubated for 40 min at a final concentration of 30 nm with 1 µL of Lipofectamine® RNAiMAX reagent (Thermo Scientific, Waltham, MA, USA). This mixture was then added to BV2 microglial cells in a 24-well cell culture plate (2 × 10^5^ cells/well). Twenty-four hours later, 5 μM nilotinib or 1% DMSO (vehicle) was added, followed 30 min later by 200 ng/mL LPS or PBS. Twenty-four hours after the addition of nilotinib or vehicle, real-time PCR was performed to measure Sod2 and proinflammatory cytokine mRNA levels.

### Enzyme-linked immunosorbent assay (ELISA)

The effects of nilotinib on cortical and hippocampal levels of IL-1β, IL-6, and COX-2 were assessed in wild-type mice. Injections of 20 mg/kg nilotinib (i.p.) or vehicle (5% DMSO + 10% PEG + 20% Tween 80) were administered to the mice daily for 7 days. On day 7, LPS (10 mg/kg, i.p.) or PBS was administered 30 min after the final injection of nilotinib or vehicle. After 8 h, the cortex and hippocampus were dissected and homogenized in RIPA buffer with protease and phosphatase inhibitors. The homogenate was centrifuged for 15 min at 10,000 rpm and 4 °C, and the supernatant was collected as the total protein. The BCA protein assay was used to determine the total protein concentration. ELISA kits (IL-1β: Cat. No. 88-7013-88, IL-6: Cat. No. 88-7064-88, Invitrogen, Waltham, MA, USA; COX-2: Cat. No. DYC4198-5, R&D Systems, Minneapolis, MN, USA) were used to measure the levels of IL-1β, IL-6, and COX-2 in the cortex and hippocampus as described by the manufacturer.

### Wild-type mice and immunofluorescence staining

The Korea Brain Research Institute Animal Care and Use Committee (IACUC-21-00021) approved all animal experimental protocols. Wild-type C57BL6 mice (male, 8 weeks old; Orient-Bio Company, Gyeonggi-do, Korea) were housed at 22 ± 2 °C, 50 ± 5% humidity, and a 12-h light/dark cycle in a pathogen-free facility with chow and water ad libitum. The mice were randomly allocated to 3 groups: control, LPS, and LPS + nilotinib. According to the group, the mice were administered 20 mg/kg nilotinib or vehicle (5% DMSO, 10% PEG and 20% Tween 80 in deionized water) daily by intraperitoneal (i.p.) injection for 7 days. Thirty minutes after the last injection on day 7, 10 mg/kg LPS (i.p.) or PBS was administered by i.p. injection. Eight hours after LPS or PBS injection, the mice were perfused and fixed with PBS and 4% paraformaldehyde, respectively. To obtain brain sections for immunofluorescence staining, brain tissue stored in 4% paraformaldehyde for 24 h at 4 °C was immersed in 30% sucrose in PBS for 72 h and then sliced at a thickness of 30 μm with a cryostat microtome (Leica CM1850, Wetzlar, Germany). The sections were blocked at room temperature for 2 h with 5% normal goat serum (Vector Laboratories, Burlingame, CA, USA) and subsequently immunostained at 4 °C overnight with the following primary antibodies: anti-c-Abl (1:150, Santa Cruz), anti-Iba-1 (1:500, Wako), anti-GFAP (1:500, Neuromics), anti-COX-2 (1:200, Abcam), anti-IL-1β (1:200, Abcam), anti-IL-6 (1:50, Santa Cruz), anti-p-P38 (1:200, Cell Signaling), or anti-p-STAT3^Ser727^ (1:500, Cell Signaling). Details of the antibodies are provided in Table 4. The slices were then washed with PBST buffer and incubated with Alexa 555- or Alexa 488-conjugated secondary antibodies for 2 h at room temperature. After mounting on glass slides using Vectashield mounting solution with DAPI (Vector Labs, Burlingame, CA), fluorescence microscopy images of the immunostained tissue were obtained (DMi8, Leica Microsystems, Wetzlar, Germany) and analyzed (ImageJ, US National Institutes of Health, Bethesda, MD, USA).

**Table 4:** Antibodies used for immunofluorescence staining

Primary antibodies					
Immunogen	Host species	Dilution	Manufacturer	Catalog no.	Application
Iba-1	Rabbit	1:500	Wako	019-19741	IF
GFAP	Rabbit	1:500	Neuromics	RA22101	IF
Bcr-Abl	Mouse	1:150	Santa Cruz	Sc-56887	IF
p-STAT3^S727^	Rabbit	1:500	Abcam	AB86430	IF
p-P38^T180/Y182^	Rabbit	1:200	Cell Signaling	9211	IF

Secondary antibodies					
Antibody	Dilution	Manufacturer	Catalog no	Application	
Goat anti-rabbit IgG, 555	1:200	Invitrogen	A21428	IF	
Goat anti-rabbit IgG, 488	1:200	Invitrogen	A11008	IF	
Goat anti-mouse IgG, 555	1:200	Invitrogen	A21422	IF	

### Y-maze

The ability of nilotinib to recover LPS-induced short-term and spatial memory impairments in wild-type mice was assessed by performing the Y-maze test as previously described [22]. The Y-maze was constructed of 3 arms (35 cm × 7 cm × 15 cm) that met at angles of 120°. In each test, a single mouse freely explored the maze for 5 min. A SMART video camera was used to record spontaneous alternations during the 5-min period, which were then manually counted. The alternation percentage was calculated using the following formula: [alternation percentage (%) = (number of alternations/number of alternation triads) × 100].

### Novel objective recognition test (NOR)

To evaluate the effects of nilotinib on long-term and recognition memory, the NOR test was performed as previously described [22]. The apparatus for the NOR test was an open-field box with side lengths of 40 cm and a height of 25 cm. The NOR test comprised two phases: training and testing. The training and testing phases were performed 24 h apart. In the training phase, two identical objects were placed in the box, and a single mouse explored the box for 5 min. In the testing phase, the mouse was returned to the box, which contained one familiar object and one novel object, for 5 min. The locations of the two objects in the box in the testing phase were counterbalanced among the trials. Odor cues were removed after each trial by thoroughly cleaning the box and objects with 70% ethanol. The time of exploration was manually counted by reviewing a video recording of each trial. Behavior was considered exploratory when the mouse pointed its nose toward an object. Object preference (%) for the novel object was calculated using the following formula: [object preference (%) = T Novel/(T Familiar + T Novel) × 100], where T Novel is the exploration time for the novel object and T Familiar is the exploration time for the familiar object.

### Golgi staining

After the behavioral tests, 4–5 wild-type mice were used for Golgi staining. The FD Rapid Golgi Stain kit (FD NeuroTechnologies) was used as reported previously [22]. Freshly dissected brains were first incubated in solutions A and B for 2 weeks at room temperature and then immersed in solution C for 24 h at 4 °C. The brains were sliced at a thickness of 150 μm using a vibratome (VT1000S; Leica). Bright-field microscopy (Axioplan 2; Zeiss) images (63× magnification) of pyramidal neurons in cortical layers V and CA1 were coded, and dendritic spine number was counted in a blinded manner using ImageJ (NIH). All dendritic spines with a dendrite length greater than 20 μm were counted.

### Statistical analyses

Data analysis was performed using GraphPad Prism 7 software (GraphPad Software, San Diego, CA, USA). To compare two groups, the unpaired/paired one-tailed or two-tailed *T*-test with Welch’s correction was used. For multiple comparisons, one-way ANOVA and Tukey’s test were used (Additional file 1: Tables S1, S2). Differences were considered significant at *p* < 0.05. Results are presented as the mean ± SD (*p < 0.05, **p < 0.01, ***p < 0.001).

## Results

### Nilotinib decreases LPS-stimulated proinflammatory cytokine levels and increases anti-inflammatory cytokine levels in BV2 microglial cells

**Fig. 1:**
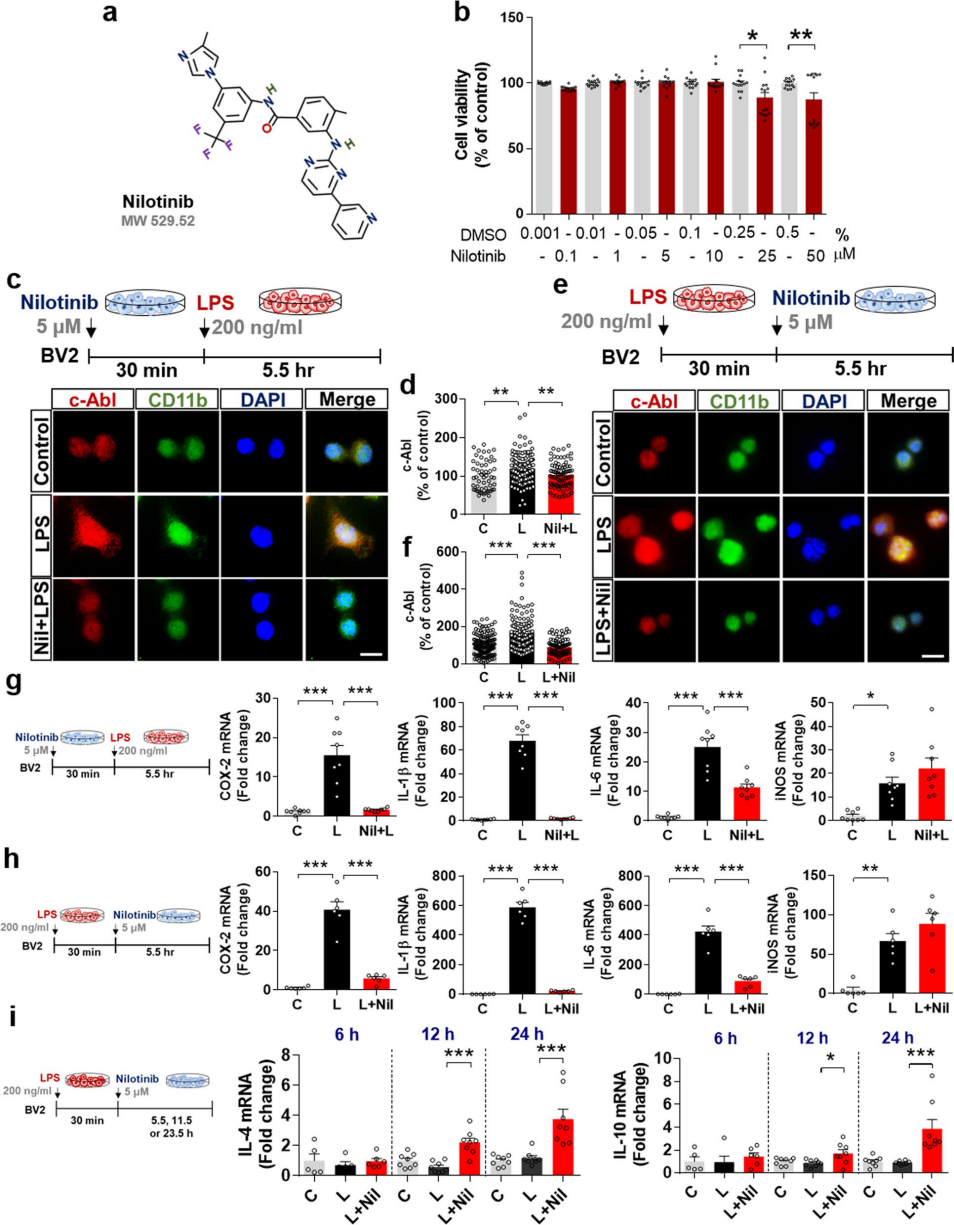
The multi-tyrosine kinase inhibitor nilotinib suppresses LPS-mediated proinflammatory responses and increases anti-inflammatory responses in BV2 microglial cells. **a** Structure of nilotinib. **b** Impact of nilotinib or vehicle on cell viability as assessed by the MTT assay (*n* = 14/group). **c**, **d** Immunocytochemistry of c-Abl expression in LPS-treated BV2 microglial cells pre-treated with nilotinib as shown (C: *n* = 54; L: *n* = 141; Nil + L: *n* = 73). **e**, **f** Immunocytochemistry of c-Abl expression in LPS-treated BV2 microglial cells post-treated with nilotinib as shown (C: *n* = 160; L: *n* = 130; L + Nil: *n* = 95). **g** Real-time PCR analysis of proinflammatory cytokine expression in LPS-treated BV2 microglial cells pre-treated with nilotinib as shown (*n* = 8/group). **h** Real-time PCR analysis of proinflammatory cytokine expression in LPS-treated BV2 microglial cells post-treated with nilotinib as shown (*n* = 6/group). **i** Real-time PCR analysis of anti-inflammatory cytokine expression in LPS-treated BV2 microglial cells post-treated with nilotinib as shown (*n* = 5–8/group). C: control, L: LPS, Nil + L: nilotinib + LPS, L + Nil: LPS + nilotinib, *p < 0.05, **p < 0.01, ***p < 0.001, scale bar = 20 μm

Nilotinib has been shown to mitigate Alzheimer’s disease (AD) and Parkinson’s disease (PD) pathologies [23, 24], but its effects on LPS-induced proinflammatory cytokine levels and the corresponding mechanism of action have not been studied. Before proceeding with in vitro experiments, we first confirmed that nilotinib had no cytotoxicity in BV2 microglial cells at concentrations up to 50 μM, as assessed by MTT assays (Fig. 1a, b).

To assess the effects of pre-treatment with nilotinib on LPS-mediated c-Abl levels, BV2 microglial cells were treated sequentially with 5 μM nilotinib or 1% DMSO (vehicle) for 30 min and 200 ng/mL LPS or PBS for 5.5 h. Immunocytochemistry with an anti-c-Abl antibody showed that nilotinib pre-treatment significantly reduced the LPS-induced increase in c-Abl levels (Fig. 1c, d). Next, the effects of nilotinib post-treatment on LPS-evoked c-Abl levels were assessed by sequentially treating BV2 microglial cells with 200 ng/mL LPS or PBS for 30 min and 5 μM nilotinib or 1% DMSO for 5.5 h. Immunocytochemistry with an anti-c-Abl antibody showed that nilotinib post-treatment also significantly downregulated the LPS-induced increase in c-Abl levels (Fig. 1e, f).

The effects of pre- or post-treatment with nilotinib on LPS-evoked proinflammatory cytokine levels in BV2 microglial cells were evaluated by real-time PCR. Pre-treatment with nilotinib significantly suppressed the LPS-induced increases in the mRNA levels of COX-2, IL-1β and IL-6 but not iNOS (Fig. 1g). Post-treatment with nilotinib also significantly downregulated LPS-mediated COX-2, IL-1β, and IL-6 but not iNOS levels in BV2 microglial cells (Fig. 1h). These data indicate that nilotinib suppresses LPS-stimulated microglial proinflammatory cytokine levels.

To explore the effects of nilotinib on LPS-mediated anti-inflammatory cytokines, BV2 microglial cells were treated with LPS (200 ng/mL) or PBS for 30 min and post-treated with nilotinib (5 μM) or vehicle (1% DMSO) for 5.5, 11.5, or 23.5 h. Real-time PCR analysis revealed that post-treatment with nilotinib for 11.5 h and 23.5 h significantly increased IL-4 and IL-10 anti-inflammatory cytokine levels, whereas post-treatment for 5.5 h had no effect (Fig. 1i). These data indicate that nilotinib increases the release of anti-inflammatory cytokines in a time-dependent manner.

### Nilotinib reduces LPS-stimulated AKT/P38/STAT3 signaling in BV2 microglial cells

**Fig. 2:**
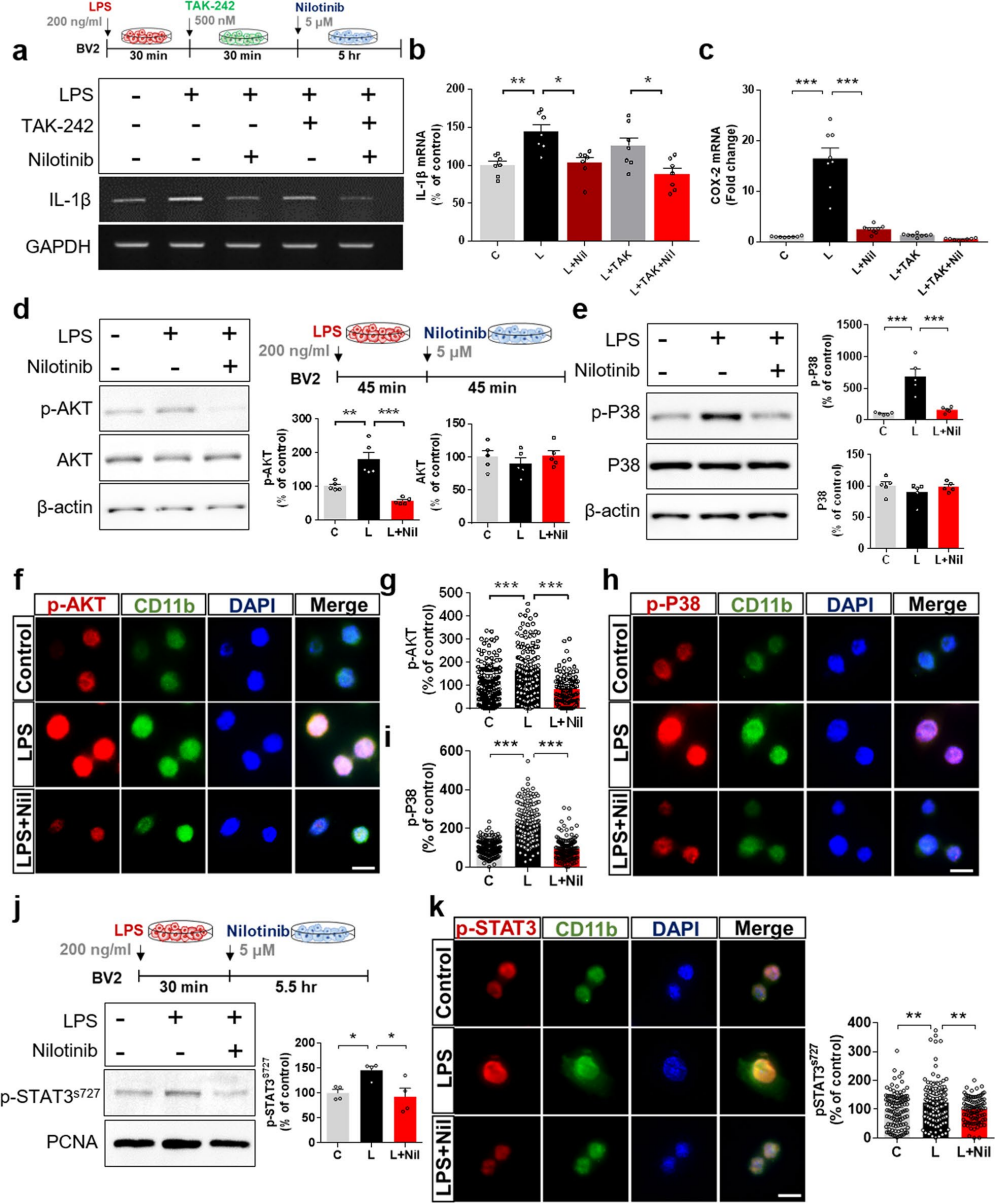
Nilotinib suppresses LPS-stimulated AKT/P38/STAT3 signaling in BV2 microglial cells. **a**, **b** RT-PCR analysis of proinflammatory cytokine IL-1β expression in BV2 microglial cells treated sequentially with LPS, TLR4 inhibitor (TAK-242) and nilotinib as shown (*n* = 7/group). **c** Real-time PCR analysis of proinflammatory cytokine COX-2 mRNA expression in BV2 microglial cells treated sequentially with LPS, TLR4 inhibitor (TAK-242) and nilotinib as shown (*n* = 8/group). **d**, **e** Western blotting analysis of AKT/P38 signaling in LPS-treated BV2 microglial cells post-treated with nilotinib as shown (*n* = 5/group). **f**–**i** Immunocytochemistry analysis of AKT/P38 signaling in LPS-treated BV2 microglial cells post-treated with nilotinib (p-AKT, C: *n* = 181; L: *n* = 162; L + Nil: *n* = 111, p-P38, C: *n* = 197; L: *n* = 246; L + Nil: *n* = 157). **j** Western blot analysis of nuclear p-STAT3 expression in LPS-treated BV2 microglial post-treated with nilotinib as shown (*n* = 4/group). **k** Immunocytochemistry analysis of p-STAT3 levels in LPS-treated BV2 microglial cells post-treated with nilotinib as shown in **j** (C: *n* = 110; L: *n* = 161; L + Nil: *n* = 94). C: control, L: LPS, Nil + L: nilotinib + LPS, *p < 0.05, **p < 0.01, ***p < 0.001, scale bar = 20 μm

Based on the literature and our findings in Fig. 1, we hypothesized that nilotinib suppresses LPS-induced proinflammatory cytokine levels by inhibiting TLR4 and/or downstream neuroinflammation-associated factors. To test our hypothesis, BV2 microglial cells were subjected to three sequential treatments: (i) 200 ng/mL LPS or PBS for 30 min; (ii) 500 nM TLR4 inhibitor (TAK-242) or 1% DMSO (vehicle) for 30 min; and (iii) 5 μM nilotinib or 1% DMSO (vehicle) for 5 h. Subsequent RT-PCR analysis showed that the LPS-induced increase in IL-1β mRNA levels was significantly reduced in BV2 microglial cells treated with LPS, TLR4 inhibitor, and nilotinib compared with cells treated with LPS and TLR4 inhibitor (Fig. 2a, b). However, no difference in LPS-mediated IL-1β mRNA levels was observed between cells treated with LPS, TLR4 inhibitor, and nilotinib and cells treated with LPS and nilotinib (Fig. 2a, b). These data indicate that the impact of nilotinib on LPS-stimulated proinflammatory cytokine IL-1β mRNA levels is independent of TLR4 signaling. In addition, real time-PCR analysis showed that the LPS-induced increase in COX-2 mRNA levels was significantly reduced in cells treated with LPS and nilotinib, LPS and TLR4 inhibitor, or LPS, TLR4 inhibitor and nilotinib compared with cells treated with LPS alone (Fig. 2c). Moreover, COX-2 mRNA levels in cells treated with LPS, TLR4 inhibitor, and nilotinib were not significantly different from those in cells treated with LPS and nilotinib or LPS and TLR4 inhibitor (Fig. 2c). These data indicate that the reduction in LPS-induced COX-2 mRNA levels by nilotinib depends on TLR4 signaling. In summary, nilotinib differentially modulates LPS-induced proinflammatory cytokine levels in a TLR signaling-independent and/or -dependent manner in BV2 microglial cells.

We then examined the effects of nilotinib on LPS-stimulated AKT/P38 signaling in BV2 microglial cells. Cells were treated for 45 min with 200 ng/mL LPS or PBS min followed by 45 min with 5 μM nilotinib or 1% DMSO (vehicle). Western blotting with anti-p-AKT, anti-AKT, anti-p-P38, and anti-P38 antibodies showed that nilotinib post-treatment significantly decreased LPS-induced p-AKT and p-P38 levels (Fig. 2d, e). These findings were confirmed by immunocytochemistry (Fig. 2f, i).

The effects of nilotinib on LPS-induced nuclear p-STAT3 and/or p-NF-kB levels were assessed in LPS-treated BV2 microglial cells post-treated with nilotinib as described above. Nuclear fractionation and western blot analysis showed that nilotinib post-treatment significantly suppressed LPS-stimulated nuclear p-STAT3 levels (Fig. 2j). Immunocytochemistry confirmed this result (Fig. 2k) and additionally showed that nilotinib post-treatment had no effect on nuclear p-NF-kB levels in BV2 microglial cells (Additional file 1: Fig. S1).

### Nilotinib modulates LPS-mediated IL-6 mRNA levels in a Sod2-dependent manner

**Fig. 3:**
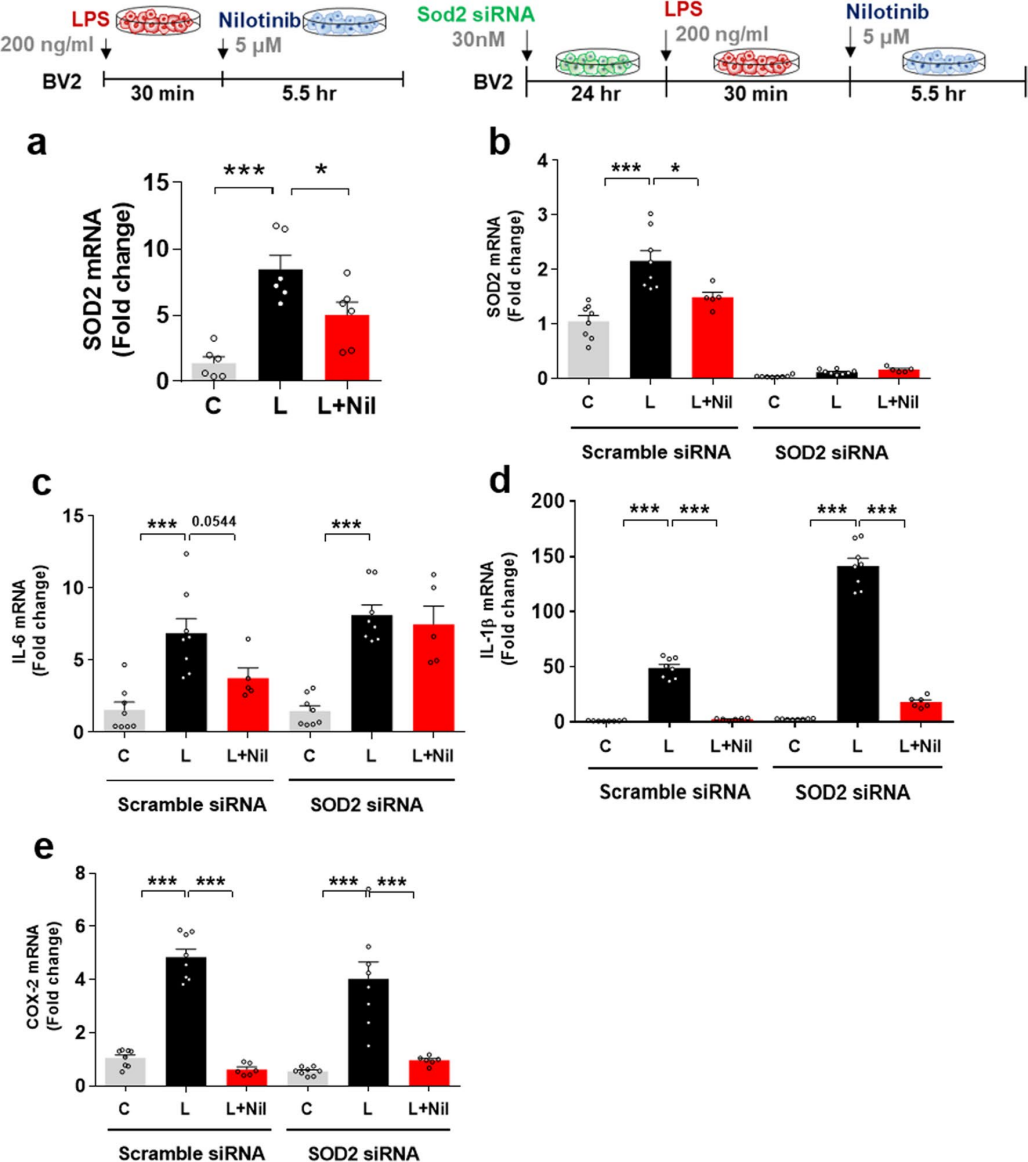
Nilotinib decreases LPS-mediated microglial neuroinflammatory responses in a Sod2-dependent manner. **a** Real-time PCR analysis of Sod2 gene expression in LPS-treated BV2 microglial cells post-treated with nilotinib as shown (*n* = 6/group). **b**–**e** Real-time PCR analysis of relative Sod2, IL-6, IL-1β, and COX-2 mRNA levels in BV2 microglial cells transfected with Sod2 siRNA (30 nM) or control (scramble) siRNA for 24 h and subsequently treated with LPS and nilotinib as shown (*n* = 8/group). C: control, L: LPS, Nil + L: nilotinib + LPS, *p < 0.05, ***p < 0.001

Since nilotinib downregulates proinflammatory cytokines via P38/AKT/STAT3 signaling pathway in BV2 microglial cells, we examined the roles of mitochondrial function and oxidative stress in these effects. Previous studies have reported that LPS stimulates oxidative stress and formation of the NLRP3 inflammasome and regulates antioxidant enzymes such as Sod1/2 and Sirt1/3 in microglia [17, 25, 26]. To verify the effects of nilotinib on proinflammatory cytokine release in response to LPS-induced activation of oxidative stress, Sod1, Sod2, Nlrp3, Nrf2, and Sirt3 mRNA levels in LPS-treated BV2 microglial cells post-treated with nilotinib as described above were first analyzed by real-time PCR. Importantly, nilotinib post-treatment significantly decreased LPS-stimulated mRNA levels of Sod2 but not Sod1, Nlrp3, Nrf2, or Sirt3 (Fig. 3a, Additional file 1: Fig. S2).

Next, we investigated whether nilotinib affects LPS-evoked proinflammatory responses through Sod2. For this experiment, BV2 microglial cells were transfected with Sod2 siRNA (30 nM) or scramble siRNA for 24 h and then treated sequentially with LPS and nilotinib as described above. Real-time PCR analysis of Sod2 and proinflammatory cytokine mRNA levels showed that transfection with Sod2 siRNA successfully reduced Sod2 mRNA levels compared with scramble siRNA (Fig. 3b). Interestingly, in cells in which Sod2 was knocked down, nilotinib post-treatment significantly reduced LPS-induced IL-1β or COX-2 mRNA levels but had no effect on LPS-induced IL-6 mRNA levels (Fig. 3c–e). These data suggest that downregulation of Sod2 is required in order for nilotinib to modulate LPS-mediated microglial IL-6 mRNA levels.

### Nilotinib alters LPS-induced pro- and anti-inflammatory cytokine levels via p38/STAT3 signaling and Sod2 mRNA levels in primary microglia

**Fig. 4:**
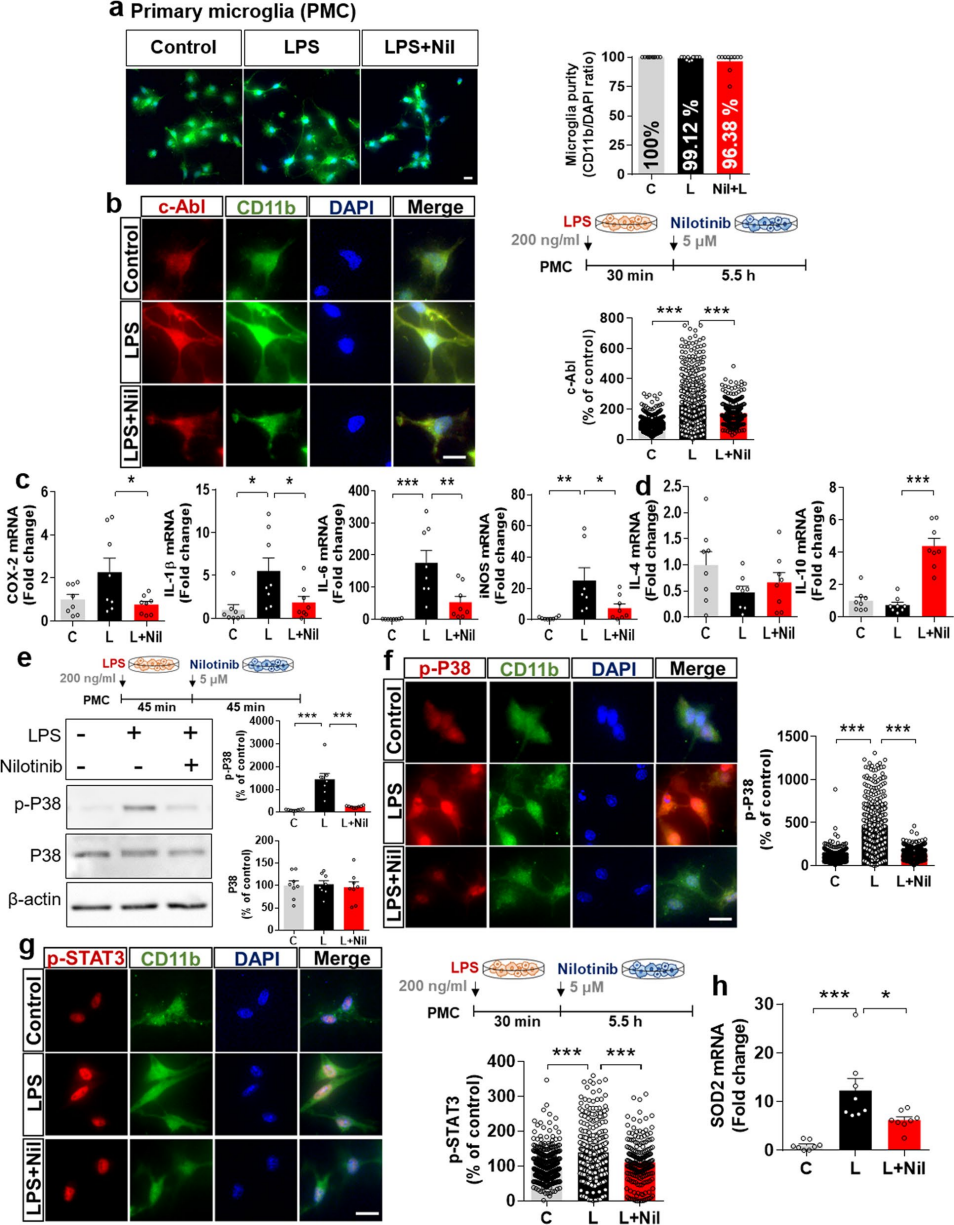
Nilotinib alters LPS-evoked changes in pro- and anti-inflammatory cytokine levels via P38/STAT signaling and suppresses *Sod2* mRNA levels in primary microglia. **a** Assessment of primary microglial purity based on the CD11b/DAPI ratio (C: *n* = 371; L: *n* = 287; L + Nil: *n* = 216). **b** Immunocytochemistry analysis of c-Abl levels in LPS-treated primary microglia post-treated with nilotinib as shown (*n* = C: *n* = 561; L: *n* = 577; Nil + L: *n* = 298). **c**, **d** Real-time PCR analysis of pro- and anti-inflammatory cytokine levels in LPS-treated primary microglia post-treated with nilotinib as shown (*n* = 8/group). **e**–**g** Western blotting analysis and immunohistochemistry staining of p-P38 and p-STAT3 levels in LPS-treated primary microglia post-treated with nilotinib as shown (p-P38 for western blot: *n* = 8/group; p-P38 for ICC: C: *n* = 526; L: *n* = 464; Nil + L: *n* = 352; p-STAT3 for ICC: C: *n* = 422; L: *n* = 358; Nil + L: *n* = 213). **h** Real-time PCR analysis of *Sod2* mRNA in LPS-treated primary microglia post-treated with nilotinib (*n* = 8/group). *p < 0.05, **p < 0.01, ***p < 0.001. Scale bar = 20 μM

Before assessing the effects of nilotinib on LPS-mediated pro- and anti-inflammatory cytokines levels in primary microglial cells, we confirmed that the primary microglial purity was approximately 96–100% by immunocytochemistry using an anti-CD11b antibody (Fig. 4a). Next, the effects of nilotinib on LPS-induced c-Abl levels were assessed in primary microglial cells treated sequentially with 200 ng/mL LPS or PBS for 30 min and 5 μM nilotinib or 1% DMSO (vehicle) for 5.5 h. Immunocytochemistry showed that nilotinib significantly decreased c-Abl protein levels in LPS-treated primary microglial cells (Fig. 4b).

To investigate whether nilotinib influences pro- and anti-inflammatory cytokine levels in primary microglia, real time-PCR was conducted. Nilotinib post-treatment significantly decreased the increases in the mRNA levels of the proinflammatory cytokines COX-2, IL-1β, IL-6 and iNOS induced by LPS in primary microglia (Fig. 4c). Similar to the effects observed in BV2 microglial cells, nilotinib post-treatment of LPS-treated primary microglial cells significantly increased levels of the anti-inflammatory cytokine IL-10 but not IL-4 (Fig. 4d).

Next, we determined whether nilotinib affects LPS-evoked P38 and STAT3 activation. Primary microglia were treated with 200 ng/mL LPS or PBS for 45 min followed by 5 μM nilotinib or 1% DMSO (vehicle) for 45 min, and western blotting was conducted with anti-p-P38, anti-P38, and anti-β-actin antibodies. Nilotinib post-treatment significantly decreased LPS-evoked p-P38 levels in primary microglia, whereas total P38 levels remained unchanged (Fig. 4e). Immunocytochemistry confirmed that nilotinib post-treatment significantly downregulated LPS-mediated microglial p-P38 levels (Fig. 4f). In addition, nilotinib post-treatment significantly suppressed LPS-stimulated nuclear p-STAT3 levels in primary microglial cells (Fig. 4g).

To identify whether nilotinib also affects *Sod2* expression to influence proinflammatory cytokine release in primary microglia, real-time-PCR was conducted. Consistent with the findings in BV2 microglial cells, nilotinib post-treatment significantly suppressed *Sod2* gene expression in LPS-treated primary microglia (Fig. 4h). Taken together, these data indicate that nilotinib downregulates proinflammatory cytokine levels via the P38/STAT3 signaling pathway and Sod2 expression in primary microglia.

### Nilotinib reduces LPS-stimulated c-Abl and proinflammatory cytokine levels in primary astrocytes

**Fig. 5:**
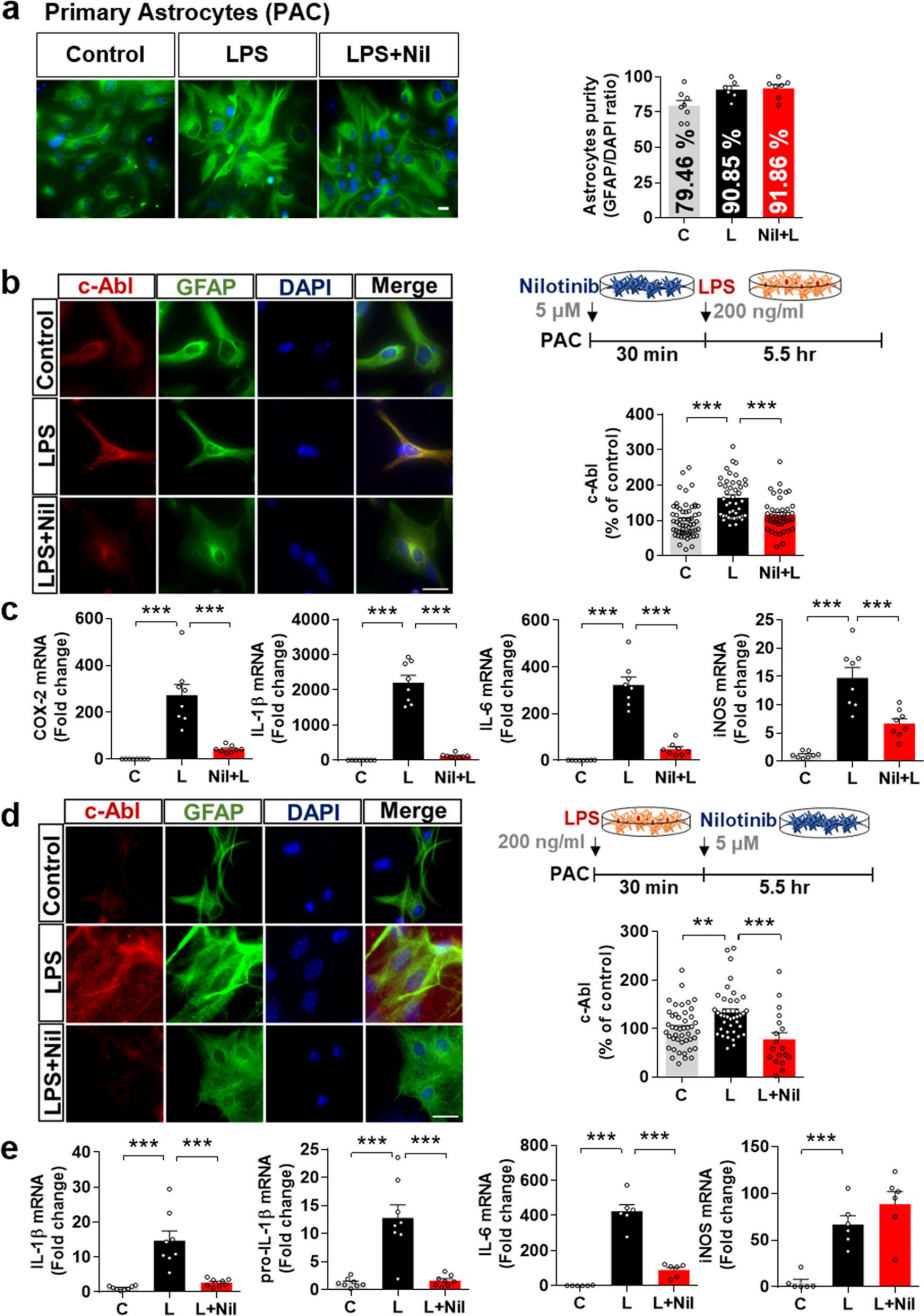
Nilotinib downregulates LPS-induced proinflammatory responses in primary astrocytes. **a** Assessment of primary astrocyte purity based on the GFAP/DAPI ratio (C: *n* = 253; L: *n* = 231; L + Nil: *n* = 236). **b** Immunocytochemistry analysis of c-Abl expression in LPS-treated primary astrocytes pre-treated with nilotinib as shown (C: *n* = 59; L: *n* = 43; Nil + L: *n* = 42). **c** Real-time PCR analysis of proinflammatory cytokine levels in LPS-treated primary astrocytes pre-treated with nilotinib as shown (*n* = 8/group). **d** Immunocytochemistry analysis of c-Abl expression in LPS-treated primary astrocytes post-treated with nilotinib as shown (C: *n* = 45; L: *n* = 39; L + Nil: *n* = 18). **e** Real-time PCR analysis of proinflammatory cytokine levels in LPS-treated primary astrocytes post-treated with nilotinib as shown (*n* = 8/group). C: control, L: LPS, Nil + L: nilotinib + LPS, L + Nil: LPS + nilotinib, **p < 0.01, ***p < 0.001, scale bar = 20 μm

Given the effects of nilotinib on LPS-induced proinflammatory cytokine levels in BV2 and primary microglial cells, we next investigated the ability of nilotinib to alter LPS-stimulated proinflammatory cytokine levels in primary astrocytes. We first confirmed that the astrocyte purity was 80–90% by immunocytochemistry using an anti-GFAP antibody (Fig. 5a).

To determine the effects of nilotinib on LPS-mediated astroglial c-Abl expression, primary astrocytes were treated with 200 ng/mL LPS or PBS for 30 min followed by 5 µM nilotinib or vehicle (1% DMSO) for 5.5 h or vice versa. Subsequent immunocytochemistry showed that both pre- and post-treatment with nilotinib significantly downregulated LPS-induced c-Abl levels in primary astrocytes (Fig. 5b, d).

We then examined whether nilotinib pre- or post-treatment differentially affected LPS-evoked astroglial proinflammatory responses. Real-time PCR demonstrated that nilotinib pre-treatment significantly decreased LPS-stimulated mRNA levels of the proinflammatory cytokines COX-2, IL-1β, IL-6, and iNOS (Fig. 5c). Post-treatment with nilotinib significantly reduced IL-1β, pro-IL-1β and IL-6 but not iNOS mRNA levels in primary astrocytes (Fig. 5e). These data suggest that nilotinib modulates LPS-stimulated proinflammatory responses in primary astrocytes.

### Nilotinib decreases LPS-stimulated P38/STAT3 phosphorylation in primary astrocytes

**Fig. 6:**
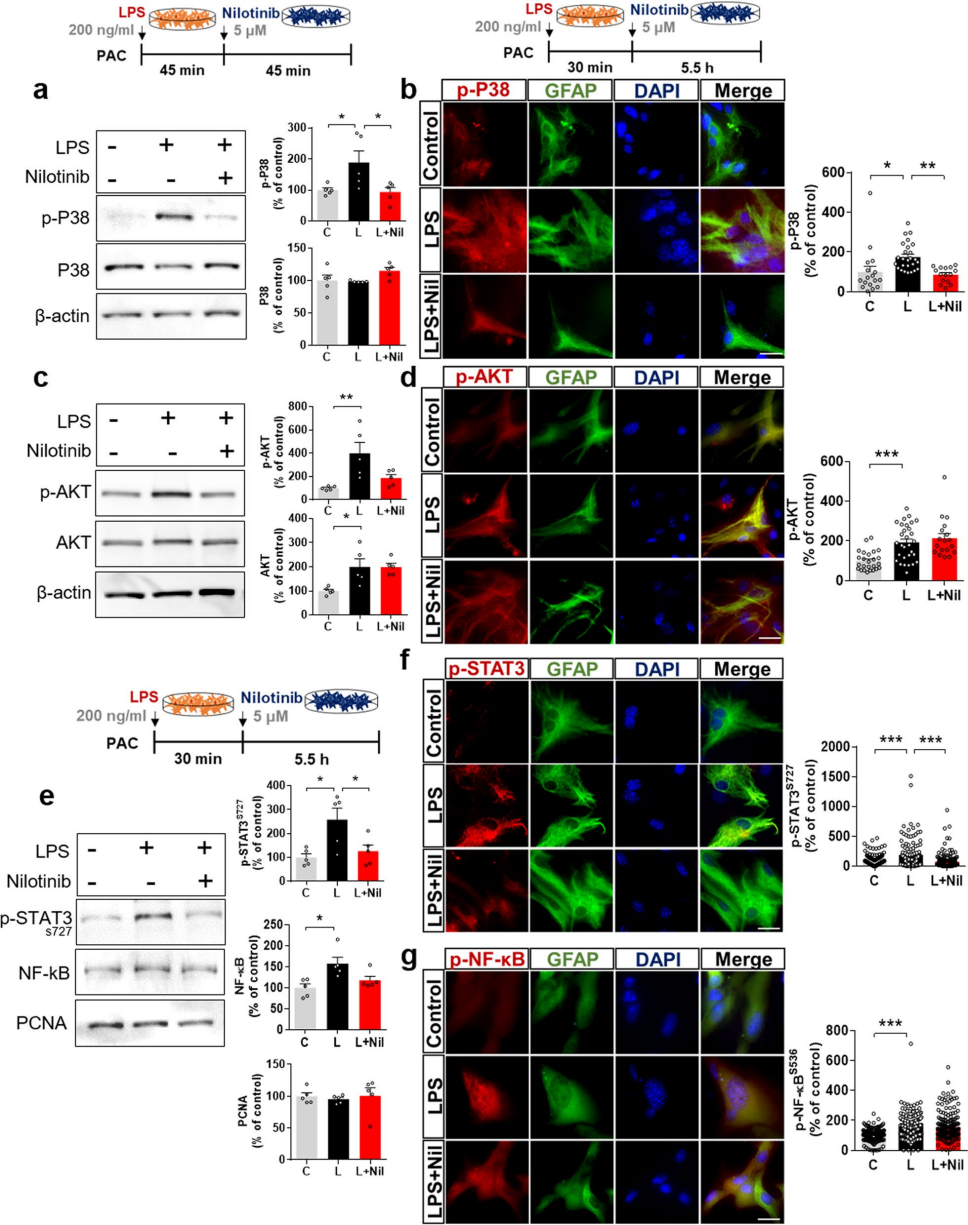
Nilotinib suppresses LPS-stimulated P38/STAT3 signaling in primary astrocytes. **a**, **c**, **e** Western blotting analysis of p-P38/p-AKT signaling and p-STAT3/NF-kB transcription factors in LPS-treated primary astrocytes post-treated with nilotinib as shown (*n* = 5/group). **b**, **d**, **f**, **g** Immunocytochemistry analysis of p-P38, p-AKT, p-STAT3, and p-NF-kB levels in LPS-treated primary astrocytes post-treated with nilotinib as shown (p-P38, C: *n* = 17; L: *n* = 26; L + Nil: *n* = 15, p-AKT, C: *n* = 25; L: *n* = 30; L + Nil: *n* = 18, p-STAT3, C: *n* = 126; L: *n* = 128; L + Nil: *n* = 173, p-NF-kB, C: *n* = 203; L: *n* = 229; L + Nil: *n* = 213). C: control, L: LPS, L + Nil: LPS + nilotinib, *p < 0.05, **p < 0.01, ***p < 0.001, scale bar = 20 μm

To evaluate the effects of nilotinib on LPS-mediated downstream signaling in primary astrocytes, we conducted western blotting and immunocytochemistry with anti-p-P38, anti-p-AKT, anti-p-STAT3, and anti-p-NF-kB or NF-kB antibodies. Nilotinib post-treatment significantly downregulated LPS-evoked p-P38 levels but not p-AKT levels in primary astrocytes (Fig. 6a–d). In addition, nuclear fractionation showed that nilotinib post-treatment significantly downregulated LPS-mediated p-STAT3 levels but not p-NF-kB and total NF-kB levels in primary astrocytes (Fig. 6e–g). These results indicate that nilotinib modulates LPS-evoked astroglial proinflammatory cytokine levels by downregulating P38/STAT3 phosphorylation.

### Nilotinib downregulates LPS-stimulated c-Abl protein levels in wild-type mice

**Fig. 7:**
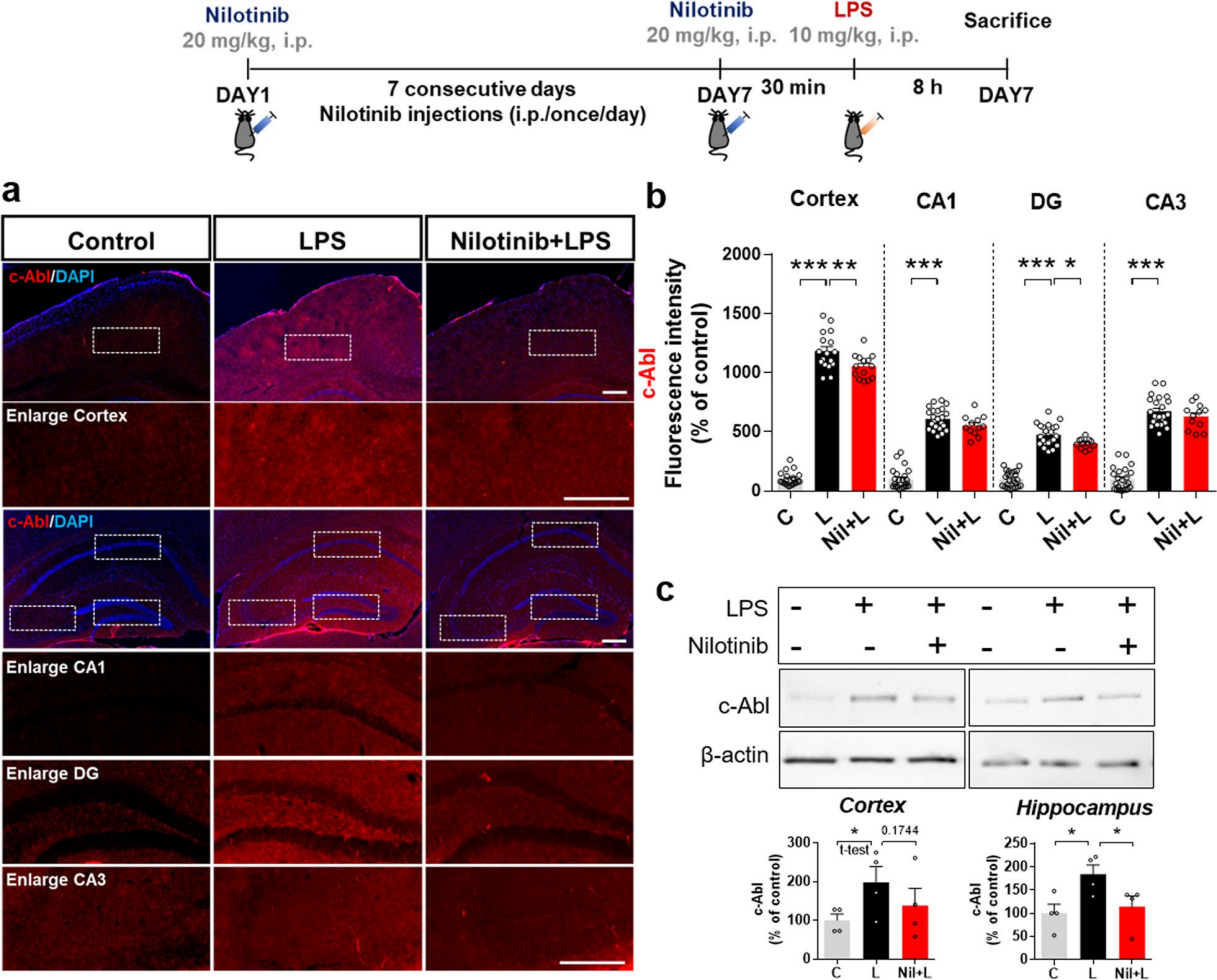
Nilotinib reduces LPS-evoked c-Abl levels in the brain in wild-type mice. **a** Immunofluorescence staining of c-Abl expression in brain slices from wild-type mice injected with vehicle (5% DMSO, 10% PEG and 20% Tween 80 in deionized water) or nilotinib (20 mg/kg, i.p.) daily for 7 days followed by LPS (10 mg/kg, i.p.) or PBS on day 7. **b** Quantification of the data in **a** (*n* = 12–23 brain slices from 3–4 mice/group). **c** Western blotting analysis of c-Abl protein levels in the cortex and hippocampus (*n* = 4/group). *p < 0.05, **p < 0.01, ***p < 0.001. Scale bar = 100 μM

The in vitro studies above confirmed that nilotinib significantly reduces LPS-stimulated c-Abl protein levels in BV2 microglial cells, primary microglia, and primary astrocytes. To determine if these effects also occur in vivo, we injected wild-type mice with 20 mg/kg nilotinib (i.p.) or vehicle (5% DMSO, 10% PEG, and 20% Tween 80 in deionized water) daily for 7 days. On day 7, the mice received a single injection of 10 mg/kg LPS (i.p.) or PBS following the final nilotinib or vehicle injection. Immunofluorescence staining with an anti-c-Abl antibody showed that nilotinib treatment significantly downregulated LPS-evoked c-Abl levels in the cortex and hippocampal DG region but not the hippocampal CA1 and CA3 regions (Fig. 7a, b).

To validate the immunofluorescence results, we conducted western blotting using an anti-c-Abl antibody and found that nilotinib treatment partially or significantly decreased the LPS-induced increase in c-Abl levels in the cortex and hippocampus, respectively (Fig. 7c). These data suggest that nilotinib suppresses LPS-induced c-Abl expression in the brain in wild-type mice.

### Nilotinib modulates LPS-induced changes in microglial activation and microglial/astroglial morphology in wild-type mice

**Fig. 8:**
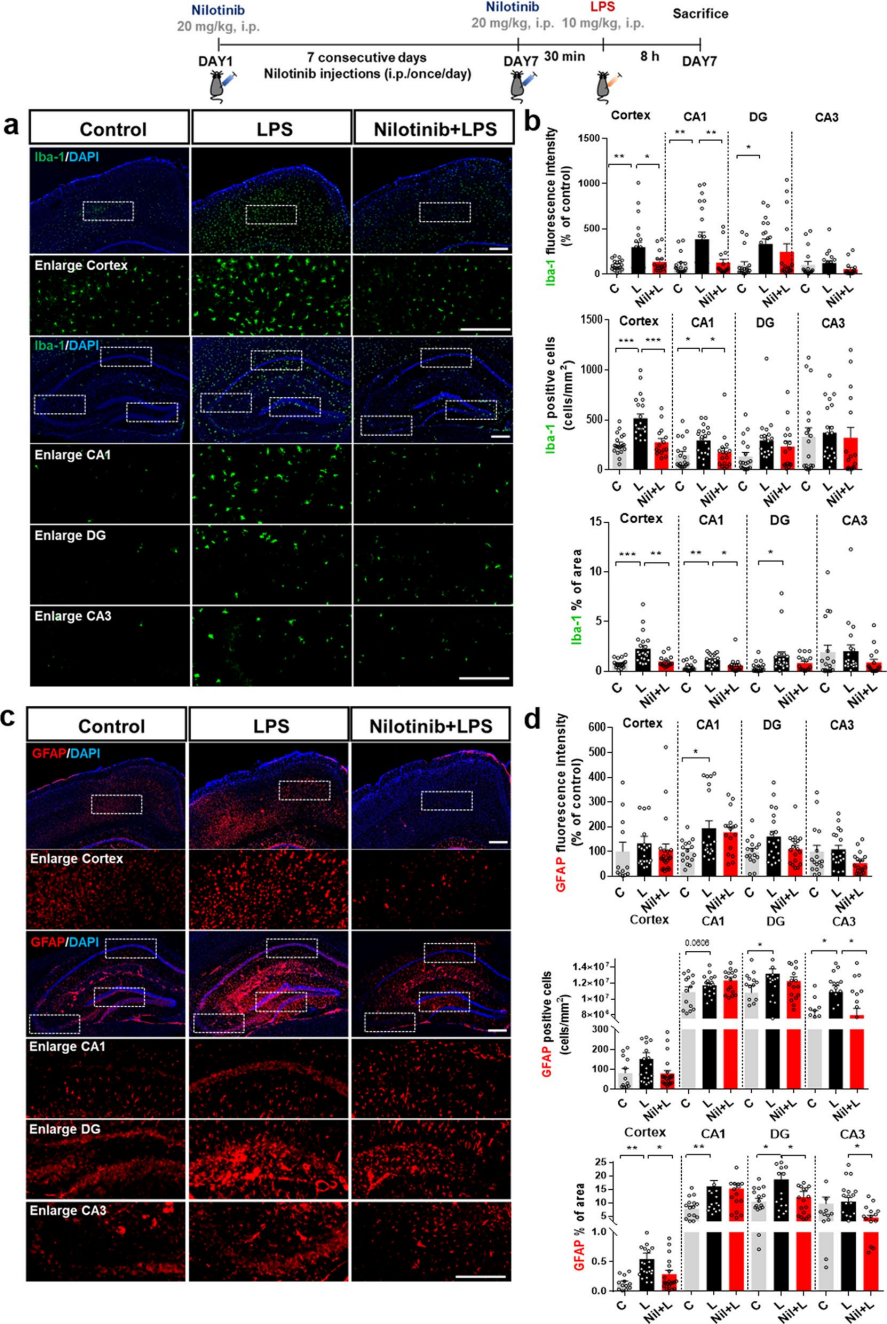
Nilotinib decreases LPS-induced microglial activation/morphology and astrocyte morphology in the brain in wild-type mice. **a**, **c** Immunofluorescence staining of microglial or astrocyte expression in brain slices from wild-type mice injected with vehicle (5% DMSO, 10% PEG and 20% Tween 80 in deionized water) or nilotinib (20 mg/kg, i.p.) daily for 7 days followed by LPS (10 mg/kg, i.p.) or PBS on day 7. **b**, **d** Quantification of data in **a** and **c** (Iba-1, *n* = 15–22 brain slices from 3–4 mice/group; GFAP, *n* = 15–22 brain slices from 3 to 4 mice/group). *p < 0.05, **p < 0.01, ***p < 0.001. Scale bar = 100 μM

As nilotinib altered LPS-induced c-Abl expression in vivo, we next examined the effects of nilotinib on LPS-evoked glial activation, including glial morphology. Wild-type mice were injected with nilotinib and LPS as described above, and immunofluorescence staining was conducted with anti-Iba-1 or anti-GFAP antibodies. Nilotinib significantly decreased the intensity of Iba-1 fluorescent staining, the number of Iba-1-positive cells, and the percent area of Iba-1 staining evoked by LPS in the cortex and hippocampal CA1 region but not in the hippocampal DG and CA3 regions (Fig. 8a, b). In addition, nilotinib significantly suppressed the number of GFAP-positive cells in the hippocampal CA3 region and the percent area of GFAP staining in the cortex and hippocampal DG and CA3 regions in LPS-injected mice (Fig. 8c, d). These data suggest that nilotinib suppresses microglial/astroglial activation in LPS-treated wild-type mice in a brain region-specific manner.

### Nilotinib decreases LPS-induced IL-1β, IL-6, and COX-2 levels in wild-type mice

**Fig. 9:**
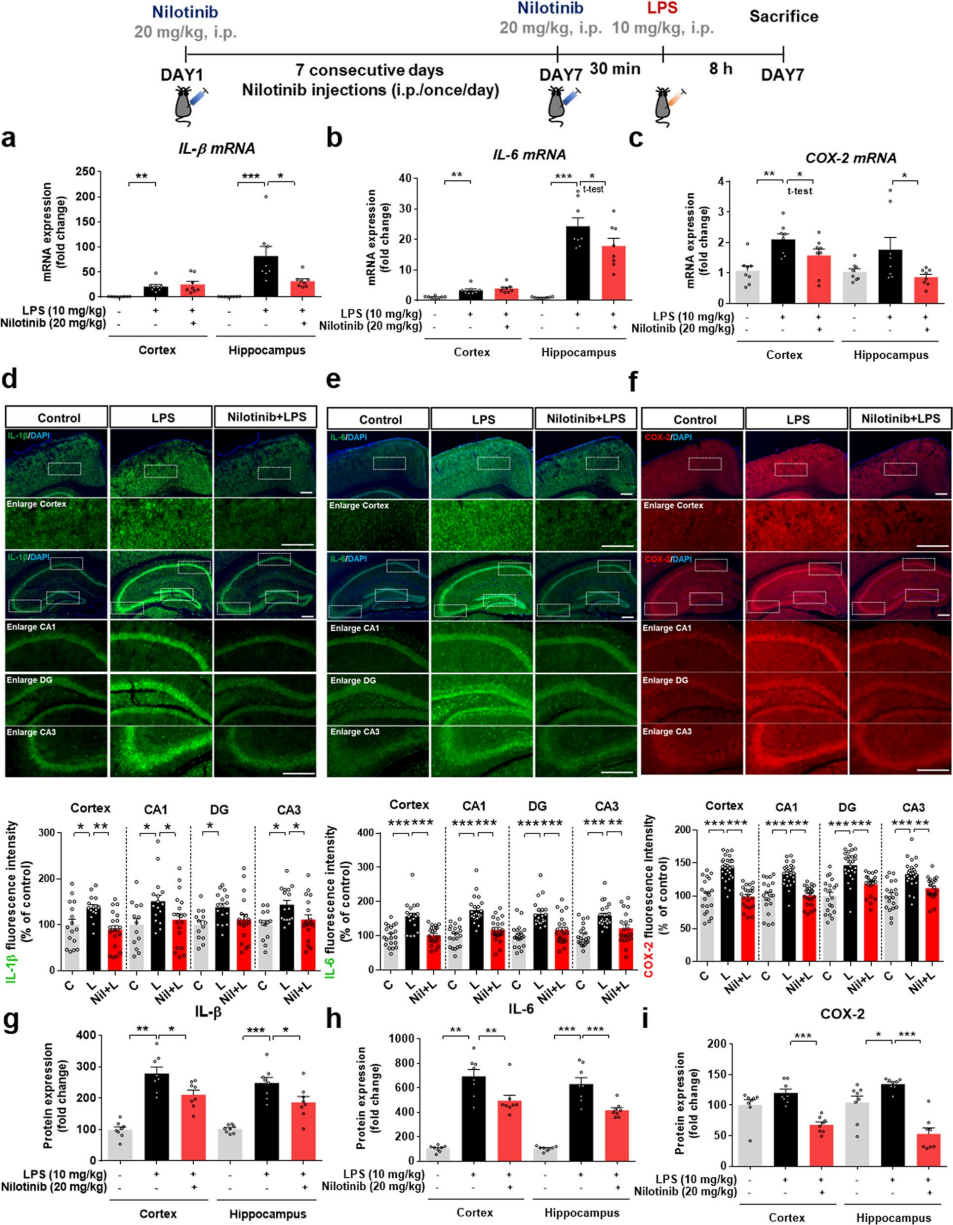
Nilotinib reduces LPS-mediated IL-1β, IL-6, and COX-2 mRNA and protein levels in wild-type mice. **a**–**c** Real-time PCR of IL-1β, IL-6, and COX-2 mRNA levels in the cortex and hippocampus (*n* = 8/group). **d**–**f** Immunofluorescence staining of IL-1β, IL-6, and COX-2 in brain slices from wild-type mice injected with vehicle (5% DMSO, 10% PEG and 20% Tween 80 in deionized water) or nilotinib (20 mg/kg, i.p.) daily for 7 days followed by LPS (10 mg/kg, i.p.) or PBS on day 7. The graphs present the quantification of the raw data from the IF staining in **d**–**f** (IL-1β, *n* = 14–20 brain slices from 3 to 4 mice/group: IL-6, and COX-2, *n* = 20–22 brain slices from 3 to 4 mice/group). **g**–**i** ELISA of IL-1β, IL-6, and COX-2 protein levels in the cortex and hippocampus (*n* = 8/group). *p < 0.05, **p < 0.01, ***p < 0.001. Scale bar = 100 μM

To determine whether nilotinib alters LPS-stimulated proinflammatory cytokine levels in vivo, wild-type mice were injected with nilotinib and LPS as described above, and real-time PCR was performed. Nilotinib significantly diminished LPS-induced IL-1β and IL-6 mRNA levels in the hippocampus but not the cortex (Fig. 9a, b). In addition, nilotinib significantly reduced LPS-induced COX-2 mRNA levels in the cortex and hippocampus (Fig. 9c).

Next, to verify the effects of nilotinib on the protein levels of proinflammatory cytokines in vivo, we conducted immunofluorescence staining with anti-IL-1β, anti-IL-6, and anti-COX-2 antibodies. Nilotinib downregulated LPS-mediated IL-1β levels in the cortex and hippocampal CA1/CA3 regions but not in the hippocampal DG region in wild-type mice (Fig. 9d). IL-6 and COX-2 protein levels in the cortex and hippocampus were significantly reduced by administration of nilotinib in LPS-treated wild-type mice (Fig. 9e, f). Moreover, nilotinib significantly decreased the LPS-induced increases in IL-1β, IL-6, and COX-2 expression in the cortex and hippocampus as measured by ELISA (Fig. 9g–i). These results suggest that nilotinib differentially regulates LPS-evoked mRNA and protein levels of proinflammatory cytokines in a region-specific manner in the brain.

### Nilotinib suppresses LPS-induced P38/STAT3 signaling in wild-type mice

**Fig. 10:**
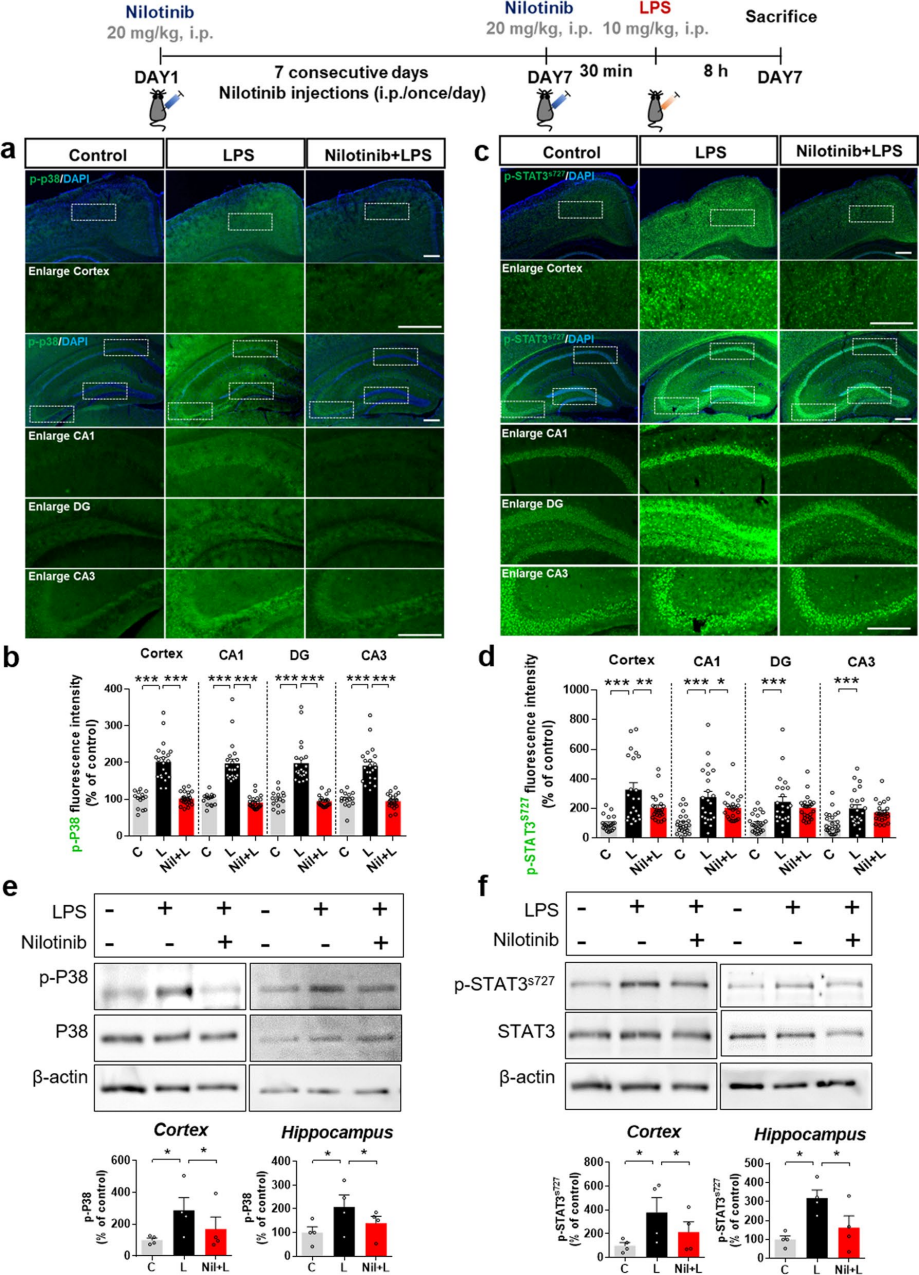
Nilotinib reduces LPS-induced p-P38 and p-STAT3 protein levels in wild-type mice. **a**, **c** Immunofluorescence staining of p-P38 and p-STAT3 in brain slices from wild-type mice injected with vehicle (5% DMSO, 10% PEG and 20% Tween 80 in deionized water) or nilotinib (20 mg/kg, i.p.) daily for 7 days followed by LPS (10 mg/kg, i.p.) or PBS on day 7. **b**, **d** Quantification of data in **a** and **c** (p-P38, *n* = 14–24 brain slices from 3 to 4 mice/group, p-STAT3, *n* = 21–24 brain slices from 3 to 4 mice/group). **e**, **f** Western blotting analysis of p-P38 and p-STAT3 in the cortex and hippocampus in wild-type mice (*n* = 4/group). *p < 0.05, **p < 0.01, ***p < 0.001. Scale bar = 100 μM

Since nilotinib downregulated proinflammatory cytokine mRNA and protein levels in vivo, we next investigated its effects on LPS-associated signaling pathways in the brain. Wild-type mice were injected with nilotinib and LPS as described above, and immunofluorescence staining and western blotting were conducted with anti p-P38 and p-STAT3 antibodies. Immunofluorescence staining showed that nilotinib significantly reduced LPS-induced p-P38 expression in the cortex and hippocampus (Fig. 10a, b) and p-STAT3 levels in the cortex and hippocampal CA1 region but not in the hippocampal DG and CA3 regions (Fig. 10c, d). Consistent with the immunofluorescence results, western blotting showed that LPS significantly upregulated p-P38 and p-STAT3 levels in the cortex and hippocampus in wild-type mice, and these increases were significantly downregulated by nilotinib treatment (Fig. 10e, f). These data suggest that nilotinib contributes to reducing proinflammatory cytokine levels by deactivating P38/STAT3 in response to neuroinflammation in the brain.

### Nilotinib rescues LPS-mediated memory impairments and cortical dendritic spine number in wild-type mice

**Fig. 11:**
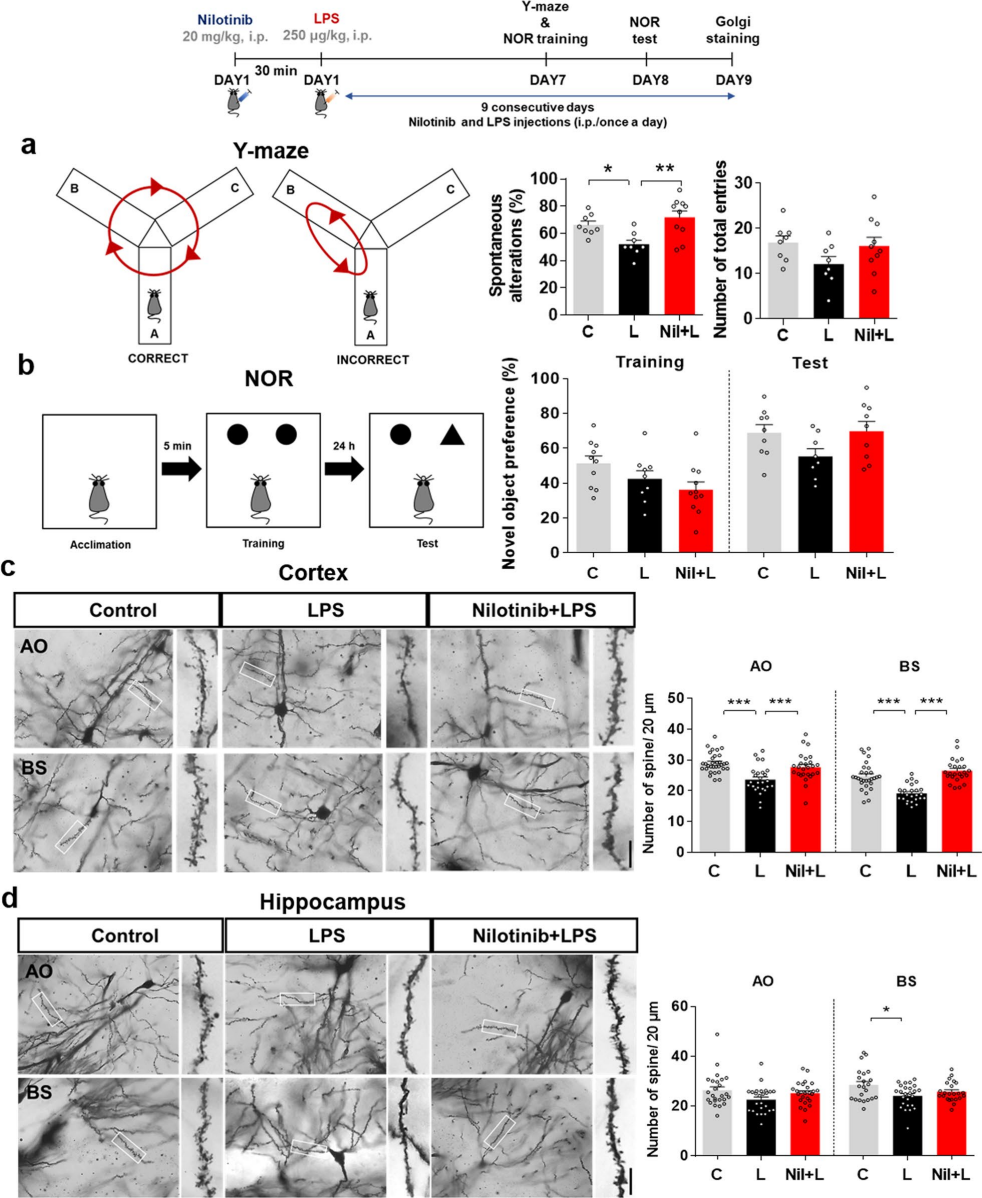
Nilotinib reverses LPS-induced spatial memory impairment and cortical dendritic spine number in wild-type mice. **a**, **b** Y-maze and novel object recognition (NOR) tests were performed on days 7 and 8, respectively. Spontaneous alternations and number of total entries are represented in **a**, and object preference is shown in **b** (*n* = 8–11 mice/group). **c**, **d** After the behavior experiments, Golgi staining was performed, and dendritic spine number was measured in the cortical and hippocampal AO and BS regions (*n* = 25–26 neurons from 4 to 5 mice/group). *p < 0.05, **p < 0.01, ***p < 0.001. Scale bar = 10 μM

Since nilotinib altered LPS-mediated neuroinflammatory responses in vivo, we next examined whether nilotinib modulates neuroinflammation-induced memory impairment by conducting Y-maze and NOR tests of wild-type mice injected with nilotinib and LPS as described above. Importantly, nilotinib recovered the LPS-induced decrease in spontaneous alternations but did not alter novel object preference in the NOR test (Fig. 11a, b).

Golgi staining to assess the effect of nilotinib on dendritic spine formation showed that nilotinib significantly rescued LPS-mediated dendritic spine loss in the cortical apical oblique (AO) and basal shaft (BS) regions (Fig. 11c). However, nilotinib did not alter LPS-mediated dendritic spine loss in the hippocampal AO and BS regions (Fig. 11d). These data suggest that nilotinib ameliorates neuroinflammation-linked short-term spatial memory impairment and dendritic spine loss in wild-type mice.

## Discussion

**Fig. 12:**
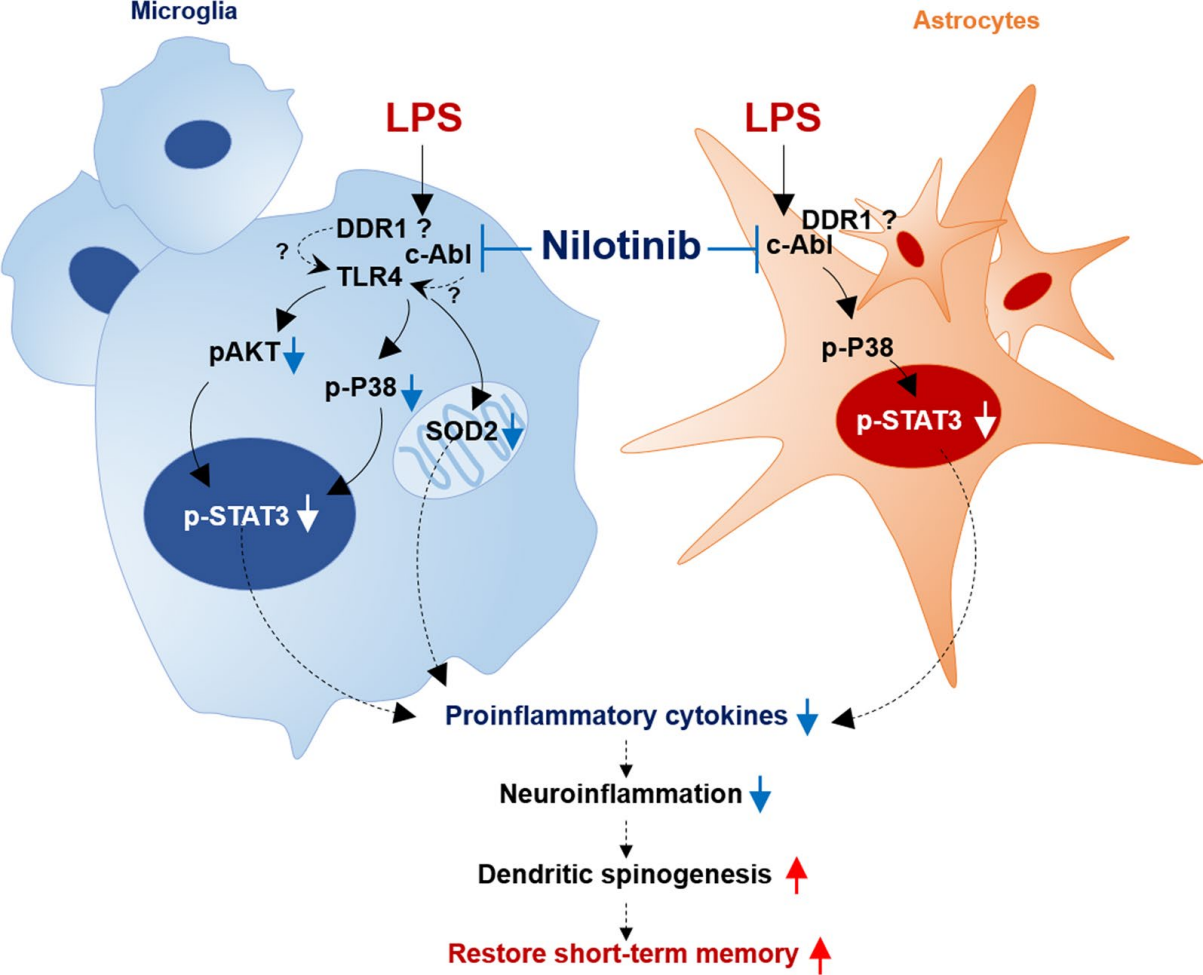
Diagram of the effects of nilotinib on LPS-stimulated neuroinflammatory responses and cognitive function. In microglial cells, nilotinib alters LPS-induced proinflammatory cytokine levels via AKT/P38/STAT3 activation and/or in a Sod2-dependent manner. In primary astrocytes, nilotinib downregulates the LPS-stimulated increase in proinflammatory cytokine levels by inhibiting the P38/STAT3 axis. In wild-type mice, nilotinib reduces LPS-induced gliosis, proinflammatory cytokine levels, and downstream P38/STAT3 signaling. Importantly, nilotinib rescues the short-term memory impairment and decrease in dendritic spinogenesis induced by LPS in wild-type mice. The ability of nilotinib to alter LPS-induced gliosis in vitro/in vivo and to modulate neuroinflammation-linked behavior suggests that nilotinib may have potential utility as a therapeutic drug for neuroinflammation/cognitive-associated disease

In the present study, we demonstrated that nilotinib reduced LPS-mediated c-Abl protein levels and proinflammatory cytokine levels via modulation of the p-P38/p-STAT3 axis in BV2 microglial cells, primary microglia, and astrocytes. In addition, we found that the effects of nilotinib on LPS-induced microglial IL-6 mRNA expression were Sod2 dependent. In LPS-treated wild-type mice, nilotinib injection downregulated microglial/astroglial activation and morphology in a brain region-dependent manner. Additionally, nilotinib significantly reduced the LPS-induced increases in IL-1β, IL-6, and COX-2 expression in the brain by modulating P38/STAT3 phosphorylation. Importantly, nilotinib rescued the LPS-mediated impairments of short-term spatial memory and cortical dendritic spine number in wild-type mice. The ability of nilotinib to alter LPS-induced gliosis in vitro/in vivo and to modulate neuroinflammation-linked behavior suggests that nilotinib may have potential utility as a therapeutic drug for neuroinflammation-associated disease (Fig. 12).

In this study, we used LPS to construct a neuroinflammation-associated disease model because several studies have demonstrated that LPS promotes neuroinflammation and neurodegeneration in vivo (e.g., AD and PD) [27–30]. For example, chronic LPS injection (i.c.v.) significantly increases β-APP mRNA in the nucleus basalis of rats [27]. In addition, 7 days of administration of LPS increases Aβ 1–42 expression and triggers AD-like neuronal degeneration in the cortex and hippocampus in aged rats [28]. In ICR albino mice, repeated injection of LPS (i.p.) 3 or 7 times activates astroglial β- or γ-secretases, resulting in Aβ 1–42 accumulation in the cerebral cortex and hippocampus [29]. Tau phosphorylation is increased in wild-type mice injected with LPS, an AD-like disease model [30]. Moreover, LPS injection (daily for 4 days, 1 mg/kg, i.p.) reduces neuronal survival, dopamine levels, and glial activation in the substantia nigra (SN) and produces deficits in locomotor activity in C57BL/6J male mice [31]. Injection of C57BL/6 mice with LPS (once weekly for 5 weeks or one monthly for 5 months) causes TH-positive neuronal loss in the substantia nigra (SN) and α-synuclein aggregation, resulting in motor impairment [32]. Given these observations, we used an LPS-injected mouse model in the present study to evaluate the effects of nilotinib on neuroinflammatory responses and neuroinflammation/neurodegenerative disease-linked cognitive function.

Nilotinib inhibits the activity of the chimeric Bcr-Abl enzyme. This fusion of the Bcr and Abl proteins is the product of reciprocal translocation between chromosomes 9 and 22 and leads to constitutively active tyrosine kinase-linked signaling, resulting in impaired DNA repair and genomic instability in cancer cells [33]. C-Abl has been shown to contribute to the modulation of proinflammatory cytokine levels in vitro [23, 34, 35]. In BV2 microglial and RAW 264.7 cells, nilotinib reduces LPS-induced increases in c-Abl mRNA and protein levels [34, 35]. Moreover, nilotinib suppresses LPS-mediated increases in COX-2, IL-1β, IL-6, and TNF-α proinflammatory cytokines in BV2 microglial cells [24]. Transfection of c-Abl siRNA significantly reduces levels of the transcription co-factor p-IκB in primary microglia and astrocytes [34]. However, the effects of nilotinib on Bcr-Abl-linked pathways regulating proinflammatory cytokines in microglia and astrocytes have not been investigated, nor have in vivo studies been performed. The present study of the effects of nilotinib on LPS-evoked neuroinflammatory responses found that nilotinib significantly reduced LPS-mediated c-Abl levels and proinflammatory cytokine (i.e., COX-2, IL-1β, and IL-6) levels in BV2 microglial cells, primary microglia, and primary astrocytes by regulating P38 and P38-linked STAT3 activation (Figs. 1, 2, 4, 5, and 6).

Toll-like receptors (TLRs), a key component of innate immune signaling, are the predominant receptors involved in the LPS-mediated immune response. TLR subfamily 4 (TLR4) is activated by pathogenic molecules such as LPS. Several studies have reported that drugs that reduce proinflammatory cytokine levels in vitro and in vivo via TLR4-dependent signaling pathways hold potential for treating neuroinflammation-linked neurodegenerative diseases [20, 21, 36, 37]. For instance, MAO inhibitor treatment significantly reduces the LPS-induced increase in IL-1β and IL-6 mRNA levels, and IL-6 but not IL-1β levels are partially dependent on TLR4 signaling in BV2 microglial cells [37]. In addition, the VEGFR antagonist sorafenib and ATP-sensitive potassium channel blocker gliquidone reduce proinflammatory cytokine levels in BV2 microglia cells via TLR4-linked signaling pathways [21, 36]. In this study, treatment of BV2 microglial cells with LPS, TLR4 inhibitor, and nilotinib significantly downregulated LPS-evoked IL-1β levels compared with treatment with LPS and TLR4 inhibitor but not treatment with LPS and nilotinib, indicating that the effects of nilotinib on IL-1β levels are independent of TLR4 (Fig. 2). However, we observed that nilotinib reduced LPS-evoked COX-2 mRNA levels in a TLR4-dependent manner (Fig. 2). Thus, it is possible that nilotinib alters LPS-induced neuroinflammatory responses via TLR4-independent or -dependent c-Abl/DDR or downstream P38/STAT3 signaling. A future study will address these possibilities. Overall, nilotinib affects LPS-mediated proinflammatory cytokine levels and downstream P38/STAT3 signaling in vitro.

Neuroinflammatory cells, including microglia, are sources of reactive species that can regulate the release of proinflammatory cytokines [38]. Proinflammatory cytokine release is closely related to SOD2 activation in response to oxidative stress in vivo and in vitro [17, 39, 40]. For example, LPS induces oxidative stress [i.e., ROS, nitric oxide (NO), NADPH oxidase (NOX)] and increases Sod2 mRNA and protein levels in BV2 microglial cells and rat primary microglia [17, 25]. In rat lung tissue, LPS injection significantly increases Sod2 gene expression, and nilotinib suppresses LPS-induced increases in SOD2 and glutathione peroxidase (GSH), which regulates nitrite/nitrate (NO_2_^−^/NO_3_^−^) concentrations [40]. In line with our data in Fig. 3d, Ishihara and co-workers found that Sod2 knockdown significantly increases the induction of TNF-α and IL-1β gene expression by LPS in rat primary microglia [17]. However, the potential link between Sod2-associated expression of proinflammatory cytokines and tyrosine kinase activation has not been clarified. In the present study, we found that knockdown of Sod2 was required for nilotinib to reduce LPS-evoked IL-6 gene expression in BV2 microglial cells but had no effect on IL-1β and COX-2 expression (Fig. 3). Taken together, our findings and the literature suggest that nilotinib downregulates IL-6 expression in a Sod2-dependent manner in LPS-treated BV2 microglial cells. Future studies will examine the possibility that nilotinib affects other neuroinflammation-associated molecular targets to alter LPS-induced IL-1β and COX-2 levels.

Given the effects of nilotinib on LPS-mediated proinflammatory cytokine expression in vitro (microglia and astrocytes), we investigated whether nilotinib modulates neuroinflammation in vivo and its underlying mechanism. Previous work has shown that administration of 25 mg/kg nilotinib (p.o., 7 days) significantly decreases LPS-induced increases in proinflammatory cytokine COX-2 and IL-1β levels as well as Iba-1 immunoreactivity in the substantia nigra part compacta (SNpc) in a mouse model of PD [24]. Similarly, treatment with 30 mg/kg nilotinib (p.o., 5 months) reduces α-synuclein preformed fibril (PFF)-induced activation of Iba-1 and GFAP immunoreactivity in the SNpc in a PD mouse model [41]. In adult male rats, the pentylenetetrazol (PTZ)-induced increase in GFAP-immunopositive cells in the hippocampal CA1 region is attenuated by nilotinib injection [42].

Microgliosis or astrogliosis can regulate proinflammatory cytokine levels, but LPS or drugs may impact proinflammatory cytokine expression before gliosis occurs. For instance, the proinflammatory cytokines IL-1β, TNF-α, and IL-6 increase significantly within 3 h after injection of a low (1 mg/kg) or high (5 mg/kg) dose of LPS, whereas gliosis occurs 24–72 h after LPS injection (1 or 5 mg/kg) in LPS-induced mice model [43, 44]. In addition, 10 mg/kg LPS (single injection, i.p.) promotes gliosis 6 h after LPS injection in the cortex and hippocampus of mice [18]. In the present study, nilotinib significantly decreased proinflammatory cytokine levels in the brains of LPS-injected mice but did not alter microgliosis in the DG/CA3 region (Figs. 8, 9). These data suggest that proinflammatory cytokines respond to LPS before gliosis is evoked. Based on the literature and our findings, it is possible that nilotinib treatment first downregulates LPS-mediated proinflammatory cytokine levels via TLR4 signaling and/or other neuroinflammation-associated molecular targets, which in turn leads to effects on microgliosis and astrogliosis. Another possibility is that nilotinib modulates LPS-induced proinflammatory cytokine levels and microgliosis/astrogliosis via bidirectional or multidirectional pathways depending on the brain region (hippocampus CA1, CA3 vs. DG region). Future studies will address these possibilities.

Nilotinib blocks P38 MAPK activation in leukocytes in the liver in wild-type mice and AKT/STAT3 phosphorylation in human melanoma cells [45, 46]. However, whether the effects of nilotinib on LPS-stimulated proinflammatory responses and downstream signaling in the cortex and hippocampus are related to cognitive consolidation and learning and memory integration remains unknown. Here, we found for the first time that administration of 20 mg/kg nilotinib (i.p. daily for 7 days) suppressed the LPS-induced increases in Iba-1, GFAP, and IL-1β expression and P38/STAT3 activation in the cortex and hippocampus, which are the core of learning and memory consolidation in wild-type mice (Figs. 8, 9, 10). Therefore, our findings and previous works suggest that nilotinib suppresses cortical and hippocampal neuroinflammatory responses via modulation of downstream P38/STAT3 signaling in LPS-injected wild-type mice. It is possible that nilotinib affects LPS-mediated proinflammatory cytokine production in glia by suppressing other tyrosine kinases and transcription factors, such as JAK2/ERK1/2 or CREB/mTOR, which are known to be involved in Bcr-Abl-mediated downstream signaling. The potential effects of nilotinib on other LPS-associated MAPK signaling pathways and transcription factors linked to c-Abl tyrosine kinase in vivo will be investigated in future work.

Several recent studies have suggested that nilotinib also inhibits DDR1 (discoidin domain receptor 1), a tyrosine kinase belonging to the DDR family that is overexpressed in microglia in response to collagen [10]. DDR1 is expressed in BV2 microglia and primary microglia, and nilotinib treatment decreases the increases in the proinflammatory cytokines COX-2 and iNOS induced by collagen in BV2 microglial cells [10]. In DDR1^−/−^ mice, the increases in kidney IFNγ, IL-23, and TNF-α levels induced by unilateral ureteral obstruction (UUO) are significantly reduced [47]. In addition, treatment with the MAPK inhibitor SB203580, the JNK inhibitor SP600125, or the ERK inhibitor PD98059 significantly decreases collagen-induced NF-kB phosphorylation via P38/JNK activation in BV2 microglia and primary microglia [10]. This evidence suggests that nilotinib affects LPS-induced neuroinflammatory responses by inhibiting DDR1, and the effects of nilotinib on DDR1-mediated neuroinflammatory responses will be examined in a future study.

Interestingly, clinical studies have revealed a strong association between DDR1 and AD/PD pathology. For example, DDR1/2 are upregulated in post-mortem PD and AD brains, and knockdown of DDR1 reduces levels of Aβ_42_, α-synuclein, and tau in APP-overexpressing mice [12, 14, 48]. In B35 cells, lentiviral-induced shDDR1 and co-treatment with shDDR1 and nilotinib significantly suppress Aβ_42_ levels [48]. In addition, nilotinib treatment (150 mg and/or 300 mg, p.o., for 26 weeks) significantly reduces Aβ burden, α-synuclein oligomers and phospho-tau-181 in the CSF in AD patients [12, 14]. A phase 2 randomized clinical study reported that nilotinib administration (150 mg, p.o., for 12 months) significantly increases the dopamine metabolites homovanillic acid and 3,4-dihydroxyphenylacetic acid in the CSF of AD and PD patients [12, 14]. Moreover, whole-genome sequencing of miRNAs in the CSF of PD patients treated with nilotinib (150 mg, 300 mg, p.o. for 12 months) demonstrated that nilotinib reduces factors associated with BBB degradation and restores autophagy-linked factors, markers of neuroinflammation, and micro-environmental markers of angiogenesis [49]. Taken together, these findings indicate that nilotinib blocks DDR1 activity and downregulates AD and PD pathology. Therefore, it will be interesting to identify the links between DDR1 and AD/PD pathology in nilotinib treatment and their underlying mechanisms in vitro and in vivo.

A correlation between neuroinflammation and memory impairments has been proposed, as the levels of proinflammatory molecules such as IL-1 and IL-1β in the brain are associated with AD and AIDS-mediated dementia [50–52]. Peripheral and intracerebral injection of IL-1β and chronic IL-1β overexpression in the hippocampus impair spatial and contextual memory in mice [51, 53, 54]. In a mouse model of AD, nilotinib decreases Aβ-plaque-induced increases in proinflammatory cytokine levels in the hippocampus, and in patients with AD or PD, nilotinib improves learning/memory behavior [12–14, 23]. Nilotinib significantly restores synaptic function in human cellular AD models by increasing the levels of the synaptic proteins Rab3A and SV2B [55]. Six months of treatment with nilotinib (150 mg and 300 mg) improves cognition in patients with Parkinson’s disease compared to baseline [13], whereas Cludiani et al. reported that leukemia patients treated with nilotinib experience memory dysfunction 4–6 months after cessation [56]. In addition, early and chronic administration of nilotinib in Tg2576 AD model mice reduces Aβ accumulation by blocking DA neuron degradation and preventing morphological changes in the ventral tegmental area (VTA) [57].

Several studies have suggested that increased cortical dendritic spine number primes neurons for better memory [58, 59]. For example, Frank and co-workers reported that spine turnover drives the localization of new spine cluster formation and modulates the neural network, thus influencing storage capacity, learning and memory [58]. Additionally, increased cortical dendritic spine density increases network of synapses and improves spatial learning tasks linked to the hippocampus [59]. Although spatial learning requires a network of brain areas, the prefrontal cortex is obviously an important part of cognitive memory (including spatial working memory) [60]. Based on the above, we hypothesized that nilotinib could restore LPS-induced deficits in cognitive function in vivo by inhibiting neuroinflammation and/or neuroinflammation-associated factors. We found that nilotinib treatment (daily for 7 days, 20 mg/kg, i.p.) significantly restored the LPS-induced decrease in spontaneous alterations, but did not alter novel object preference. Interestingly, nilotinib significantly rescued dendritic spine number in the cortical AO and BS regions. Therefore, we speculate that nilotinib contributes to improved spatial working memory by restoring cortical spine formation (Fig. 11). Taken together, these data suggest that nilotinib ameliorates LPS-mediated memory impairments by suppressing neuroinflammation and/or neuroinflammation-related molecular targets in vivo.

However, this study is subject to limitations. First, nilotinib is a multi-target tyrosine kinase inhibitor (e.g., Bcr-Abl, DDR1) and thus affects various tyrosine kinase-linked molecular targets and signaling cascades in microglia and astrocytes in response to neuroinflammation. Therefore, the potential role of multi-tyrosine kinase-linked mechanisms in the effects of nilotinib on LPS-induced neuroinflammatory responses (directly and/or indirectly via Bcr-Abi and/or DDR1) needs to be addressed in a future study. Second, in this study we found that daily nilotinib treatment (20 mg/kg, i.p.) for 7 days rescued LPS-mediated short-term memory impairment as well as cortical dendritic spine loss. It is possible that longer treatment durations (e.g., daily for 1 month) and/or higher doses of nilotinib (30 mg/kg) may further improve short- and long-term memory, which will be examined in future work. Third, the molecular mechanisms by which nilotinib modulates cognitive function remain to be identified.

This study is the first to explore the mechanisms underlying the effects of nilotinib on LPS-induced neuroinflammation and cognitive function (including dendritic spinogenesis) in vitro and in vivo and thus provides several novel findings. First, we showed that nilotinib decreases LPS-induced proinflammatory cytokine IL-6 levels in a Sod2-dependent manner in BV2 microglial cells and primary microglia. Second, we found that nilotinib downregulates LPS-stimulated proinflammatory cytokine levels and P38/STAT3 signaling in primary astrocytes. Third, we demonstrated that nilotinib suppresses phosphorylation of the LPS-induced transcription factor STAT3 to downregulate neuroinflammatory responses in primary microglia, primary astrocytes, and in vivo (cortex and hippocampus, the core of learning and memory processing). Fourth, we observed that nilotinib alters LPS-mediated microglial activation/morphology, astrocyte morphology, and proinflammatory cytokine levels in the cortex and hippocampus in vivo. Finally, we demonstrated that nilotinib can rescue the impairments of short-term memory and cortical dendritic spine formation induced by injection of a low dose of LPS (250 µg/mL) or chronic LPS administration (daily for 7 days, i.p.) in vivo. Taken together, our results from a neuroinflammation-associated disease model (i.e., LPS) support novel inhibitory effects of nilotinib on neuroinflammatory responses and provide insights into nilotinib’s molecular mechanisms of action.

## Conclusions

The current study explored the impact of nilotinib on LPS-stimulated neuroinflammatory responses in vitro and in vivo. In BV2 microglial cells, primary microglia, and primary astrocytes, nilotinib modulated LPS-induced proinflammatory cytokine levels by inhibiting p38/STAT3/Sod2. In wild-type mice, nilotinib reduced LPS-induced microglial activation/morphology and/or astroglial morphology, in addition to suppressing LPS-mediated proinflammatory cytokine IL-1β, IL-6, and COX-2 levels and downstream P38/STAT3 signaling. Importantly, nilotinib treatment rescued LPS-induced short-term memory impairment and cortical dendritic spine number in vivo. In summary, these data suggest that nilotinib modulates LPS-induced neuroinflammatory responses, cognitive function, and dendritic spine formation.

## Supplementary Information

**Additional file 1: Figure S1.** Nilotinib does not alter LPS-mediated nuclear p-NF-kB levels in BV2 microglial cells. **a** Western blotting analysis of NF-kB in LPS-treated BV2 microglial cells post-treated with nilotinib as shown (*n* = 5/group). **b** Immunocytochemistry of CD11b and p-NF-kB in LPS-treated BV2 microglial cells post-treated with nilotinib as shown. The graph shows the quantification of the data in the left panel (C, *n* = 546; L, *n* = 263; L + Nil, *n* = 342). C: control, L: LPS, L + Nil: LPS + Nilotinib, ***p < 0.001, scale bar = 20 μm. **Figure S2.** Nilotinib does not affect LPS-induced NLRP3, NRF2, SOD1, and Sirt3 levels in BV2 microglial cells. **a-b** Real-time PCR analysis of NLRP3, NRF2, SOD1, and Sirt3 levels in LPS-treated BV2 microglial cells post-treated with nilotinib as shown (*n* = 6/group). C: control, L: LPS, L + Nil: LPS + Nilotinib, ***p < 0.001. **Table S1.** One-way ANOVA (Tukey’s test) and significance of the results of the in vitro experiments in this study. **Table S2.** *t*-Tests or one-way ANOVA (Tukey’s test) and significance of the results of the in vivo experiments in this study.

## Data Availability

All data generated and/or analyzed during this study are included in this article.

## Abbreviations

SOD2: Superoxide dismutase 2
COX-2: Cyclooxygenase-2
IL-1β: Interleukin-1β
IL-6: Interleukin-6
LPS: Lipopolysaccharide
TLR4: Toll-like receptor 4
TNF-α: Tumor necrosis factor-α
STAT3: Signal transducer and activator of transcription 3
AO: Apical oblique
BS: Basal shift
AD: Alzheimer’s disease
PD: Parkinson’s disease
CML: Chronic myelogenous leukemia
Bcr: Breakpoint cluster region protein
Abl: ABL proto-oncogene 1
CNS: Central nervous system
