# Supplementary material for: Nilotinib modulates LPS-induced cognitive impairment and neuroinflammatory responses by regulating P38/STAT3 signaling
Source: J Neuroinflammation. 2022 Jul 15;19:187. doi: 10.1186/s12974-022-02549-0 (PMC9288088; doi:10.1186/s12974-022-02549-0)
Supplement: Supplementary file 1 — Additional file 1: Figure S1. Nilotinib does not alter LPS-mediated nuclear p-NF-kB levels in BV2 microglial cells. a Western blotting analysis of NF-kB in LPS-treated BV2 microglial cells post-treated with nilotinib as shown (n = 5/group). b Immunocytochemistry of CD11b and p-NF-kB in LPS-treated BV2 microglial cells post-treated with nilotinib as shown. The graph shows the quantification of the data in the left panel (C, n = 546; L, n = 263; L + Nil, n = 342). C: control, L: LPS, L + Nil: LPS + Nilotinib, ***p < 0.001, scale bar = 20 μm. Figure S2. Nilotinib does not affect LPS-induced NLRP3, NRF2, SOD1, and Sirt3 levels in BV2 microglial cells. a-b Real-time PCR analysis of NLRP3, NRF2, SOD1, and Sirt3 levels in LPS-treated BV2 microglial cells post-treated with nilotinib as shown (n = 6/group). C: control, L: LPS, L + Nil: LPS + Nilotinib, ***p < 0.001. Table S1. One-way ANOVA (Tukey’s test) and significance of the results of the in vitro experiments in this study. Table S2. t-Tests or one-way ANOVA (Tukey’s test) and significance of the results of the in vivo experiments in this study. [file 12974_2022_2549_MOESM1_ESM.docx]

**Nilotinib modulates LPS-induced cognitive impairment and neuroinflammatory responses by regulating P38/STAT3 signaling**

Jieun Kim^a^, Hyun-ju Lee^a^, Jin-Hee Park^a,b^, Byung-Yoon Cha^c^, Hyang-Sook Hoe^a,b *^

^a^Department of Neural Development and Disease, Korea Brain Research Institute (KBRI), 61, Cheomdan-ro, Dong-gu, Daegu, Korea, 41062; ^b^Department of Brain and Cognitive Sciences, Daegu Gyeongbuk Institute of Science & Technology, Daegu 42988, Korea; ^c^PharmacoRex Co., Ltd., 20 Techno 1-ro, Yuseong-gu, Daejeon, Korea, 34016

Jieun Kim: jieunkim@kbri.re.kr

Hyun-ju Lee: hjlee@kbri.re.kr

Jin-Hee Park: mingmeng1005@kbri.re.kr

Byung-Yoon Cha: pmrceo@pharmacorex.co.kr

Hyang-Sook Hoe: sookhoe72@kbri.re.kr

*Corresponding author

Hyang-Sook Hoe, Ph.D., Department of Neural Development and Disease, Korea Brain Research Institute (KBRI), 61, Cheomdan-ro, Dong-gu, Daegu, Korea 41068

E-mail: sookhoe72@kbri.re.kr


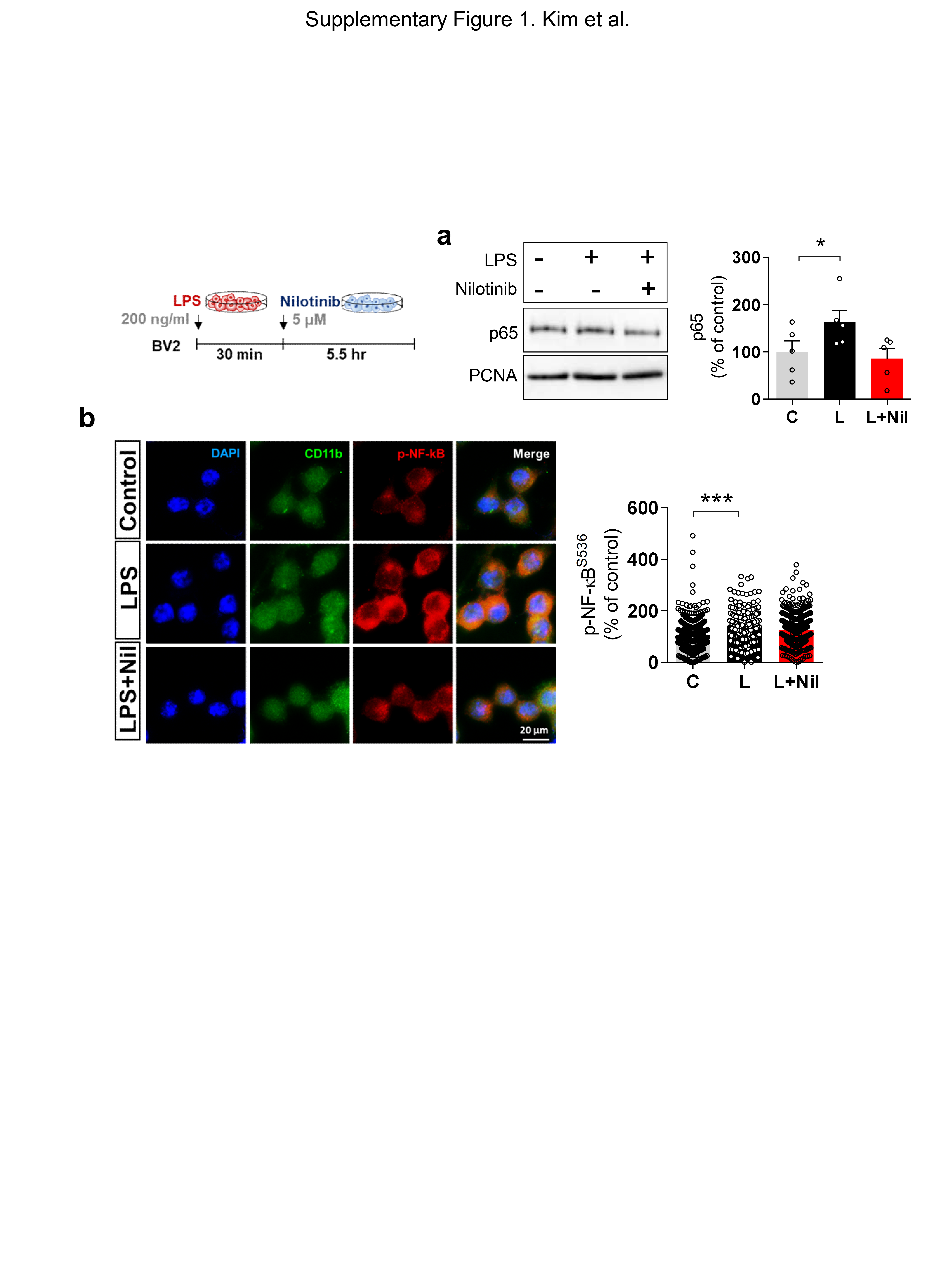


**Figure S1.** Nilotinib does not alter LPS-mediated nuclear p-NF-kB levels in BV2 microglial cells. **a** Western blotting analysis of NF-kB in LPS-treated BV2 microglial cells post-treated with nilotinib as shown (n = 5/group). **b** Immunocytochemistry of CD11b and p-NF-kB in LPS-treated BV2 microglial cells post-treated with nilotinib as shown. The graph shows the quantification of the data in the left panel (C, n = 546; L, n = 263; L+Nil, n = 342). C: control, L: LPS, L+Nil: LPS+Nilotinib, ***p < 0.001, scale bar = 20 μm.


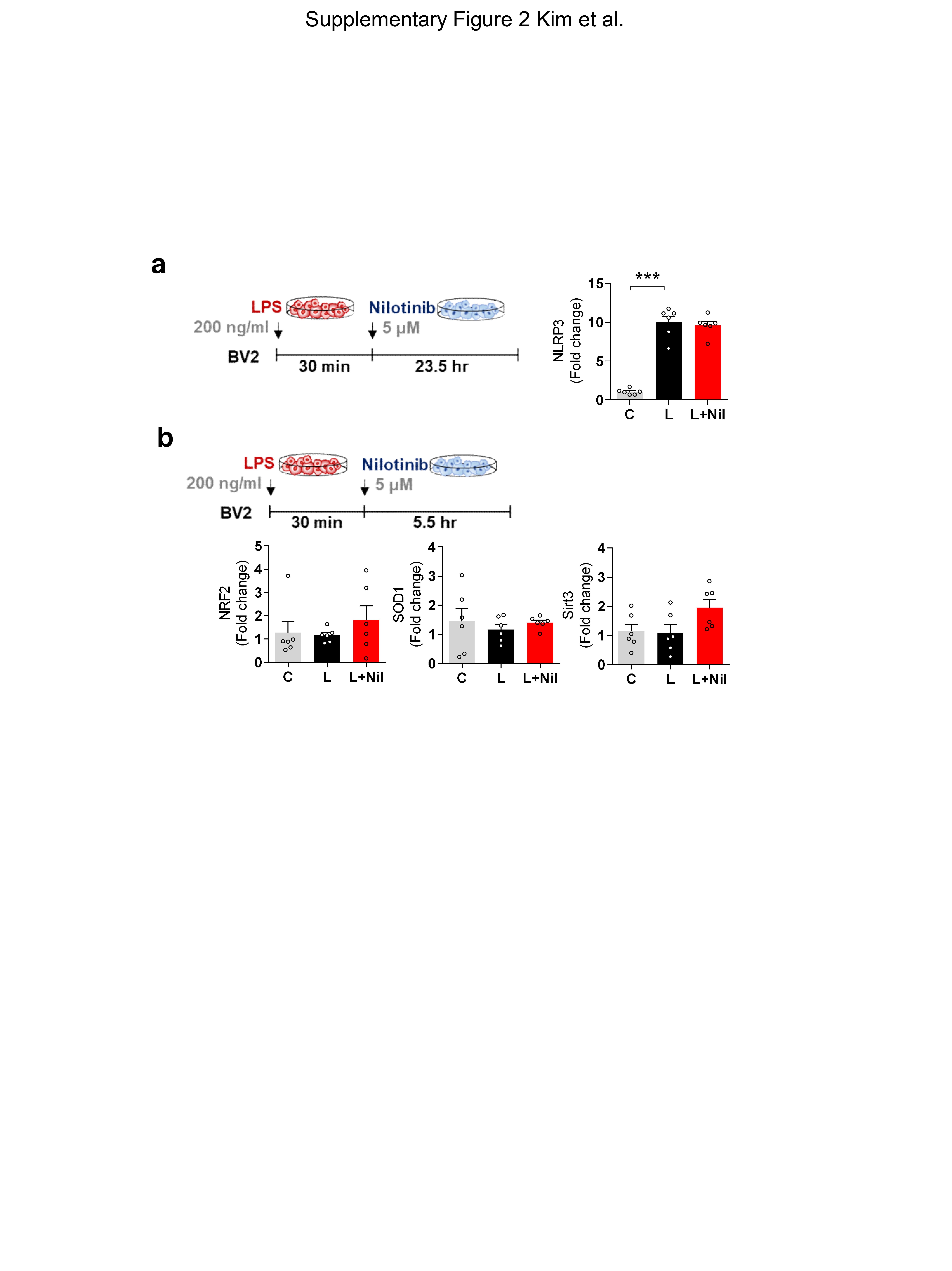


**Figure S2.** Nilotinib does not affect LPS-induced NLRP3, NRF2, SOD1, and Sirt3 levels in BV2 microglial cells. **a-b** Real-time PCR analysis of NLRP3, NRF2, SOD1, and Sirt3 levels in LPS-treated BV2 microglial cells post-treated with nilotinib as shown (n = 6/group). C: control, L: LPS, L+Nil: LPS+Nilotinib, ***p < 0.001.

**Table S1.** One-way ANOVA (Tukey’s test) and significance of the results of the *in vitro* experiments in this study.

| **Figure 1B MTT** |
| --- |
| \| Number of families \| 1 \|  \|  \|  \|  \|  \| \| --- \| --- \| --- \| --- \| --- \| --- \| --- \| \| Number of comparisons per family \| 66 \|  \|  \|  \|  \|  \| \| Alpha \| 0.05 \|  \|  \|  \|  \|  \| \|  \|  \|  \|  \|  \|  \|  \| \| Tukey's multiple comparisons test \| Mean Diff. \| 95.00% CI of diff. \| Significant? \| Summary \| Adjusted P Value \|  \| \|  \|  \|  \|  \|  \|  \|  \| \| DMSO (0.001%) vs. Nilotinib 0.1 μM \| 4.064 \| -5.925 to 14.05 \| No \| ns \| 0.9708 \| A-B \| \| DMSO (0.001%) vs. DMSO (0.01%) \| 1.429e-006 \| -9.989 to 9.989 \| No \| ns \| >0.9999 \| A-C \| \| DMSO (0.001%) vs. Nilotinib 1 μM \| -1.05 \| -11.04 to 8.939 \| No \| ns \| >0.9999 \| A-D \| \| DMSO (0.001%) vs. DMSO (0.05%) \| 1.214e-005 \| -9.989 to 9.989 \| No \| ns \| >0.9999 \| A-E \| \| DMSO (0.001%) vs. Nilotinib 5 μM \| -0.5943 \| -10.58 to 9.395 \| No \| ns \| >0.9999 \| A-F \| \| DMSO (0.001%) vs. DMSO (0.1%) \| 1e-005 \| -9.989 to 9.989 \| No \| ns \| >0.9999 \| A-G \| \| DMSO (0.001%) vs. Nilotinib 10 μM \| -1.139 \| -11.13 to 8.85 \| No \| ns \| >0.9999 \| A-H \| \| DMSO (0.001%) vs. DMSO (0.25%) \| 7.143e-007 \| -9.989 to 9.989 \| No \| ns \| >0.9999 \| A-I \| \| DMSO (0.001%) vs. Nilotinib 25 μM \| 10.98 \| 0.9897 to 20.97 \| Yes \| * \| 0.0181 \| A-J \| \| DMSO (0.001%) vs. DMSO (0.5%) \| -4.286e-006 \| -9.989 to 9.989 \| No \| ns \| >0.9999 \| A-K \| \| DMSO (0.001%) vs. Nilotinib 50 μM \| 12.46 \| 2.469 to 22.45 \| Yes \| ** \| 0.0032 \| A-L \| \| Nilotinib 0.1 μM vs. DMSO (0.01%) \| -4.064 \| -14.05 to 5.925 \| No \| ns \| 0.9708 \| B-C \| \| Nilotinib 0.1 μM vs. Nilotinib 1 μM \| -5.114 \| -15.1 to 4.875 \| No \| ns \| 0.8662 \| B-D \| \| Nilotinib 0.1 μM vs. DMSO (0.05%) \| -4.064 \| -14.05 to 5.925 \| No \| ns \| 0.9708 \| B-E \| \| Nilotinib 0.1 μM vs. Nilotinib 5 μM \| -4.658 \| -14.65 to 5.331 \| No \| ns \| 0.9245 \| B-F \| \| Nilotinib 0.1 μM vs. DMSO (0.1%) \| -4.064 \| -14.05 to 5.925 \| No \| ns \| 0.9708 \| B-G \| \| Nilotinib 0.1 μM vs. Nilotinib 10 μM \| -5.203 \| -15.19 to 4.786 \| No \| ns \| 0.8523 \| B-H \| \| Nilotinib 0.1 μM vs. DMSO (0.25%) \| -4.064 \| -14.05 to 5.925 \| No \| ns \| 0.9708 \| B-I \| \| Nilotinib 0.1 μM vs. Nilotinib 25 μM \| 6.915 \| -3.074 to 16.9 \| No \| ns \| 0.4832 \| B-J \| \| Nilotinib 0.1 μM vs. DMSO (0.5%) \| -4.064 \| -14.05 to 5.925 \| No \| ns \| 0.9708 \| B-K \| \| Nilotinib 0.1 μM vs. Nilotinib 50 μM \| 8.394 \| -1.595 to 18.38 \| No \| ns \| 0.1954 \| B-L \| \| DMSO (0.01%) vs. Nilotinib 1 μM \| -1.05 \| -11.04 to 8.939 \| No \| ns \| >0.9999 \| C-D \| \| DMSO (0.01%) vs. DMSO (0.05%) \| 1.071e-005 \| -9.989 to 9.989 \| No \| ns \| >0.9999 \| C-E \| \| DMSO (0.01%) vs. Nilotinib 5 μM \| -0.5943 \| -10.58 to 9.395 \| No \| ns \| >0.9999 \| C-F \| \| DMSO (0.01%) vs. DMSO (0.1%) \| 8.571e-006 \| -9.989 to 9.989 \| No \| ns \| >0.9999 \| C-G \| \| DMSO (0.01%) vs. Nilotinib 10 μM \| -1.139 \| -11.13 to 8.85 \| No \| ns \| >0.9999 \| C-H \| \| DMSO (0.01%) vs. DMSO (0.25%) \| -7.143e-007 \| -9.989 to 9.989 \| No \| ns \| >0.9999 \| C-I \| \| DMSO (0.01%) vs. Nilotinib 25 μM \| 10.98 \| 0.9897 to 20.97 \| Yes \| * \| 0.0181 \| C-J \| \| DMSO (0.01%) vs. DMSO (0.5%) \| -5.714e-006 \| -9.989 to 9.989 \| No \| ns \| >0.9999 \| C-K \| \| DMSO (0.01%) vs. Nilotinib 50 μM \| 12.46 \| 2.469 to 22.45 \| Yes \| ** \| 0.0032 \| C-L \| \| Nilotinib 1 μM vs. DMSO (0.05%) \| 1.05 \| -8.939 to 11.04 \| No \| ns \| >0.9999 \| D-E \| \| Nilotinib 1 μM vs. Nilotinib 5 μM \| 0.4557 \| -9.533 to 10.44 \| No \| ns \| >0.9999 \| D-F \| \| Nilotinib 1 μM vs. DMSO (0.1%) \| 1.05 \| -8.939 to 11.04 \| No \| ns \| >0.9999 \| D-G \| \| Nilotinib 1 μM vs. Nilotinib 10 μM \| -0.08901 \| -10.08 to 9.9 \| No \| ns \| >0.9999 \| D-H \| \| Nilotinib 1 μM vs. DMSO (0.25%) \| 1.05 \| -8.939 to 11.04 \| No \| ns \| >0.9999 \| D-I \| \| Nilotinib 1 μM vs. Nilotinib 25 μM \| 12.03 \| 2.04 to 22.02 \| Yes \| ** \| 0.0054 \| D-J \| \| Nilotinib 1 μM vs. DMSO (0.5%) \| 1.05 \| -8.939 to 11.04 \| No \| ns \| >0.9999 \| D-K \| \| Nilotinib 1 μM vs. Nilotinib 50 μM \| 13.51 \| 3.519 to 23.5 \| Yes \| *** \| 0.0008 \| D-L \| \| DMSO (0.05%) vs. Nilotinib 5 μM \| -0.5943 \| -10.58 to 9.395 \| No \| ns \| >0.9999 \| E-F \| \| DMSO (0.05%) vs. DMSO (0.1%) \| -2.143e-006 \| -9.989 to 9.989 \| No \| ns \| >0.9999 \| E-G \| \| DMSO (0.05%) vs. Nilotinib 10 μM \| -1.139 \| -11.13 to 8.85 \| No \| ns \| >0.9999 \| E-H \| \| DMSO (0.05%) vs. DMSO (0.25%) \| -1.143e-005 \| -9.989 to 9.989 \| No \| ns \| >0.9999 \| E-I \| \| DMSO (0.05%) vs. Nilotinib 25 μM \| 10.98 \| 0.9897 to 20.97 \| Yes \| * \| 0.0181 \| E-J \| \| DMSO (0.05%) vs. DMSO (0.5%) \| -1.643e-005 \| -9.989 to 9.989 \| No \| ns \| >0.9999 \| E-K \| \| DMSO (0.05%) vs. Nilotinib 50 μM \| 12.46 \| 2.469 to 22.45 \| Yes \| ** \| 0.0032 \| E-L \| \| Nilotinib 5 μM vs. DMSO (0.1%) \| 0.5943 \| -9.395 to 10.58 \| No \| ns \| >0.9999 \| F-G \| \| Nilotinib 5 μM vs. Nilotinib 10 μM \| -0.5447 \| -10.53 to 9.444 \| No \| ns \| >0.9999 \| F-H \| \| Nilotinib 5 μM vs. DMSO (0.25%) \| 0.5943 \| -9.395 to 10.58 \| No \| ns \| >0.9999 \| F-I \| \| Nilotinib 5 μM vs. Nilotinib 25 μM \| 11.57 \| 1.584 to 21.56 \| Yes \| ** \| 0.0093 \| F-J \| \| Nilotinib 5 μM vs. DMSO (0.5%) \| 0.5943 \| -9.395 to 10.58 \| No \| ns \| >0.9999 \| F-K \| \| Nilotinib 5 μM vs. Nilotinib 50 μM \| 13.05 \| 3.063 to 23.04 \| Yes \| ** \| 0.0015 \| F-L \| \| DMSO (0.1%) vs. Nilotinib 10 μM \| -1.139 \| -11.13 to 8.85 \| No \| ns \| >0.9999 \| G-H \| \| DMSO (0.1%) vs. DMSO (0.25%) \| -9.286e-006 \| -9.989 to 9.989 \| No \| ns \| >0.9999 \| G-I \| \| DMSO (0.1%) vs. Nilotinib 25 μM \| 10.98 \| 0.9897 to 20.97 \| Yes \| * \| 0.0181 \| G-J \| \| DMSO (0.1%) vs. DMSO (0.5%) \| -1.429e-005 \| -9.989 to 9.989 \| No \| ns \| >0.9999 \| G-K \| \| DMSO (0.1%) vs. Nilotinib 50 μM \| 12.46 \| 2.469 to 22.45 \| Yes \| ** \| 0.0032 \| G-L \| \| Nilotinib 10 μM vs. DMSO (0.25%) \| 1.139 \| -8.85 to 11.13 \| No \| ns \| >0.9999 \| H-I \| \| Nilotinib 10 μM vs. Nilotinib 25 μM \| 12.12 \| 2.129 to 22.11 \| Yes \| ** \| 0.0049 \| H-J \| \| Nilotinib 10 μM vs. DMSO (0.5%) \| 1.139 \| -8.85 to 11.13 \| No \| ns \| >0.9999 \| H-K \| \| Nilotinib 10 μM vs. Nilotinib 50 μM \| 13.6 \| 3.608 to 23.59 \| Yes \| *** \| 0.0007 \| H-L \| \| DMSO (0.25%) vs. Nilotinib 25 μM \| 10.98 \| 0.9897 to 20.97 \| Yes \| * \| 0.0181 \| I-J \| \| DMSO (0.25%) vs. DMSO (0.5%) \| -5e-006 \| -9.989 to 9.989 \| No \| ns \| >0.9999 \| I-K \| \| DMSO (0.25%) vs. Nilotinib 50 μM \| 12.46 \| 2.469 to 22.45 \| Yes \| ** \| 0.0032 \| I-L \| \| Nilotinib 25 μM vs. DMSO (0.5%) \| -10.98 \| -20.97 to -0.9898 \| Yes \| * \| 0.0181 \| J-K \| \| Nilotinib 25 μM vs. Nilotinib 50 μM \| 1.479 \| -8.51 to 11.47 \| No \| ns \| >0.9999 \| J-L \| \| DMSO (0.5%) vs. Nilotinib 50 μM \| 12.46 \| 2.469 to 22.45 \| Yes \| ** \| 0.0032 \| K-L \| |
| **Figure 1d c-Abl** |
| \| Number of families \| 1 \|  \|  \|  \|  \|  \| \| --- \| --- \| --- \| --- \| --- \| --- \| --- \| \| Number of comparisons per family \| 3 \|  \|  \|  \|  \|  \| \| Alpha \| 0.05 \|  \|  \|  \|  \|  \| \|  \|  \|  \|  \|  \|  \|  \| \| Tukey's multiple comparisons test \| Mean Diff. \| 95.00% CI of diff. \| Significant? \| Summary \| Adjusted P Value \|  \| \|  \|  \|  \|  \|  \|  \|  \| \| C vs. L \| -19.46 \| -33.19 to -5.728 \| Yes \| ** \| 0.0027 \| A-B \| \| C vs. Nil+L \| -3.243 \| -18.24 to 11.76 \| No \| ns \| 0.8668 \| A-C \| \| L vs. Nil+L \| 16.21 \| 4.345 to 28.08 \| Yes \| ** \| 0.0041 \| B-C \| |
| **Figure 1f c-Abl** |
| \| Number of families \| 1 \|  \|  \|  \|  \|  \| \| --- \| --- \| --- \| --- \| --- \| --- \| --- \| \| Number of comparisons per family \| 3 \|  \|  \|  \|  \|  \| \| Alpha \| 0.05 \|  \|  \|  \|  \|  \| \|  \|  \|  \|  \|  \|  \|  \| \| Tukey's multiple comparisons test \| Mean Diff. \| 95.00% CI of diff. \| Significant? \| Summary \| Adjusted P Value \|  \| \|  \|  \|  \|  \|  \|  \|  \| \| C vs. L \| -67.96 \| -85.3 to -50.61 \| Yes \| **** \| <0.0001 \| A-B \| \| C vs. L+Nil \| 11.03 \| -8 to 30.06 \| No \| ns \| 0.3611 \| A-C \| \| L vs. L+Nil \| 78.99 \| 59.16 to 98.82 \| Yes \| **** \| <0.0001 \| B-C \| |
| **Figure 1g COX-2** |
| \| Number of families \| 1 \|  \|  \|  \|  \|  \| \| --- \| --- \| --- \| --- \| --- \| --- \| --- \| \| Number of comparisons per family \| 3 \|  \|  \|  \|  \|  \| \| Alpha \| 0.05 \|  \|  \|  \|  \|  \| \|  \|  \|  \|  \|  \|  \|  \| \| Tukey's multiple comparisons test \| Mean Diff. \| 95.00% CI of diff. \| Significant? \| Summary \| Adjusted P Value \|  \| \|  \|  \|  \|  \|  \|  \|  \| \| C vs. L \| -14.28 \| -19.33 to -9.221 \| Yes \| **** \| <0.0001 \| A-B \| \| C vs. Nil+L \| -0.4273 \| -5.482 to 4.627 \| No \| ns \| 0.9753 \| A-C \| \| L vs. Nil+L \| 13.85 \| 8.794 to 18.9 \| Yes \| **** \| <0.0001 \| B-C \| |
| **Figure 1g IL-1β** |
| \| Number of families \| 1 \|  \|  \|  \|  \|  \| \| --- \| --- \| --- \| --- \| --- \| --- \| --- \| \| Number of comparisons per family \| 3 \|  \|  \|  \|  \|  \| \| Alpha \| 0.05 \|  \|  \|  \|  \|  \| \|  \|  \|  \|  \|  \|  \|  \| \| Tukey's multiple comparisons test \| Mean Diff. \| 95.00% CI of diff. \| Significant? \| Summary \| Adjusted P Value \|  \| \|  \|  \|  \|  \|  \|  \|  \| \| C vs. L \| -66.94 \| -77.23 to -56.64 \| Yes \| **** \| <0.0001 \| A-B \| \| C vs. Nil+L \| -0.7857 \| -11.08 to 9.508 \| No \| ns \| 0.9798 \| A-C \| \| L vs. Nil+L \| 66.15 \| 55.86 to 76.44 \| Yes \| **** \| <0.0001 \| B-C \| |
| **Figure 1g IL-6** |
| \| Number of families \| 1 \|  \|  \|  \|  \|  \| \| --- \| --- \| --- \| --- \| --- \| --- \| --- \| \| Number of comparisons per family \| 3 \|  \|  \|  \|  \|  \| \| Alpha \| 0.05 \|  \|  \|  \|  \|  \| \|  \|  \|  \|  \|  \|  \|  \| \| Tukey's multiple comparisons test \| Mean Diff. \| 95.00% CI of diff. \| Significant? \| Summary \| Adjusted P Value \|  \| \|  \|  \|  \|  \|  \|  \|  \| \| C vs. L \| -23.91 \| -30.19 to -17.62 \| Yes \| **** \| <0.0001 \| A-B \| \| C vs. Nil+L \| -10.13 \| -16.41 to -3.846 \| Yes \| ** \| 0.0016 \| A-C \| \| L vs. Nil+L \| 13.78 \| 7.497 to 20.06 \| Yes \| **** \| <0.0001 \| B-C \| |
| **Figure 1g iNOS** |
| \| Number of families \| 1 \|  \|  \|  \|  \|  \| \| --- \| --- \| --- \| --- \| --- \| --- \| --- \| \| Number of comparisons per family \| 3 \|  \|  \|  \|  \|  \| \| Alpha \| 0.05 \|  \|  \|  \|  \|  \| \|  \|  \|  \|  \|  \|  \|  \| \| Tukey's multiple comparisons test \| Mean Diff. \| 95.00% CI of diff. \| Significant? \| Summary \| Adjusted P Value \|  \| \|  \|  \|  \|  \|  \|  \|  \| \| C vs. L \| -13.86 \| -24.6 to -3.116 \| Yes \| * \| 0.0102 \| A-B \| \| C vs. Nil+L \| -20.14 \| -30.88 to -9.4 \| Yes \| *** \| 0.0003 \| A-C \| \| L vs. Nil+L \| -6.284 \| -17.03 to 4.457 \| No \| ns \| 0.3229 \| B-C \| |
| **Figure 1h COX-2** |
| \| Number of families \| 1 \|  \|  \|  \|  \|  \| \| --- \| --- \| --- \| --- \| --- \| --- \| --- \| \| Number of comparisons per family \| 3 \|  \|  \|  \|  \|  \| \| Alpha \| 0.05 \|  \|  \|  \|  \|  \| \|  \|  \|  \|  \|  \|  \|  \| \| Tukey's multiple comparisons test \| Mean Diff. \| 95.00% CI of diff. \| Significant? \| Summary \| Adjusted P Value \|  \| \|  \|  \|  \|  \|  \|  \|  \| \| C vs. L \| -39.61 \| -48.46 to -30.75 \| Yes \| **** \| <0.0001 \| A-B \| \| C vs. L+Nil \| -4.746 \| -13.6 to 4.108 \| No \| ns \| 0.3695 \| A-C \| \| L vs. L+Nil \| 34.86 \| 26.01 to 43.72 \| Yes \| **** \| <0.0001 \| B-C \| |
| **Figure 1h IL-1β** |
| \| Number of families \| 1 \|  \|  \|  \|  \|  \| \| --- \| --- \| --- \| --- \| --- \| --- \| --- \| \| Number of comparisons per family \| 3 \|  \|  \|  \|  \|  \| \| Alpha \| 0.05 \|  \|  \|  \|  \|  \| \|  \|  \|  \|  \|  \|  \|  \| \| Tukey's multiple comparisons test \| Mean Diff. \| 95.00% CI of diff. \| Significant? \| Summary \| Adjusted P Value \|  \| \|  \|  \|  \|  \|  \|  \|  \| \| C vs. L \| -587 \| -659.1 to -514.9 \| Yes \| **** \| <0.0001 \| A-B \| \| C vs. L+Nil \| -19.54 \| -91.63 to 52.55 \| No \| ns \| 0.7648 \| A-C \| \| L vs. L+Nil \| 567.5 \| 495.4 to 639.5 \| Yes \| **** \| <0.0001 \| B-C \| |
| **Figure 1h IL-6** |
| \| Number of families \| 1 \|  \|  \|  \|  \|  \| \| --- \| --- \| --- \| --- \| --- \| --- \| --- \| \| Number of comparisons per family \| 3 \|  \|  \|  \|  \|  \| \| Alpha \| 0.05 \|  \|  \|  \|  \|  \| \|  \|  \|  \|  \|  \|  \|  \| \| Tukey's multiple comparisons test \| Mean Diff. \| 95.00% CI of diff. \| Significant? \| Summary \| Adjusted P Value \|  \| \|  \|  \|  \|  \|  \|  \|  \| \| C vs. L \| -422.2 \| -510 to -334.4 \| Yes \| **** \| <0.0001 \| A-B \| \| C vs. L+Nil \| -88.83 \| -176.6 to -1.042 \| Yes \| * \| 0.0472 \| A-C \| \| L vs. L+Nil \| 333.4 \| 245.6 to 421.2 \| Yes \| **** \| <0.0001 \| B-C \| |
| **Figure 1h iNOS** |
| \| Number of families \| 1 \|  \|  \|  \|  \|  \| \| --- \| --- \| --- \| --- \| --- \| --- \| --- \| \| Number of comparisons per family \| 3 \|  \|  \|  \|  \|  \| \| Alpha \| 0.05 \|  \|  \|  \|  \|  \| \|  \|  \|  \|  \|  \|  \|  \| \| Tukey's multiple comparisons test \| Mean Diff. \| 95.00% CI of diff. \| Significant? \| Summary \| Adjusted P Value \|  \| \|  \|  \|  \|  \|  \|  \|  \| \| C vs. L \| -62.18 \| -97.97 to -26.38 \| Yes \| ** \| 0.0011 \| A-B \| \| C vs. L+Nil \| -83.88 \| -119.7 to -48.08 \| Yes \| **** \| <0.0001 \| A-C \| \| L vs. L+Nil \| -21.7 \| -57.49 to 14.1 \| No \| ns \| 0.2865 \| B-C \| |
| **Figure 1i IL-4 6h** |
| \| Number of families \| 1 \|  \|  \|  \|  \|  \| \| --- \| --- \| --- \| --- \| --- \| --- \| --- \| \| Number of comparisons per family \| 3 \|  \|  \|  \|  \|  \| \| Alpha \| 0.05 \|  \|  \|  \|  \|  \| \|  \|  \|  \|  \|  \|  \|  \| \| Tukey's multiple comparisons test \| Mean Diff. \| 95.00% CI of diff. \| Significant? \| Summary \| Adjusted P Value \|  \| \|  \|  \|  \|  \|  \|  \|  \| \| C vs. L \| 0.2925 \| -0.8424 to 1.427 \| No \| ns \| 0.7787 \| A-B \| \| C vs. L+Nil \| 0.02961 \| -1.057 to 1.116 \| No \| ns \| 0.9971 \| A-C \| \| L vs. L+Nil \| -0.2629 \| -1.349 to 0.8237 \| No \| ns \| 0.8018 \| B-C \| |
| **Figure 1i IL-4 12h** |
| \| Number of families \| 1 \|  \|  \|  \|  \|  \| \| --- \| --- \| --- \| --- \| --- \| --- \| --- \| \| Number of comparisons per family \| 3 \|  \|  \|  \|  \|  \| \| Alpha \| 0.05 \|  \|  \|  \|  \|  \| \|  \|  \|  \|  \|  \|  \|  \| \| Tukey's multiple comparisons test \| Mean Diff. \| 95.00% CI of diff. \| Significant? \| Summary \| Adjusted P Value \|  \| \|  \|  \|  \|  \|  \|  \|  \| \| C vs. L \| 0.4242 \| -0.2953 to 1.144 \| No \| ns \| 0.3176 \| D-E \| \| C vs. L+Nil \| -1.22 \| -1.94 to -0.5004 \| Yes \| *** \| 0.0009 \| D-F \| \| L vs. L+Nil \| -1.644 \| -2.364 to -0.9246 \| Yes \| **** \| <0.0001 \| E-F \| |
| **Figure 1i IL-4 24h** |
| \| Number of families \| 1 \|  \|  \|  \|  \|  \| \| --- \| --- \| --- \| --- \| --- \| --- \| --- \| \| Number of comparisons per family \| 3 \|  \|  \|  \|  \|  \| \| Alpha \| 0.05 \|  \|  \|  \|  \|  \| \|  \|  \|  \|  \|  \|  \|  \| \| Tukey's multiple comparisons test \| Mean Diff. \| 95.00% CI of diff. \| Significant? \| Summary \| Adjusted P Value \|  \| \|  \|  \|  \|  \|  \|  \|  \| \| C vs. L \| -0.1691 \| -1.569 to 1.231 \| No \| ns \| 0.9503 \| G-H \| \| C vs. L+Nil \| -2.771 \| -4.171 to -1.371 \| Yes \| *** \| 0.0002 \| G-I \| \| L vs. L+Nil \| -2.602 \| -4.002 to -1.202 \| Yes \| *** \| 0.0004 \| H-I \| |
| **Figure 1i IL-10 6h** |
| \| Number of families \| 1 \|  \|  \|  \|  \|  \| \| --- \| --- \| --- \| --- \| --- \| --- \| --- \| \| Number of comparisons per family \| 3 \|  \|  \|  \|  \|  \| \| Alpha \| 0.05 \|  \|  \|  \|  \|  \| \|  \|  \|  \|  \|  \|  \|  \| \| Tukey's multiple comparisons test \| Mean Diff. \| 95.00% CI of diff. \| Significant? \| Summary \| Adjusted P Value \|  \| \|  \|  \|  \|  \|  \|  \|  \| \| C vs. L \| 0.06399 \| -1.558 to 1.686 \| No \| ns \| 0.9940 \| A-B \| \| C vs. L+Nil \| -0.4402 \| -1.993 to 1.112 \| No \| ns \| 0.7397 \| A-C \| \| L vs. L+Nil \| -0.5042 \| -2.057 to 1.048 \| No \| ns \| 0.6753 \| B-C \| |
| **Figure 1i IL-10 12h** |
| \| Number of families \| 1 \|  \|  \|  \|  \|  \| \| --- \| --- \| --- \| --- \| --- \| --- \| --- \| \| Number of comparisons per family \| 3 \|  \|  \|  \|  \|  \| \| Alpha \| 0.05 \|  \|  \|  \|  \|  \| \|  \|  \|  \|  \|  \|  \|  \| \| Tukey's multiple comparisons test \| Mean Diff. \| 95.00% CI of diff. \| Significant? \| Summary \| Adjusted P Value \|  \| \|  \|  \|  \|  \|  \|  \|  \| \| C vs. L \| 0.134 \| -0.591 to 0.859 \| No \| ns \| 0.8879 \| D-E \| \| C vs. L+Nil \| -0.7063 \| -1.431 to 0.01867 \| No \| ns \| 0.0571 \| D-F \| \| L vs. L+Nil \| -0.8403 \| -1.565 to -0.1153 \| Yes \| * \| 0.0213 \| E-F \| |
| **Figure 1i IL-10 24h** |
| \| Number of families \| 1 \|  \|  \|  \|  \|  \| \| --- \| --- \| --- \| --- \| --- \| --- \| --- \| \| Number of comparisons per family \| 3 \|  \|  \|  \|  \|  \| \| Alpha \| 0.05 \|  \|  \|  \|  \|  \| \|  \|  \|  \|  \|  \|  \|  \| \| Tukey's multiple comparisons test \| Mean Diff. \| 95.00% CI of diff. \| Significant? \| Summary \| Adjusted P Value \|  \| \|  \|  \|  \|  \|  \|  \|  \| \| C vs. L \| 0.107 \| -1.573 to 1.787 \| No \| ns \| 0.9859 \| G-H \| \| C vs. L+Nil \| -2.849 \| -4.529 to -1.17 \| Yes \| *** \| 0.0009 \| G-I \| \| L vs. L+Nil \| -2.956 \| -4.636 to -1.277 \| Yes \| *** \| 0.0006 \| H-I \| |
| **Figure 2b IL-1β** |
| \| Number of families \| 1 \|  \|  \|  \|  \|  \| \| --- \| --- \| --- \| --- \| --- \| --- \| --- \| \| Number of comparisons per family \| 10 \|  \|  \|  \|  \|  \| \| Alpha \| 0.05 \|  \|  \|  \|  \|  \| \|  \|  \|  \|  \|  \|  \|  \| \| Tukey's multiple comparisons test \| Mean Diff. \| 95.00% CI of diff. \| Significant? \| Summary \|  \|  \| \|  \|  \|  \|  \|  \|  \|  \| \| C vs. L \| -44 \| -77.81 to -10.19 \| Yes \| ** \|  \| A-B \| \| C vs. L+Nil \| -2.999 \| -36.81 to 30.81 \| No \| ns \|  \| A-C \| \| C vs. L+TAK \| -25.4 \| -59.21 to 8.415 \| No \| ns \|  \| A-D \| \| C vs. L+TAK+Nil \| 12.13 \| -21.69 to 45.94 \| No \| ns \|  \| A-E \| \| L vs. L+Nil \| 41 \| 7.188 to 74.81 \| Yes \| * \|  \| B-C \| \| L vs. L+TAK \| 18.6 \| -15.21 to 52.42 \| No \| ns \|  \| B-D \| \| L vs. L+TAK+Nil \| 56.13 \| 22.31 to 89.94 \| Yes \| *** \|  \| B-E \| \| L+Nil vs. L+TAK \| -22.4 \| -56.21 to 11.41 \| No \| ns \|  \| C-D \| \| L+Nil vs. L+TAK+Nil \| 15.13 \| -18.69 to 48.94 \| No \| ns \|  \| C-E \| \| L+TAK vs. L+TAK+Nil \| 37.52 \| 3.711 to 71.34 \| Yes \| * \|  \| D-E \| |
| **Figure 2c GAPDH** |
| \| Number of families \| 1 \|  \|  \|  \|  \|  \| \| --- \| --- \| --- \| --- \| --- \| --- \| --- \| \| Number of comparisons per family \| 10 \|  \|  \|  \|  \|  \| \| Alpha \| 0.05 \|  \|  \|  \|  \|  \| \|  \|  \|  \|  \|  \|  \|  \| \| Tukey's multiple comparisons test \| Mean Diff. \| 95.00% CI of diff. \| Significant? \| Summary \|  \|  \| \|  \|  \|  \|  \|  \|  \|  \| \| C vs. L \| -1.725 \| -31.38 to 27.93 \| No \| ns \|  \| A-B \| \| C vs. L+Nil \| -0.4914 \| -30.15 to 29.17 \| No \| ns \|  \| A-C \| \| C vs. L+TAK \| -1.128 \| -30.79 to 28.53 \| No \| ns \|  \| A-D \| \| C vs. L+TAK+Nil \| 1.698 \| -27.96 to 31.36 \| No \| ns \|  \| A-E \| \| L vs. L+Nil \| 1.234 \| -28.43 to 30.89 \| No \| ns \|  \| B-C \| \| L vs. L+TAK \| 0.5976 \| -29.06 to 30.26 \| No \| ns \|  \| B-D \| \| L vs. L+TAK+Nil \| 3.423 \| -26.24 to 33.08 \| No \| ns \|  \| B-E \| \| L+Nil vs. L+TAK \| -0.6363 \| -30.3 to 29.02 \| No \| ns \|  \| C-D \| \| L+Nil vs. L+TAK+Nil \| 2.189 \| -27.47 to 31.85 \| No \| ns \|  \| C-E \| \| L+TAK vs. L+TAK+Nil \| 2.825 \| -26.83 to 32.48 \| No \| ns \|  \| D-E \| |
| **Figure 2d p-AKT** |
| \| Number of families \| 1 \|  \|  \|  \|  \|  \| \| --- \| --- \| --- \| --- \| --- \| --- \| --- \| \| Number of comparisons per family \| 3 \|  \|  \|  \|  \|  \| \| Alpha \| 0.05 \|  \|  \|  \|  \|  \| \|  \|  \|  \|  \|  \|  \|  \| \| Tukey's multiple comparisons test \| Mean Diff. \| 95.00% CI of diff. \| Significant? \| Summary \| Adjusted P Value \|  \| \|  \|  \|  \|  \|  \|  \|  \| \| C vs. L \| -80.04 \| -130.3 to -29.83 \| Yes \| ** \| 0.0030 \| A-B \| \| C vs. L+Nil \| 44.87 \| -5.339 to 95.08 \| No \| ns \| 0.0818 \| A-C \| \| L vs. L+Nil \| 124.9 \| 74.7 to 175.1 \| Yes \| **** \| <0.0001 \| B-C \| |
| **Figure 2d AKT** |
| \| Number of families \| 1 \|  \|  \|  \|  \|  \| \| --- \| --- \| --- \| --- \| --- \| --- \| --- \| \| Number of comparisons per family \| 3 \|  \|  \|  \|  \|  \| \| Alpha \| 0.05 \|  \|  \|  \|  \|  \| \|  \|  \|  \|  \|  \|  \|  \| \| Tukey's multiple comparisons test \| Mean Diff. \| 95.00% CI of diff. \| Significant? \| Summary \| Adjusted P Value \|  \| \|  \|  \|  \|  \|  \|  \|  \| \| C vs. L \| 10.53 \| -21.27 to 42.34 \| No \| ns \| 0.6604 \| A-B \| \| C vs. L+Nil \| -1.39 \| -33.19 to 30.42 \| No \| ns \| 0.9925 \| A-C \| \| L vs. L+Nil \| -11.92 \| -43.73 to 19.88 \| No \| ns \| 0.5907 \| B-C \| |
| **Figure 2e p-P38** |
| \| Number of families \| 1 \|  \|  \|  \|  \|  \| \| --- \| --- \| --- \| --- \| --- \| --- \| --- \| \| Number of comparisons per family \| 3 \|  \|  \|  \|  \|  \| \| Alpha \| 0.05 \|  \|  \|  \|  \|  \| \|  \|  \|  \|  \|  \|  \|  \| \| Tukey's multiple comparisons test \| Mean Diff. \| 95.00% CI of diff. \| Significant? \| Summary \| Adjusted P Value \|  \| \|  \|  \|  \|  \|  \|  \|  \| \| C vs. L \| -586.8 \| -851.3 to -322.4 \| Yes \| *** \| 0.0002 \| A-B \| \| C vs. L+Nil \| -58.81 \| -323.2 to 205.6 \| No \| ns \| 0.8262 \| A-C \| \| L vs. L+Nil \| 528 \| 263.6 to 792.4 \| Yes \| *** \| 0.0005 \| B-C \| |
| **Figure 2e P38** |
| \| Number of families \| 1 \|  \|  \|  \|  \|  \| \| --- \| --- \| --- \| --- \| --- \| --- \| --- \| \| Number of comparisons per family \| 3 \|  \|  \|  \|  \|  \| \| Alpha \| 0.05 \|  \|  \|  \|  \|  \| \|  \|  \|  \|  \|  \|  \|  \| \| Tukey's multiple comparisons test \| Mean Diff. \| 95.00% CI of diff. \| Significant? \| Summary \| Adjusted P Value \|  \| \|  \|  \|  \|  \|  \|  \|  \| \| C vs. L \| 10.75 \| -11.24 to 32.75 \| No \| ns \| 0.4196 \| A-B \| \| C vs. L+Nil \| 1.856 \| -20.14 to 23.85 \| No \| ns \| 0.9725 \| A-C \| \| L vs. L+Nil \| -8.896 \| -30.89 to 13.1 \| No \| ns \| 0.5442 \| B-C \| |
| **Figure 2g p-AKT** |
| \| Number of families \| 1 \|  \|  \|  \|  \|  \| \| --- \| --- \| --- \| --- \| --- \| --- \| --- \| \| Number of comparisons per family \| 3 \|  \|  \|  \|  \|  \| \| Alpha \| 0.05 \|  \|  \|  \|  \|  \| \|  \|  \|  \|  \|  \|  \|  \| \| Tukey's multiple comparisons test \| Mean Diff. \| 95.00% CI of diff. \| Significant? \| Summary \| Adjusted P Value \|  \| \|  \|  \|  \|  \|  \|  \|  \| \| C vs. L \| -63.81 \| -85.41 to -42.21 \| Yes \| **** \| <0.0001 \| A-B \| \| C vs. L+Nil \| 19.42 \| -4.649 to 43.5 \| No \| ns \| 0.1406 \| A-C \| \| L vs. L+Nil \| 83.24 \| 58.63 to 107.8 \| Yes \| **** \| <0.0001 \| B-C \| |
| **Figure 2i p-P38** |
| \| Number of families \| 1 \|  \|  \|  \|  \|  \| \| --- \| --- \| --- \| --- \| --- \| --- \| --- \| \| Number of comparisons per family \| 3 \|  \|  \|  \|  \|  \| \| Alpha \| 0.05 \|  \|  \|  \|  \|  \| \|  \|  \|  \|  \|  \|  \|  \| \| Tukey's multiple comparisons test \| Mean Diff. \| 95.00% CI of diff. \| Significant? \| Summary \| Adjusted P Value \|  \| \|  \|  \|  \|  \|  \|  \|  \| \| C vs. L \| -114.6 \| -128.7 to -100.5 \| Yes \| **** \| <0.0001 \| A-B \| \| C vs. L+Nil \| 6.158 \| -9.65 to 21.97 \| No \| ns \| 0.6309 \| A-C \| \| L vs. L+Nil \| 120.8 \| 105.7 to 135.8 \| Yes \| **** \| <0.0001 \| B-C \| |
| **Figure 2j p-STAT3^s727^** |
| \| Number of families \| 1 \|  \|  \|  \|  \|  \| \| --- \| --- \| --- \| --- \| --- \| --- \| --- \| \| Number of comparisons per family \| 3 \|  \|  \|  \|  \|  \| \| Alpha \| 0.05 \|  \|  \|  \|  \|  \| \|  \|  \|  \|  \|  \|  \|  \| \| Tukey's multiple comparisons test \| Mean Diff. \| 95.00% CI of diff. \| Significant? \| Summary \| Adjusted P Value \|  \| \|  \|  \|  \|  \|  \|  \|  \| \| C vs. L \| -45.23 \| -90.35 to -0.1214 \| Yes \| * \| 0.0494 \| A-B \| \| C vs. L+Nil \| 7.279 \| -37.83 to 52.39 \| No \| ns \| 0.8954 \| A-C \| \| L vs. L+Nil \| 52.51 \| 7.4 to 97.63 \| Yes \| * \| 0.0245 \| B-C \| |
| **Figure 2k p-STAT3^s727^** |
| \| Number of families \| 1 \|  \|  \|  \|  \|  \| \| --- \| --- \| --- \| --- \| --- \| --- \| --- \| \| Number of comparisons per family \| 3 \|  \|  \|  \|  \|  \| \| Alpha \| 0.05 \|  \|  \|  \|  \|  \| \|  \|  \|  \|  \|  \|  \|  \| \| Tukey's multiple comparisons test \| Mean Diff. \| 95.00% CI of diff. \| Significant? \| Summary \| Adjusted P Value \|  \| \|  \|  \|  \|  \|  \|  \|  \| \| C vs. L \| -23.3 \| -40.33 to -6.27 \| Yes \| ** \| 0.0040 \| A-B \| \| C vs. L+Nil \| 0.1194 \| -19.22 to 19.46 \| No \| ns \| 0.9999 \| A-C \| \| L vs. L+Nil \| 23.42 \| 5.549 to 41.29 \| Yes \| ** \| 0.0062 \| B-C \| |
| **Figure 3a SOD2** |
| \| Number of families \| 1 \|  \|  \|  \|  \|  \| \| --- \| --- \| --- \| --- \| --- \| --- \| --- \| \| Number of comparisons per family \| 3 \|  \|  \|  \|  \|  \| \| Alpha \| 0.05 \|  \|  \|  \|  \|  \| \|  \|  \|  \|  \|  \|  \|  \| \| Tukey's multiple comparisons test \| Mean Diff. \| 95.00% CI of diff. \| Significant? \| Summary \| Adjusted P Value \|  \| \|  \|  \|  \|  \|  \|  \|  \| \| C vs. L \| -7.083 \| -10.23 to -3.936 \| Yes \| **** \| <0.0001 \| A-B \| \| C vs. L+Nil \| -3.634 \| -6.781 to -0.4877 \| Yes \| * \| 0.0230 \| A-C \| \| L vs. L+Nil \| 3.448 \| 0.302 to 6.595 \| Yes \| * \| 0.0310 \| B-C \| |
| **Figure 3b SOD2-scrambled** |
| \| Number of families \| 1 \|  \|  \|  \|  \|  \| \| --- \| --- \| --- \| --- \| --- \| --- \| --- \| \| Number of comparisons per family \| 3 \|  \|  \|  \|  \|  \| \| Alpha \| 0.05 \|  \|  \|  \|  \|  \| \|  \|  \|  \|  \|  \|  \|  \| \| Tukey's multiple comparisons test \| Mean Diff. \| 95.00% CI of diff. \| Significant? \| Summary \| Adjusted P Value \|  \| \|  \|  \|  \|  \|  \|  \|  \| \| C vs. L \| -1.11 \| -1.621 to -0.5993 \| Yes \| **** \| <0.0001 \| A-B \| \| C vs. L+Nil \| -0.4441 \| -1.026 to 0.1382 \| No \| ns \| 0.1546 \| A-C \| \| L vs. L+Nil \| 0.666 \| 0.0837 to 1.248 \| Yes \| * \| 0.0237 \| B-C \| |
| **Figure 3b SOD2-SOD2 siRNA** |
| \| Number of families \| 1 \|  \|  \|  \|  \|  \| \| --- \| --- \| --- \| --- \| --- \| --- \| --- \| \| Number of comparisons per family \| 3 \|  \|  \|  \|  \|  \| \| Alpha \| 0.05 \|  \|  \|  \|  \|  \| \|  \|  \|  \|  \|  \|  \|  \| \| Tukey's multiple comparisons test \| Mean Diff. \| 95.00% CI of diff. \| Significant? \| Summary \| Adjusted P Value \|  \| \|  \|  \|  \|  \|  \|  \|  \| \| C vs. L \| -0.07855 \| -0.126 to -0.03111 \| Yes \| ** \| 0.0014 \| E-F \| \| C vs. L+Nil \| -0.1222 \| -0.1763 to -0.06812 \| Yes \| **** \| <0.0001 \| E-G \| \| L vs. L+Nil \| -0.04366 \| -0.09775 to 0.01044 \| No \| ns \| 0.1268 \| F-G \| |
| **Figure 3c IL-6-scrambled** |
| \| Number of families \| 1 \|  \|  \|  \|  \|  \| \| --- \| --- \| --- \| --- \| --- \| --- \| --- \| \| Number of comparisons per family \| 3 \|  \|  \|  \|  \|  \| \| Alpha \| 0.05 \|  \|  \|  \|  \|  \| \|  \|  \|  \|  \|  \|  \|  \| \| Tukey's multiple comparisons test \| Mean Diff. \| 95.00% CI of diff. \| Significant? \| Summary \| Adjusted P Value \|  \| \|  \|  \|  \|  \|  \|  \|  \| \| C vs. L \| -5.31 \| -8.081 to -2.54 \| Yes \| *** \| 0.0003 \| A-B \| \| C vs. L+Nil \| -2.205 \| -5.364 to 0.9535 \| No \| ns \| 0.2038 \| A-C \| \| L vs. L+Nil \| 3.105 \| -0.05316 to 6.264 \| No \| ns \| 0.0544 \| B-C \| |
| **Figure 3d IL-6-SOD2 siRNA** |
| \| Number of families \| 1 \|  \|  \|  \|  \|  \| \| --- \| --- \| --- \| --- \| --- \| --- \| --- \| \| Number of comparisons per family \| 3 \|  \|  \|  \|  \|  \| \| Alpha \| 0.05 \|  \|  \|  \|  \|  \| \|  \|  \|  \|  \|  \|  \|  \| \| Tukey's multiple comparisons test \| Mean Diff. \| 95.00% CI of diff. \| Significant? \| Summary \| Adjusted P Value \|  \| \|  \|  \|  \|  \|  \|  \|  \| \| C vs. L \| -6.654 \| -9.117 to -4.191 \| Yes \| **** \| <0.0001 \| E-F \| \| C vs. L+Nil \| -6.01 \| -8.818 to -3.202 \| Yes \| **** \| <0.0001 \| E-G \| \| L vs. L+Nil \| 0.644 \| -2.164 to 3.452 \| No \| ns \| 0.8296 \| F-G \| |
| **Figure 3d IL-1β -scrambled** |
| \| Number of families \| 1 \|  \|  \|  \|  \|  \| \| --- \| --- \| --- \| --- \| --- \| --- \| --- \| \| Number of comparisons per family \| 3 \|  \|  \|  \|  \|  \| \| Alpha \| 0.05 \|  \|  \|  \|  \|  \| \|  \|  \|  \|  \|  \|  \|  \| \| Tukey's multiple comparisons test \| Mean Diff. \| 95.00% CI of diff. \| Significant? \| Summary \| Adjusted P Value \|  \| \|  \|  \|  \|  \|  \|  \|  \| \| C vs. L \| -47.88 \| -54.96 to -40.79 \| Yes \| **** \| <0.0001 \| A-B \| \| C vs. L+Nil \| -1.566 \| -9.218 to 6.086 \| No \| ns \| 0.8626 \| A-C \| \| L vs. L+Nil \| 46.31 \| 38.66 to 53.96 \| Yes \| **** \| <0.0001 \| B-C \| |
| **Figure 3e IL-1β-SOD2 siRNA** |
| \| Number of families \| 1 \|  \|  \|  \|  \|  \| \| --- \| --- \| --- \| --- \| --- \| --- \| --- \| \| Number of comparisons per family \| 3 \|  \|  \|  \|  \|  \| \| Alpha \| 0.05 \|  \|  \|  \|  \|  \| \|  \|  \|  \|  \|  \|  \|  \| \| Tukey's multiple comparisons test \| Mean Diff. \| 95.00% CI of diff. \| Significant? \| Summary \| Adjusted P Value \|  \| \|  \|  \|  \|  \|  \|  \|  \| \| C vs. L \| -138.8 \| -154.5 to -123 \| Yes \| **** \| <0.0001 \| E-F \| \| C vs. L+Nil \| -15.51 \| -32.49 to 1.47 \| No \| ns \| 0.0771 \| E-G \| \| L vs. L+Nil \| 123.3 \| 106.3 to 140.2 \| Yes \| **** \| <0.0001 \| F-G \| |
| **Figure 3e COX-2-scrambled** |
| \| Number of families \| 1 \|  \|  \|  \|  \|  \| \| --- \| --- \| --- \| --- \| --- \| --- \| --- \| \| Number of comparisons per family \| 3 \|  \|  \|  \|  \|  \| \| Alpha \| 0.05 \|  \|  \|  \|  \|  \| \|  \|  \|  \|  \|  \|  \|  \| \| Tukey's multiple comparisons test \| Mean Diff. \| 95.00% CI of diff. \| Significant? \| Summary \| Adjusted P Value \|  \| \|  \|  \|  \|  \|  \|  \|  \| \| C vs. L \| -3.793 \| -4.509 to -3.076 \| Yes \| **** \| <0.0001 \| A-B \| \| C vs. L+Nil \| 0.4351 \| -0.3389 to 1.209 \| No \| ns \| 0.3470 \| A-C \| \| L vs. L+Nil \| 4.228 \| 3.454 to 5.002 \| Yes \| **** \| <0.0001 \| B-C \| |
| **Figure 3e COX-2-SOD2 siRNA** |
| \| Number of families \| 1 \|  \|  \|  \|  \|  \| \| --- \| --- \| --- \| --- \| --- \| --- \| --- \| \| Number of comparisons per family \| 3 \|  \|  \|  \|  \|  \| \| Alpha \| 0.05 \|  \|  \|  \|  \|  \| \|  \|  \|  \|  \|  \|  \|  \| \| Tukey's multiple comparisons test \| Mean Diff. \| 95.00% CI of diff. \| Significant? \| Summary \| Adjusted P Value \|  \| \|  \|  \|  \|  \|  \|  \|  \| \| C vs. L \| -3.47 \| -4.885 to -2.055 \| Yes \| **** \| <0.0001 \| E-F \| \| C vs. L+Nil \| -0.4099 \| -1.938 to 1.118 \| No \| ns \| 0.7770 \| E-G \| \| L vs. L+Nil \| 3.06 \| 1.532 to 4.589 \| Yes \| *** \| 0.0002 \| F-G \| |
| **Figure 4b c-Abl** |
| \| Number of families \| 1 \|  \|  \|  \|  \|  \| \| --- \| --- \| --- \| --- \| --- \| --- \| --- \| \| Number of comparisons per family \| 3 \|  \|  \|  \|  \|  \| \| Alpha \| 0.05 \|  \|  \|  \|  \|  \| \|  \|  \|  \|  \|  \|  \|  \| \| Tukey's multiple comparisons test \| Mean Diff. \| 95.00% CI of diff. \| Significant? \| Summary \| Adjusted P Value \|  \| \|  \|  \|  \|  \|  \|  \|  \| \| C vs. L \| -129.9 \| -145.2 to -114.7 \| Yes \| **** \| <0.0001 \| A-B \| \| C vs. L+Nil \| -73.03 \| -91.47 to -54.59 \| Yes \| **** \| <0.0001 \| A-C \| \| L vs. L+Nil \| 56.88 \| 38.53 to 75.23 \| Yes \| **** \| <0.0001 \| B-C \| |
| **Figure 4c COX-2** |
| \| Number of families \| 1 \|  \|  \|  \|  \|  \| \| --- \| --- \| --- \| --- \| --- \| --- \| --- \| \| Number of comparisons per family \| 3 \|  \|  \|  \|  \|  \| \| Alpha \| 0.05 \|  \|  \|  \|  \|  \| \|  \|  \|  \|  \|  \|  \|  \| \| Tukey's multiple comparisons test \| Mean Diff. \| 95.00% CI of diff. \| Significant? \| Summary \| Adjusted P Value \|  \| \|  \|  \|  \|  \|  \|  \|  \| \| C vs. L \| -1.269 \| -2.735 to 0.1968 \| No \| ns \| 0.0977 \| A-B \| \| C vs. L+Nil \| 0.244 \| -1.222 to 1.71 \| No \| ns \| 0.9080 \| A-C \| \| L vs. L+Nil \| 1.513 \| 0.04718 to 2.979 \| Yes \| * \| 0.0423 \| B-C \| |
| **Figure 4c IL-1β** |
| \| Number of families \| 1 \|  \|  \|  \|  \|  \| \| --- \| --- \| --- \| --- \| --- \| --- \| --- \| \| Number of comparisons per family \| 3 \|  \|  \|  \|  \|  \| \| Alpha \| 0.05 \|  \|  \|  \|  \|  \| \|  \|  \|  \|  \|  \|  \|  \| \| Tukey's multiple comparisons test \| Mean Diff. \| 95.00% CI of diff. \| Significant? \| Summary \| Adjusted P Value \|  \| \|  \|  \|  \|  \|  \|  \|  \| \| C vs. L \| -4.535 \| -8.208 to -0.862 \| Yes \| * \| 0.0140 \| A-B \| \| C vs. L+Nil \| -0.8579 \| -4.531 to 2.815 \| No \| ns \| 0.8276 \| A-C \| \| L vs. L+Nil \| 3.677 \| 0.004151 to 7.35 \| Yes \| * \| 0.0497 \| B-C \| |
| **Figure 4c IL-6** |
| \| Number of families \| 1 \|  \|  \|  \|  \|  \| \| --- \| --- \| --- \| --- \| --- \| --- \| --- \| \| Number of comparisons per family \| 3 \|  \|  \|  \|  \|  \| \| Alpha \| 0.05 \|  \|  \|  \|  \|  \| \|  \|  \|  \|  \|  \|  \|  \| \| Tukey's multiple comparisons test \| Mean Diff. \| 95.00% CI of diff. \| Significant? \| Summary \| Adjusted P Value \|  \| \|  \|  \|  \|  \|  \|  \|  \| \| C vs. L \| -175.2 \| -261.7 to -88.72 \| Yes \| *** \| 0.0001 \| A-B \| \| C vs. L+Nil \| -51.86 \| -138.3 to 34.61 \| No \| ns \| 0.3058 \| A-C \| \| L vs. L+Nil \| 123.3 \| 36.86 to 209.8 \| Yes \| ** \| 0.0046 \| B-C \| |
| **Figure 4c iNOS** |
| \| Number of families \| 1 \|  \|  \|  \|  \|  \| \| --- \| --- \| --- \| --- \| --- \| --- \| --- \| \| Number of comparisons per family \| 3 \|  \|  \|  \|  \|  \| \| Alpha \| 0.05 \|  \|  \|  \|  \|  \| \|  \|  \|  \|  \|  \|  \|  \| \| Tukey's multiple comparisons test \| Mean Diff. \| 95.00% CI of diff. \| Significant? \| Summary \| Adjusted P Value \|  \| \|  \|  \|  \|  \|  \|  \|  \| \| C vs. L \| -24.05 \| -40.77 to -7.33 \| Yes \| ** \| 0.0044 \| A-B \| \| C vs. L+Nil \| -6.449 \| -22.6 to 9.702 \| No \| ns \| 0.5791 \| A-C \| \| L vs. L+Nil \| 17.6 \| 0.881 to 34.32 \| Yes \| * \| 0.0380 \| B-C \| |
| **Figure 4d IL-4** |
| \| Number of families \| 1 \|  \|  \|  \|  \|  \| \| --- \| --- \| --- \| --- \| --- \| --- \| --- \| \| Number of comparisons per family \| 3 \|  \|  \|  \|  \|  \| \| Alpha \| 0.05 \|  \|  \|  \|  \|  \| \|  \|  \|  \|  \|  \|  \|  \| \| Tukey's multiple comparisons test \| Mean Diff. \| 95.00% CI of diff. \| Significant? \| Summary \| Adjusted P Value \|  \| \|  \|  \|  \|  \|  \|  \|  \| \| C vs. L \| 0.5231 \| -0.166 to 1.212 \| No \| ns \| 0.1597 \| A-B \| \| C vs. L+Nil \| 0.3335 \| -0.3556 to 1.023 \| No \| ns \| 0.4550 \| A-C \| \| L vs. L+Nil \| -0.1896 \| -0.8787 to 0.4995 \| No \| ns \| 0.7698 \| B-C \| |
| **Figure 4d IL-10** |
| \| Number of families \| 1 \|  \|  \|  \|  \|  \| \| --- \| --- \| --- \| --- \| --- \| --- \| --- \| \| Number of comparisons per family \| 3 \|  \|  \|  \|  \|  \| \| Alpha \| 0.05 \|  \|  \|  \|  \|  \| \|  \|  \|  \|  \|  \|  \|  \| \| Tukey's multiple comparisons test \| Mean Diff. \| 95.00% CI of diff. \| Significant? \| Summary \| Adjusted P Value \|  \| \|  \|  \|  \|  \|  \|  \|  \| \| C vs. L \| 0.2562 \| -0.8869 to 1.399 \| No \| ns \| 0.8400 \| A-B \| \| C vs. L+Nil \| -3.394 \| -4.537 to -2.251 \| Yes \| **** \| <0.0001 \| A-C \| \| L vs. L+Nil \| -3.65 \| -4.793 to -2.507 \| Yes \| **** \| <0.0001 \| B-C \| |
| **Figure 4e p-P38** |
| \| Number of families \| 1 \|  \|  \|  \|  \|  \| \| --- \| --- \| --- \| --- \| --- \| --- \| --- \| \| Number of comparisons per family \| 3 \|  \|  \|  \|  \|  \| \| Alpha \| 0.05 \|  \|  \|  \|  \|  \| \|  \|  \|  \|  \|  \|  \|  \| \| Tukey's multiple comparisons test \| Mean Diff. \| 95.00% CI of diff. \| Significant? \| Summary \| Adjusted P Value \|  \| \|  \|  \|  \|  \|  \|  \|  \| \| C vs. L \| -1350 \| -1879 to -820.2 \| Yes \| **** \| <0.0001 \| A-B \| \| C vs. L+Nil \| -144.5 \| -674 to 385.1 \| No \| ns \| 0.7732 \| A-C \| \| L vs. L+Nil \| 1205 \| 675.7 to 1735 \| Yes \| **** \| <0.0001 \| B-C \| |
| **Figure 4e P38** |
| \| Number of families \| 1 \|  \|  \|  \|  \|  \| \| --- \| --- \| --- \| --- \| --- \| --- \| --- \| \| Number of comparisons per family \| 3 \|  \|  \|  \|  \|  \| \| Alpha \| 0.05 \|  \|  \|  \|  \|  \| \|  \|  \|  \|  \|  \|  \|  \| \| Tukey's multiple comparisons test \| Mean Diff. \| 95.00% CI of diff. \| Significant? \| Summary \| Adjusted P Value \|  \| \|  \|  \|  \|  \|  \|  \|  \| \| C vs. L \| -2.451 \| -39.14 to 34.24 \| No \| ns \| 0.9845 \| A-B \| \| C vs. L+Nil \| 4.192 \| -32.5 to 40.88 \| No \| ns \| 0.9554 \| A-C \| \| L vs. L+Nil \| 6.643 \| -30.05 to 43.33 \| No \| ns \| 0.8921 \| B-C \| |
| **Figure 4f p-P38** |
| \| Number of families \| 1 \|  \|  \|  \|  \|  \| \| --- \| --- \| --- \| --- \| --- \| --- \| --- \| \| Number of comparisons per family \| 3 \|  \|  \|  \|  \|  \| \| Alpha \| 0.05 \|  \|  \|  \|  \|  \| \|  \|  \|  \|  \|  \|  \|  \| \| Tukey's multiple comparisons test \| Mean Diff. \| 95.00% CI of diff. \| Significant? \| Summary \| Adjusted P Value \|  \| \|  \|  \|  \|  \|  \|  \|  \| \| C vs. L \| -366.7 \| -391.9 to -341.4 \| Yes \| **** \| <0.0001 \| A-B \| \| C vs. L+Nil \| -38.41 \| -65.7 to -11.12 \| Yes \| ** \| 0.0028 \| A-C \| \| L vs. L+Nil \| 328.3 \| 300.3 to 356.3 \| Yes \| **** \| <0.0001 \| B-C \| |
| **Figure 4g p-STAT3^s727^** |
| \| Number of families \| 1 \|  \|  \|  \|  \|  \|  \| \| --- \| --- \| --- \| --- \| --- \| --- \| --- \| --- \| \| Number of comparisons per family \| 3 \|  \|  \|  \|  \|  \|  \| \| Alpha \| 0.05 \|  \|  \|  \|  \|  \|  \| \|  \|  \|  \|  \|  \|  \|  \|  \| \| Tukey's multiple comparisons test \| Mean Diff. \| 95.00% CI of diff. \| Significant? \| Summary \| Adjusted P Value \|  \|  \| \|  \|  \|  \|  \|  \|  \|  \|  \| \| C vs. L \| -39.39 \| -49.21 to -29.57 \| Yes \| **** \| <0.0001 \| A-B \|  \| \| C vs. L+Nil \| -10.44 \| -21.93 to 1.052 \| No \| ns \| 0.0839 \| A-C \|  \| \| L vs. L+Nil \| 28.95 \| 17.12 to 40.78 \| Yes \| **** \| <0.0001 \| B-C \|  \| |
| **Figure 4h SOD2** |
| \| Number of families \| 1 \|  \|  \|  \|  \|  \| \| --- \| --- \| --- \| --- \| --- \| --- \| --- \| \| Number of comparisons per family \| 3 \|  \|  \|  \|  \|  \| \| Alpha \| 0.05 \|  \|  \|  \|  \|  \| \|  \|  \|  \|  \|  \|  \|  \| \| Tukey's multiple comparisons test \| Mean Diff. \| 95.00% CI of diff. \| Significant? \| Summary \| Adjusted P Value \|  \| \|  \|  \|  \|  \|  \|  \|  \| \| C vs. L \| -11.26 \| -16.59 to -5.941 \| Yes \| **** \| <0.0001 \| A-B \| \| C vs. L+Nil \| -5.197 \| -10.52 to 0.126 \| No \| ns \| 0.0565 \| A-C \| \| L vs. L+Nil \| 6.067 \| 0.7444 to 11.39 \| Yes \| * \| 0.0237 \| B-C \| |
| **Figure 5b c-Abl** |
| \| Number of families \| 1 \|  \|  \|  \|  \|  \| \| --- \| --- \| --- \| --- \| --- \| --- \| --- \| \| Number of comparisons per family \| 3 \|  \|  \|  \|  \|  \| \| Alpha \| 0.05 \|  \|  \|  \|  \|  \| \|  \|  \|  \|  \|  \|  \|  \| \| Tukey's multiple comparisons test \| Mean Diff. \| 95.00% CI of diff. \| Significant? \| Summary \| Adjusted P Value \|  \| \|  \|  \|  \|  \|  \|  \|  \| \| C vs. L \| -64.07 \| -87.96 to -40.17 \| Yes \| **** \| <0.0001 \| A-B \| \| C vs. Nil+L \| -15.93 \| -39.99 to 8.135 \| No \| ns \| 0.2630 \| A-C \| \| L vs. Nil+L \| 48.14 \| 22.28 to 74 \| Yes \| **** \| <0.0001 \| B-C \| |
| **Figure 5c COX-2** |
| \| Number of families \| 1 \|  \|  \|  \|  \|  \| \| --- \| --- \| --- \| --- \| --- \| --- \| --- \| \| Number of comparisons per family \| 3 \|  \|  \|  \|  \|  \| \| Alpha \| 0.05 \|  \|  \|  \|  \|  \| \|  \|  \|  \|  \|  \|  \|  \| \| Tukey's multiple comparisons test \| Mean Diff. \| 95.00% CI of diff. \| Significant? \| Summary \| Adjusted P Value \|  \| \|  \|  \|  \|  \|  \|  \|  \| \| C vs. L \| -272.2 \| -367.8 to -176.6 \| Yes \| **** \| <0.0001 \| A-B \| \| C vs. Nil+L \| -42.06 \| -137.6 to 53.51 \| No \| ns \| 0.5191 \| A-C \| \| L vs. Nil+L \| 230.1 \| 134.5 to 325.7 \| Yes \| **** \| <0.0001 \| B-C \| |
| **Figure 5c IL-1β** |
| \| Number of families \| 1 \|  \|  \|  \|  \|  \| \| --- \| --- \| --- \| --- \| --- \| --- \| --- \| \| Number of comparisons per family \| 3 \|  \|  \|  \|  \|  \| \| Alpha \| 0.05 \|  \|  \|  \|  \|  \| \|  \|  \|  \|  \|  \|  \|  \| \| Tukey's multiple comparisons test \| Mean Diff. \| 95.00% CI of diff. \| Significant? \| Summary \| Adjusted P Value \|  \| \|  \|  \|  \|  \|  \|  \|  \| \| C vs. L \| -2204 \| -2628 to -1781 \| Yes \| **** \| <0.0001 \| A-B \| \| C vs. Nil+L \| -119.2 \| -542.7 to 304.4 \| No \| ns \| 0.7608 \| A-C \| \| L vs. Nil+L \| 2085 \| 1662 to 2509 \| Yes \| **** \| <0.0001 \| B-C \| |
| **Figure 5c IL-6** |
| \| Number of families \| 1 \|  \|  \|  \|  \|  \| \| --- \| --- \| --- \| --- \| --- \| --- \| --- \| \| Number of comparisons per family \| 3 \|  \|  \|  \|  \|  \| \| Alpha \| 0.05 \|  \|  \|  \|  \|  \| \|  \|  \|  \|  \|  \|  \|  \| \| Tukey's multiple comparisons test \| Mean Diff. \| 95.00% CI of diff. \| Significant? \| Summary \| Adjusted P Value \|  \| \|  \|  \|  \|  \|  \|  \|  \| \| C vs. L \| -323.4 \| -394 to -252.7 \| Yes \| **** \| <0.0001 \| A-B \| \| C vs. Nil+L \| -47.49 \| -118.1 to 23.17 \| No \| ns \| 0.2308 \| A-C \| \| L vs. Nil+L \| 275.9 \| 205.2 to 346.5 \| Yes \| **** \| <0.0001 \| B-C \| |
| **Figure 5c iNOS** |
| \| Number of families \| 1 \|  \|  \|  \|  \|  \| \| --- \| --- \| --- \| --- \| --- \| --- \| --- \| \| Number of comparisons per family \| 3 \|  \|  \|  \|  \|  \| \| Alpha \| 0.05 \|  \|  \|  \|  \|  \| \|  \|  \|  \|  \|  \|  \|  \| \| Tukey's multiple comparisons test \| Mean Diff. \| 95.00% CI of diff. \| Significant? \| Summary \| Adjusted P Value \|  \| \|  \|  \|  \|  \|  \|  \|  \| \| C vs. L \| -13.62 \| -17.85 to -9.379 \| Yes \| **** \| <0.0001 \| A-B \| \| C vs. Nil+L \| -5.56 \| -9.798 to -1.322 \| Yes \| ** \| 0.0090 \| A-C \| \| L vs. Nil+L \| 8.057 \| 3.818 to 12.29 \| Yes \| *** \| 0.0003 \| B-C \| |
| **Figure 5d c-Abl** |
| \| Number of families \| 1 \|  \|  \|  \|  \|  \| \| --- \| --- \| --- \| --- \| --- \| --- \| --- \| \| Number of comparisons per family \| 3 \|  \|  \|  \|  \|  \| \| Alpha \| 0.05 \|  \|  \|  \|  \|  \| \|  \|  \|  \|  \|  \|  \|  \| \| Tukey's multiple comparisons test \| Mean Diff. \| 95.00% CI of diff. \| Significant? \| Summary \| Adjusted P Value \|  \| \|  \|  \|  \|  \|  \|  \|  \| \| C vs. L \| -32.58 \| -57.43 to -7.741 \| Yes \| ** \| 0.0066 \| A-B \| \| C vs. L+Nil \| 22.35 \| -9.322 to 54.01 \| No \| ns \| 0.2183 \| A-C \| \| L vs. L+Nil \| 54.93 \| 22.57 to 87.29 \| Yes \| *** \| 0.0003 \| B-C \| |
| **Figure 5e IL-1β** |
| \| Number of families \| 1 \|  \|  \|  \|  \|  \|  \| \| --- \| --- \| --- \| --- \| --- \| --- \| --- \| --- \| \| Number of comparisons per family \| 3 \|  \|  \|  \|  \|  \|  \| \| Alpha \| 0.05 \|  \|  \|  \|  \|  \|  \| \|  \|  \|  \|  \|  \|  \|  \|  \| \| Tukey's multiple comparisons test \| Mean Diff. \| 95.00% CI of diff. \| Significant? \| Summary \| Adjusted P Value \|  \|  \| \|  \|  \|  \|  \|  \|  \|  \|  \| \| C vs. L \| -13.62 \| -19.33 to -7.904 \| Yes \| **** \| <0.0001 \| A-B \|  \| \| C vs. L+Nil \| -1.55 \| -7.262 to 4.162 \| No \| ns \| 0.7753 \| A-C \|  \| \| L vs. L+Nil \| 12.07 \| 6.354 to 17.78 \| Yes \| **** \| <0.0001 \| B-C \|  \| |
| **Figure 5e pro-IL-1β** |
| \| Number of families \| 1 \|  \|  \|  \|  \|  \| \| --- \| --- \| --- \| --- \| --- \| --- \| --- \| \| Number of comparisons per family \| 3 \|  \|  \|  \|  \|  \| \| Alpha \| 0.05 \|  \|  \|  \|  \|  \| \|  \|  \|  \|  \|  \|  \|  \| \| Tukey's multiple comparisons test \| Mean Diff. \| 95.00% CI of diff. \| Significant? \| Summary \| Adjusted P Value \|  \| \|  \|  \|  \|  \|  \|  \|  \| \| C vs. L \| -11.55 \| -16.39 to -6.724 \| Yes \| **** \| <0.0001 \| A-B \| \| C vs. L+Nil \| -0.3866 \| -5.217 to 4.444 \| No \| ns \| 0.9778 \| A-C \| \| L vs. L+Nil \| 11.17 \| 6.337 to 16 \| Yes \| **** \| <0.0001 \| B-C \| |
| **Figure 5e IL-6** |
| \| Number of families \| 1 \|  \|  \|  \|  \|  \| \| --- \| --- \| --- \| --- \| --- \| --- \| --- \| \| Number of comparisons per family \| 3 \|  \|  \|  \|  \|  \| \| Alpha \| 0.05 \|  \|  \|  \|  \|  \| \|  \|  \|  \|  \|  \|  \|  \| \| Tukey's multiple comparisons test \| Mean Diff. \| 95.00% CI of diff. \| Significant? \| Summary \| Adjusted P Value \|  \| \|  \|  \|  \|  \|  \|  \|  \| \| C vs. L \| -422.2 \| -510 to -334.4 \| Yes \| **** \| <0.0001 \| A-B \| \| C vs. L+Nil \| -88.83 \| -176.6 to -1.042 \| Yes \| * \| 0.0472 \| A-C \| \| L vs. L+Nil \| 333.4 \| 245.6 to 421.2 \| Yes \| **** \| <0.0001 \| B-C \| |
| **Figure 5e iNOS** |
| \| Number of families \| 1 \|  \|  \|  \|  \|  \| \| --- \| --- \| --- \| --- \| --- \| --- \| --- \| \| Number of comparisons per family \| 3 \|  \|  \|  \|  \|  \| \| Alpha \| 0.05 \|  \|  \|  \|  \|  \| \|  \|  \|  \|  \|  \|  \|  \| \| Tukey's multiple comparisons test \| Mean Diff. \| 95.00% CI of diff. \| Significant? \| Summary \| Adjusted P Value \|  \| \|  \|  \|  \|  \|  \|  \|  \| \| C vs. L \| -62.18 \| -97.97 to -26.38 \| Yes \| ** \| 0.0011 \| A-B \| \| C vs. L+Nil \| -83.88 \| -119.7 to -48.08 \| Yes \| **** \| <0.0001 \| A-C \| \| L vs. L+Nil \| -21.7 \| -57.49 to 14.1 \| No \| ns \| 0.2865 \| B-C \| |
| **Figure 6a p-P38** |
| \| Number of families \| 1 \|  \|  \|  \|  \|  \| \| --- \| --- \| --- \| --- \| --- \| --- \| --- \| \| Number of comparisons per family \| 3 \|  \|  \|  \|  \|  \| \| Alpha \| 0.05 \|  \|  \|  \|  \|  \| \|  \|  \|  \|  \|  \|  \|  \| \| Tukey's multiple comparisons test \| Mean Diff. \| 95.00% CI of diff. \| Significant? \| Summary \| Adjusted P Value \|  \| \|  \|  \|  \|  \|  \|  \|  \| \| C vs. L \| -88.97 \| -177.5 to -0.405 \| Yes \| * \| 0.0489 \| A-B \| \| C vs. L+Nil \| 5.783 \| -82.79 to 94.35 \| No \| ns \| 0.9834 \| A-C \| \| L vs. L+Nil \| 94.76 \| 6.188 to 183.3 \| Yes \| * \| 0.0360 \| B-C \| |
| **Figure 6a P38** |
| \| Number of families \| 1 \|  \|  \|  \|  \|  \| \| --- \| --- \| --- \| --- \| --- \| --- \| --- \| \| Number of comparisons per family \| 3 \|  \|  \|  \|  \|  \| \| Alpha \| 0.05 \|  \|  \|  \|  \|  \| \|  \|  \|  \|  \|  \|  \|  \| \| Tukey's multiple comparisons test \| Mean Diff. \| 95.00% CI of diff. \| Significant? \| Summary \| Adjusted P Value \|  \| \|  \|  \|  \|  \|  \|  \|  \| \| C vs. L \| 1.749 \| -19.88 to 23.38 \| No \| ns \| 0.9747 \| A-B \| \| C vs. L+Nil \| -14.92 \| -36.55 to 6.71 \| No \| ns \| 0.1985 \| A-C \| \| L vs. L+Nil \| -16.67 \| -38.3 to 4.961 \| No \| ns \| 0.1413 \| B-C \| |
| **Figure 6b p-P38** |
| \| Number of families \| 1 \|  \|  \|  \|  \|  \| \| --- \| --- \| --- \| --- \| --- \| --- \| --- \| \| Number of comparisons per family \| 3 \|  \|  \|  \|  \|  \| \| Alpha \| 0.05 \|  \|  \|  \|  \|  \| \|  \|  \|  \|  \|  \|  \|  \| \| Tukey's multiple comparisons test \| Mean Diff. \| 95.00% CI of diff. \| Significant? \| Summary \| Adjusted P Value \|  \| \|  \|  \|  \|  \|  \|  \|  \| \| C vs. L \| -75.56 \| -136.4 to -14.72 \| Yes \| * \| 0.0114 \| A-B \| \| C vs. L+Nil \| 13.65 \| -55.45 to 82.75 \| No \| ns \| 0.8830 \| A-C \| \| L vs. L+Nil \| 89.21 \| 25.96 to 152.5 \| Yes \| ** \| 0.0036 \| B-C \| |
| **Figure 6c p-AKT** |
| \| Number of families \| 1 \|  \|  \|  \|  \|  \| \| --- \| --- \| --- \| --- \| --- \| --- \| --- \| \| Number of comparisons per family \| 3 \|  \|  \|  \|  \|  \| \| Alpha \| 0.05 \|  \|  \|  \|  \|  \| \|  \|  \|  \|  \|  \|  \|  \| \| Tukey's multiple comparisons test \| Mean Diff. \| 95.00% CI of diff. \| Significant? \| Summary \| Adjusted P Value \|  \| \|  \|  \|  \|  \|  \|  \|  \| \| C vs. L \| -300.6 \| -515.7 to -85.56 \| Yes \| ** \| 0.0075 \| A-B \| \| C vs. L+Nil \| -86.33 \| -301.4 to 128.8 \| No \| ns \| 0.5490 \| A-C \| \| L vs. L+Nil \| 214.3 \| -0.7734 to 429.4 \| No \| ns \| 0.0509 \| B-C \| |
| **Figure 6c AKT** |
| \| Number of families \| 1 \|  \|  \|  \|  \|  \| \| --- \| --- \| --- \| --- \| --- \| --- \| --- \| \| Number of comparisons per family \| 3 \|  \|  \|  \|  \|  \| \| Alpha \| 0.05 \|  \|  \|  \|  \|  \| \|  \|  \|  \|  \|  \|  \|  \| \| Tukey's multiple comparisons test \| Mean Diff. \| 95.00% CI of diff. \| Significant? \| Summary \| Adjusted P Value \|  \| \|  \|  \|  \|  \|  \|  \|  \| \| C vs. L \| -99.41 \| -180.9 to -17.89 \| Yes \| * \| 0.0176 \| A-B \| \| C vs. L+Nil \| -98.4 \| -179.9 to -16.87 \| Yes \| * \| 0.0187 \| A-C \| \| L vs. L+Nil \| 1.017 \| -80.51 to 82.54 \| No \| ns \| 0.9994 \| B-C \| |
| **Figure 6d p-AKT** |
| \| Number of families \| 1 \|  \|  \|  \|  \|  \|  \| \| --- \| --- \| --- \| --- \| --- \| --- \| --- \| --- \| \| Number of comparisons per family \| 3 \|  \|  \|  \|  \|  \|  \| \| Alpha \| 0.05 \|  \|  \|  \|  \|  \|  \| \|  \|  \|  \|  \|  \|  \|  \|  \| \| Tukey's multiple comparisons test \| Mean Diff. \| 95.00% CI of diff. \| Significant? \| Summary \| Adjusted P Value \|  \|  \| \|  \|  \|  \|  \|  \|  \|  \|  \| \| C vs. L \| -92.42 \| -144.9 to -39.92 \| Yes \| *** \| 0.0002 \| A-B \|  \| \| C vs. L+Nil \| -114.1 \| -174 to -54.13 \| Yes \| **** \| <0.0001 \| A-C \|  \| \| L vs. L+Nil \| -21.64 \| -79.44 to 36.16 \| No \| ns \| 0.6442 \| B-C \|  \| \|  \|  \|  \|  \|  \|  \|  \|  \| |
| **Figure 6e p-STAT3^s727^** |
| \| Number of families \| 1 \|  \|  \|  \|  \|  \| \| --- \| --- \| --- \| --- \| --- \| --- \| --- \| \| Number of comparisons per family \| 3 \|  \|  \|  \|  \|  \| \| Alpha \| 0.05 \|  \|  \|  \|  \|  \| \|  \|  \|  \|  \|  \|  \|  \| \| Tukey's multiple comparisons test \| Mean Diff. \| 95.00% CI of diff. \| Significant? \| Summary \| Adjusted P Value \|  \| \|  \|  \|  \|  \|  \|  \|  \| \| C vs. L \| -157.7 \| -283.1 to -32.28 \| Yes \| * \| 0.0147 \| A-B \| \| C vs. L+Nil \| -25.91 \| -151.3 to 99.51 \| No \| ns \| 0.8478 \| A-C \| \| L vs. L+Nil \| 131.8 \| 6.368 to 257.2 \| Yes \| * \| 0.0394 \| B-C \| |
| **Figure 6e NF-kB** |
| \| Number of families \| 1 \|  \|  \|  \|  \|  \| \| --- \| --- \| --- \| --- \| --- \| --- \| --- \| \| Number of comparisons per family \| 3 \|  \|  \|  \|  \|  \| \| Alpha \| 0.05 \|  \|  \|  \|  \|  \| \|  \|  \|  \|  \|  \|  \|  \| \| Tukey's multiple comparisons test \| Mean Diff. \| 95.00% CI of diff. \| Significant? \| Summary \| Adjusted P Value \|  \| \|  \|  \|  \|  \|  \|  \|  \| \| C vs. L \| -57.56 \| -101 to -14.14 \| Yes \| * \| 0.0106 \| A-B \| \| C vs. L+Nil \| -18.18 \| -61.6 to 25.24 \| No \| ns \| 0.5222 \| A-C \| \| L vs. L+Nil \| 39.38 \| -4.044 to 82.8 \| No \| ns \| 0.0770 \| B-C \| |
| **Figure 6e PCNA** |
| \| Number of families \| 1 \|  \|  \|  \|  \|  \| \| --- \| --- \| --- \| --- \| --- \| --- \| --- \| \| Number of comparisons per family \| 3 \|  \|  \|  \|  \|  \| \| Alpha \| 0.05 \|  \|  \|  \|  \|  \| \|  \|  \|  \|  \|  \|  \|  \| \| Tukey's multiple comparisons test \| Mean Diff. \| 95.00% CI of diff. \| Significant? \| Summary \| Adjusted P Value \|  \| \|  \|  \|  \|  \|  \|  \|  \| \| C vs. L \| 4.642 \| -25.56 to 34.85 \| No \| ns \| 0.9122 \| A-B \| \| C vs. L+Nil \| -0.7423 \| -30.95 to 29.46 \| No \| ns \| 0.9976 \| A-C \| \| L vs. L+Nil \| -5.384 \| -35.59 to 24.82 \| No \| ns \| 0.8840 \| B-C \| |
| **Figure 6f p-STAT3^s727^** |
| \| Number of families \| 1 \|  \|  \|  \|  \|  \| \| --- \| --- \| --- \| --- \| --- \| --- \| --- \| \| Number of comparisons per family \| 3 \|  \|  \|  \|  \|  \| \| Alpha \| 0.05 \|  \|  \|  \|  \|  \| \|  \|  \|  \|  \|  \|  \|  \| \| Tukey's multiple comparisons test \| Mean Diff. \| 95.00% CI of diff. \| Significant? \| Summary \| Adjusted P Value \|  \| \|  \|  \|  \|  \|  \|  \|  \| \| C vs. L \| -85.86 \| -133.4 to -38.36 \| Yes \| **** \| <0.0001 \| A-B \| \| C vs. L+Nil \| 25.1 \| -19.23 to 69.43 \| No \| ns \| 0.3784 \| A-C \| \| L vs. L+Nil \| 111 \| 66.83 to 155.1 \| Yes \| **** \| <0.0001 \| B-C \| |
| **Figure 6g p-NF-kB^s536^** |
| \| Number of families \| 1 \|  \|  \|  \|  \|  \| \| --- \| --- \| --- \| --- \| --- \| --- \| --- \| \| Number of comparisons per family \| 3 \|  \|  \|  \|  \|  \| \| Alpha \| 0.05 \|  \|  \|  \|  \|  \| \|  \|  \|  \|  \|  \|  \|  \| \| Tukey's multiple comparisons test \| Mean Diff. \| 95.00% CI of diff. \| Significant? \| Summary \| Adjusted P Value \|  \| \|  \|  \|  \|  \|  \|  \|  \| \| C vs. L \| -59.6 \| -74.94 to -44.27 \| Yes \| **** \| <0.0001 \| A-B \| \| C vs. L+Nil \| -55.15 \| -70.76 to -39.55 \| Yes \| **** \| <0.0001 \| A-C \| \| L vs. L+Nil \| 4.45 \| -10.69 to 19.59 \| No \| ns \| 0.7692 \| B-C \| |

**Table S2.** t-tests or one-way ANOVA (Tukey’s test) and significance of the results of the *in vivo* experiments in this study.

| **Figure 7b c-Abl Cortex** | | | |
| --- | --- | --- | --- |
| \| Number of families \| 1 \|  \|  \|  \|  \|  \| \| --- \| --- \| --- \| --- \| --- \| --- \| --- \| \| Number of comparisons per family \| 3 \|  \|  \|  \|  \|  \| \| Alpha \| 0.05 \|  \|  \|  \|  \|  \| \|  \|  \|  \|  \|  \|  \|  \| \| Tukey's multiple comparisons test \| Mean Diff. \| 95.00% CI of diff. \| Significant? \| Summary \| Adjusted P Value \|  \| \|  \|  \|  \|  \|  \|  \|  \| \| **C** vs. L \| -1082 \| -1169 to -995.2 \| Yes \| **** \| <0.0001 \| A-B \| \| **C** vs. Nil+L \| -953.1 \| -1045 to -861.4 \| Yes \| **** \| <0.0001 \| A-C \| \| L vs. Nil+L \| 128.8 \| 32.87 to 224.7 \| Yes \| ** \| 0.0059 \| B-C \| | | | |
| **Figure 7b c-Abl CA1** | | | |
| \| Number of families \| 1 \|  \|  \|  \|  \|  \| \| --- \| --- \| --- \| --- \| --- \| --- \| --- \| \| Number of comparisons per family \| 3 \|  \|  \|  \|  \|  \| \| Alpha \| 0.05 \|  \|  \|  \|  \|  \| \|  \|  \|  \|  \|  \|  \|  \| \| Tukey's multiple comparisons test \| Mean Diff. \| 95.00% CI of diff. \| Significant? \| Summary \| Adjusted P Value \|  \| \|  \|  \|  \|  \|  \|  \|  \| \| **C** vs. L \| -509.6 \| -572.2 to -447.1 \| Yes \| **** \| <0.0001 \| D-E \| \| **C** vs. Nil+L \| -451.2 \| -526.7 to -375.6 \| Yes \| **** \| <0.0001 \| D-F \| \| L vs. Nil+L \| 58.46 \| -17.1 to 134 \| No \| ns \| 0.1590 \| E-F \| | | | |
| **Figure 7b c-Abl DG** | | | |
| \| Number of families \| 1 \|  \|  \|  \|  \|  \| \| --- \| --- \| --- \| --- \| --- \| --- \| --- \| \| Number of comparisons per family \| 3 \|  \|  \|  \|  \|  \| \| Alpha \| 0.05 \|  \|  \|  \|  \|  \| \|  \|  \|  \|  \|  \|  \|  \| \| Tukey's multiple comparisons test \| Mean Diff. \| 95.00% CI of diff. \| Significant? \| Summary \| Adjusted P Value \|  \| \|  \|  \|  \|  \|  \|  \|  \| \| **C** vs. L \| -374.4 \| -425.9 to -323 \| Yes \| **** \| <0.0001 \| G-H \| \| **C** vs. Nil+L \| -301.1 \| -363.3 to -238.9 \| Yes \| **** \| <0.0001 \| G-I \| \| L vs. Nil+L \| 73.38 \| 11.19 to 135.6 \| Yes \| * \| 0.0170 \| H-I \| | | | |
| **Figure 7b c-Abl CA3** | | | |
| \| Number of families \| 1 \|  \|  \|  \|  \|  \| \| --- \| --- \| --- \| --- \| --- \| --- \| --- \| \| Number of comparisons per family \| 3 \|  \|  \|  \|  \|  \| \| Alpha \| 0.05 \|  \|  \|  \|  \|  \| \|  \|  \|  \|  \|  \|  \|  \| \| Tukey's multiple comparisons test \| Mean Diff. \| 95.00% CI of diff. \| Significant? \| Summary \| Adjusted P Value \|  \| \|  \|  \|  \|  \|  \|  \|  \| \| **C** vs. L \| -573.3 \| -650.3 to -496.3 \| Yes \| **** \| <0.0001 \| J-K \| \| **C** vs. Nil+L \| -529.7 \| -622.6 to -436.7 \| Yes \| **** \| <0.0001 \| J-L \| \| L vs. Nil+L \| 43.68 \| -49.3 to 136.7 \| No \| ns \| 0.4990 \| K-L \| | | | |
| **Figure 7c c-Abl Cortex** | | | |
| \| Table Analyzed \| Bcr-Abl-Cortex \| \| --- \| --- \| \|  \|  \| \| Column B \| L \| \| vs. \| vs. \| \| Column A \| C \| \|  \|  \| \| Unpaired t test \|  \| \| P value \| 0.0329 \| \| P value summary \| * \| \| Significantly different (P < 0.05)? \| Yes \| \| One- or two-tailed P value? \| One-tailed \| \| t, df \| t=2.247 df=6 \| | | | \| Table Analyzed \| Bcr-Abl-Cortex \| \| --- \| --- \| \|  \|  \| \| Column C \| L+Nil \| \| vs. \| vs. \| \| Column B \| L \| \|  \|  \| \| Unpaired t test \|  \| \| P value \| 0.1774 \| \| P value summary \| ns \| \| Significantly different (P < 0.05)? \| No \| \| One- or two-tailed P value? \| One-tailed \| \| t, df \| t=1.002 df=6 \| |
| **Figure 7c c-Abl Hippocampus** | | | |
| \| Number of families \| 1 \|  \|  \|  \|  \|  \| \| --- \| --- \| --- \| --- \| --- \| --- \| --- \| \| Number of comparisons per family \| 3 \|  \|  \|  \|  \|  \| \| Alpha \| 0.05 \|  \|  \|  \|  \|  \| \|  \|  \|  \|  \|  \|  \|  \| \| Tukey's multiple comparisons test \| Mean Diff. \| 95.00% CI of diff. \| Significant? \| Summary \| Adjusted P Value \|  \| \|  \|  \|  \|  \|  \|  \|  \| \| C vs. L \| -83.46 \| -130.2 to -36.76 \| Yes \| * \| 0.0102 \| A-B \| \| C vs. L+Nil \| -13.93 \| -66.61 to 38.75 \| No \| ns \| 0.5742 \| A-C \| \| L vs. L+Nil \| 69.53 \| 13.58 to 125.5 \| Yes \| * \| 0.0281 \| B-C \| | | | |
| **Figure 8b Iba-1 Fluorescence intensity-Cortex** | | | |
| \| Number of families \| 1 \|  \|  \|  \|  \|  \| \| --- \| --- \| --- \| --- \| --- \| --- \| --- \| \| Number of comparisons per family \| 3 \|  \|  \|  \|  \|  \| \| Alpha \| 0.05 \|  \|  \|  \|  \|  \| \|  \|  \|  \|  \|  \|  \|  \| \| Tukey's multiple comparisons test \| Mean Diff. \| 95.00% CI of diff. \| Significant? \| Summary \| Adjusted P Value \|  \| \|  \|  \|  \|  \|  \|  \|  \| \| **C** vs. L \| -202.1 \| -332.8 to -71.31 \| Yes \| ** \| 0.0014 \| A-B \| \| **C** vs. Nil+L \| -38.38 \| -182.6 to 105.8 \| No \| ns \| 0.7978 \| A-C \| \| L vs. Nil+L \| 163.7 \| 23.89 to 303.5 \| Yes \| * \| 0.0180 \| B-C \| | | | |
| **Figure 8b Iba-1 Fluorescence intensity-CA1** | | | |
| \| Number of families \| 1 \|  \|  \|  \|  \|  \| \| --- \| --- \| --- \| --- \| --- \| --- \| --- \| \| Number of comparisons per family \| 3 \|  \|  \|  \|  \|  \| \| Alpha \| 0.05 \|  \|  \|  \|  \|  \| \|  \|  \|  \|  \|  \|  \|  \| \| Tukey's multiple comparisons test \| Mean Diff. \| 95.00% CI of diff. \| Significant? \| Summary \| Adjusted P Value \|  \| \|  \|  \|  \|  \|  \|  \|  \| \| **C** vs. L \| -282.8 \| -474.9 to -90.69 \| Yes \| ** \| 0.0024 \| D-E \| \| **C** vs. Nil+L \| -23.9 \| -227.1 to 179.3 \| No \| ns \| 0.9565 \| D-F \| \| L vs. Nil+L \| 258.9 \| 60.57 to 457.2 \| Yes \| ** \| 0.0075 \| E-F \| | | | |
| **Figure 8b Iba-1 Fluorescence intensity-DG** | | | |
| \| Number of families \| 1 \|  \|  \|  \|  \|  \| \| --- \| --- \| --- \| --- \| --- \| --- \| --- \| \| Number of comparisons per family \| 3 \|  \|  \|  \|  \|  \| \| Alpha \| 0.05 \|  \|  \|  \|  \|  \| \|  \|  \|  \|  \|  \|  \|  \| \| Tukey's multiple comparisons test \| Mean Diff. \| 95.00% CI of diff. \| Significant? \| Summary \| Adjusted P Value \|  \| \|  \|  \|  \|  \|  \|  \|  \| \| **C** vs. L \| -232.2 \| -434.7 to -29.72 \| Yes \| * \| 0.0210 \| G-H \| \| **C** vs. Nil+L \| -143.7 \| -357.8 to 70.4 \| No \| ns \| 0.2464 \| G-I \| \| L vs. Nil+L \| 88.47 \| -120.5 to 297.5 \| No \| ns \| 0.5667 \| H-I \| | | | |
| **Figure 8b Iba-1 Fluorescence intensity-CA3** | | | |
| \| Number of families \| 1 \|  \|  \|  \|  \|  \| \| --- \| --- \| --- \| --- \| --- \| --- \| --- \| \| Number of comparisons per family \| 3 \|  \|  \|  \|  \|  \| \| Alpha \| 0.05 \|  \|  \|  \|  \|  \| \|  \|  \|  \|  \|  \|  \|  \| \| Tukey's multiple comparisons test \| Mean Diff. \| 95.00% CI of diff. \| Significant? \| Summary \| Adjusted P Value \|  \| \|  \|  \|  \|  \|  \|  \|  \| \| **C** vs. L \| -20.75 \| -114.8 to 73.27 \| No \| ns \| 0.8556 \| J-K \| \| **C** vs. Nil+L \| 47.03 \| -52.41 to 146.5 \| No \| ns \| 0.4932 \| J-L \| \| L vs. Nil+L \| 67.79 \| -29.29 to 164.9 \| No \| ns \| 0.2205 \| K-L \| | | | |
| **Figure 8b Iba-1 Positive cells-Cortex** | | | |
| \| Number of families \| 1 \|  \|  \|  \|  \|  \| \| --- \| --- \| --- \| --- \| --- \| --- \| --- \| \| Number of comparisons per family \| 3 \|  \|  \|  \|  \|  \| \| Alpha \| 0.05 \|  \|  \|  \|  \|  \| \|  \|  \|  \|  \|  \|  \|  \| \| Tukey's multiple comparisons test \| Mean Diff. \| 95.00% CI of diff. \| Significant? \| Summary \| Adjusted P Value \|  \| \|  \|  \|  \|  \|  \|  \|  \| \| **C** vs. L \| -260.9 \| -380.4 to -141.5 \| Yes \| **** \| <0.0001 \| A-B \| \| **C** vs. Nil+L \| -21.88 \| -153.6 to 109.9 \| No \| ns \| 0.9156 \| A-C \| \| L vs. Nil+L \| 239.1 \| 111.4 to 366.8 \| Yes \| *** \| 0.0001 \| B-C \| | | | |
| **Figure 8b Iba-1 Positive cells-CA1** | | | |
| \| Number of families \| 1 \|  \|  \|  \|  \| \| --- \| --- \| --- \| --- \| --- \| --- \| \| Number of comparisons per family \| 3 \|  \|  \|  \|  \| \| Alpha \| 0.05 \|  \|  \|  \|  \| \|  \|  \|  \|  \|  \|  \| \| Newman-Keuls multiple comparisons test \| Mean Diff. \| Significant? \| Summary \|  \|  \| \|  \|  \|  \|  \|  \|  \| \| **C** vs. L \| -142.8 \| Yes \| * \|  \| D-E \| \| **C** vs. Nil+L \| -23.79 \| No \| ns \|  \| D-F \| \| L vs. Nil+L \| 119 \| Yes \| * \|  \| E-F \| | | | |
| **Figure 8b Iba-1 Positive cells-DG** | | | |
| \| Number of families \| 1 \|  \|  \|  \|  \|  \| \| --- \| --- \| --- \| --- \| --- \| --- \| --- \| \| Number of comparisons per family \| 3 \|  \|  \|  \|  \|  \| \| Alpha \| 0.05 \|  \|  \|  \|  \|  \| \|  \|  \|  \|  \|  \|  \|  \| \| Tukey's multiple comparisons test \| Mean Diff. \| 95.00% CI of diff. \| Significant? \| Summary \| Adjusted P Value \|  \| \|  \|  \|  \|  \|  \|  \|  \| \| **C** vs. L \| -158.3 \| -318.6 to 2 \| No \| ns \| 0.0536 \| G-H \| \| **C** vs. Nil+L \| -95.16 \| -264.7 to 74.36 \| No \| ns \| 0.3719 \| G-I \| \| L vs. Nil+L \| 63.13 \| -102.4 to 228.6 \| No \| ns \| 0.6297 \| H-I \| | | | |
| **Figure 8b Iba-1 Positive cells-CA3** | | | |
| \| Number of families \| 1 \|  \|  \|  \|  \|  \| \| --- \| --- \| --- \| --- \| --- \| --- \| --- \| \| Number of comparisons per family \| 3 \|  \|  \|  \|  \|  \| \| Alpha \| 0.05 \|  \|  \|  \|  \|  \| \|  \|  \|  \|  \|  \|  \|  \| \| Tukey's multiple comparisons test \| Mean Diff. \| 95.00% CI of diff. \| Significant? \| Summary \| Adjusted P Value \|  \| \|  \|  \|  \|  \|  \|  \|  \| \| **C** vs. L \| -40.34 \| -320.3 to 239.6 \| No \| ns \| 0.9356 \| J-K \| \| **C** vs. Nil+L \| 11.08 \| -285 to 307.1 \| No \| ns \| 0.9955 \| J-L \| \| L vs. Nil+L \| 51.42 \| -237.6 to 340.4 \| No \| ns \| 0.9035 \| K-L \| | | | |
| **Figure 8b Iba-1 % of area-Cortex** | | | |
| \| Number of families \| 1 \|  \|  \|  \|  \|  \| \| --- \| --- \| --- \| --- \| --- \| --- \| --- \| \| Number of comparisons per family \| 3 \|  \|  \|  \|  \|  \| \| Alpha \| 0.05 \|  \|  \|  \|  \|  \| \|  \|  \|  \|  \|  \|  \|  \| \| Tukey's multiple comparisons test \| Mean Diff. \| 95.00% CI of diff. \| Significant? \| Summary \| Adjusted P Value \|  \| \|  \|  \|  \|  \|  \|  \|  \| \| **C** vs. L \| -1.486 \| -2.248 to -0.7244 \| Yes \| **** \| <0.0001 \| A-B \| \| **C** vs. Nil+L \| -0.2024 \| -1.042 to 0.6375 \| No \| ns \| 0.8308 \| A-C \| \| L vs. Nil+L \| 1.284 \| 0.4694 to 2.098 \| Yes \| ** \| 0.0011 \| B-C \| | | | |
| **Figure 8b Iba-1 % of area-CA1** | | | |
| \| Number of families \| 1 \|  \|  \|  \|  \|  \| \| --- \| --- \| --- \| --- \| --- \| --- \| --- \| \| Number of comparisons per family \| 3 \|  \|  \|  \|  \|  \| \| Alpha \| 0.05 \|  \|  \|  \|  \|  \| \|  \|  \|  \|  \|  \|  \|  \| \| Tukey's multiple comparisons test \| Mean Diff. \| 95.00% CI of diff. \| Significant? \| Summary \| Adjusted P Value \|  \| \|  \|  \|  \|  \|  \|  \|  \| \| **C** vs. L \| -0.6875 \| -1.157 to -0.2184 \| Yes \| ** \| 0.0025 \| D-E \| \| **C** vs. Nil+L \| -0.1605 \| -0.6566 to 0.3356 \| No \| ns \| 0.7163 \| D-F \| \| L vs. Nil+L \| 0.527 \| 0.04271 to 1.011 \| Yes \| * \| 0.0300 \| E-F \| | | | |
| **Figure 8b Iba-1 % of area-DG** | | | |
| \| Number of families \| 1 \|  \|  \|  \|  \|  \| \| --- \| --- \| --- \| --- \| --- \| --- \| --- \| \| Number of comparisons per family \| 3 \|  \|  \|  \|  \|  \| \| Alpha \| 0.05 \|  \|  \|  \|  \|  \| \|  \|  \|  \|  \|  \|  \|  \| \| Tukey's multiple comparisons test \| Mean Diff. \| 95.00% CI of diff. \| Significant? \| Summary \| Adjusted P Value \|  \| \|  \|  \|  \|  \|  \|  \|  \| \| **C** vs. L \| -1.116 \| -2.123 to -0.1098 \| Yes \| * \| 0.0265 \| G-H \| \| **C** vs. Nil+L \| -0.4156 \| -1.48 to 0.6489 \| No \| ns \| 0.6162 \| G-I \| \| L vs. Nil+L \| 0.7008 \| -0.3384 to 1.74 \| No \| ns \| 0.2433 \| H-I \| | | | |
| **Figure 8b Iba-1 % of area-CA3** | | | |
| \| Number of families \| 1 \|  \|  \|  \|  \|  \| \| --- \| --- \| --- \| --- \| --- \| --- \| --- \| \| Number of comparisons per family \| 3 \|  \|  \|  \|  \|  \| \| Alpha \| 0.05 \|  \|  \|  \|  \|  \| \|  \|  \|  \|  \|  \|  \|  \| \| Tukey's multiple comparisons test \| Mean Diff. \| 95.00% CI of diff. \| Significant? \| Summary \| Adjusted P Value \|  \| \|  \|  \|  \|  \|  \|  \|  \| \| **C** vs. L \| -0.08327 \| -2.037 to 1.871 \| No \| ns \| 0.9942 \| J-K \| \| **C** vs. Nil+L \| 1.057 \| -1.009 to 3.123 \| No \| ns \| 0.4385 \| J-L \| \| L vs. Nil+L \| 1.14 \| -0.8769 to 3.157 \| No \| ns \| 0.3669 \| K-L \| | | | |
| **Figure 8d GFAP Fluorescence intensity-Cortex** | | | |
| \| Number of families \| 1 \|  \|  \|  \|  \|  \| \| --- \| --- \| --- \| --- \| --- \| --- \| --- \| \| Number of comparisons per family \| 3 \|  \|  \|  \|  \|  \| \| Alpha \| 0.05 \|  \|  \|  \|  \|  \| \|  \|  \|  \|  \|  \|  \|  \| \| Tukey's multiple comparisons test \| Mean Diff. \| 95.00% CI of diff. \| Significant? \| Summary \| Adjusted P Value \|  \| \|  \|  \|  \|  \|  \|  \|  \| \| **C** vs. L \| -31.31 \| -132.4 to 69.81 \| No \| ns \| 0.7370 \| A-B \| \| **C** vs. Nil+L \| -5.219 \| -106.3 to 95.9 \| No \| ns \| 0.9915 \| A-C \| \| L vs. Nil+L \| 26.09 \| -58.87 to 111 \| No \| ns \| 0.7407 \| B-C \| | | | |
| **Figure 8d GFAP Fluorescence intensity-CA1** | | | |
| \| Number of families \| 1 \|  \|  \|  \|  \|  \| \| --- \| --- \| --- \| --- \| --- \| --- \| --- \| \| Number of comparisons per family \| 3 \|  \|  \|  \|  \|  \| \| Alpha \| 0.05 \|  \|  \|  \|  \|  \| \|  \|  \|  \|  \|  \|  \|  \| \| Tukey's multiple comparisons test \| Mean Diff. \| 95.00% CI of diff. \| Significant? \| Summary \| Adjusted P Value \|  \| \|  \|  \|  \|  \|  \|  \|  \| \| **C** vs. L \| -94.25 \| -174.7 to -13.78 \| Yes \| * \| 0.0181 \| D-E \| \| **C** vs. Nil+L \| -78.24 \| -161.8 to 5.323 \| No \| ns \| 0.0708 \| D-F \| \| L vs. Nil+L \| 16.01 \| -63.13 to 95.15 \| No \| ns \| 0.8770 \| E-F \| | | | |
| **Figure 8d GFAP Fluorescence intensity-DG** | | | |
| \| Number of families \| 1 \|  \|  \|  \|  \|  \| \| --- \| --- \| --- \| --- \| --- \| --- \| --- \| \| Number of comparisons per family \| 3 \|  \|  \|  \|  \|  \| \| Alpha \| 0.05 \|  \|  \|  \|  \|  \| \|  \|  \|  \|  \|  \|  \|  \| \| Tukey's multiple comparisons test \| Mean Diff. \| 95.00% CI of diff. \| Significant? \| Summary \| Adjusted P Value \|  \| \|  \|  \|  \|  \|  \|  \|  \| \| **C** vs. L \| -60.19 \| -121.5 to 1.163 \| No \| ns \| 0.0556 \| G-H \| \| **C** vs. Nil+L \| -9.64 \| -72.49 to 53.21 \| No \| ns \| 0.9273 \| G-I \| \| L vs. Nil+L \| 50.55 \| -8.88 to 110 \| No \| ns \| 0.1100 \| H-I \| | | | |
| **Figure 8d GFAP Fluorescence intensity-CA3** | | | |
| \| Number of families \| 1 \|  \|  \|  \|  \|  \| \| --- \| --- \| --- \| --- \| --- \| --- \| --- \| \| Number of comparisons per family \| 3 \|  \|  \|  \|  \|  \| \| Alpha \| 0.05 \|  \|  \|  \|  \|  \| \|  \|  \|  \|  \|  \|  \|  \| \| Tukey's multiple comparisons test \| Mean Diff. \| 95.00% CI of diff. \| Significant? \| Summary \| Adjusted P Value \|  \| \|  \|  \|  \|  \|  \|  \|  \| \| **C** vs. L \| -8.681 \| -69.46 to 52.1 \| No \| ns \| 0.9366 \| J-K \| \| **C** vs. Nil+L \| 46.18 \| -15.37 to 107.7 \| No \| ns \| 0.1760 \| J-L \| \| L vs. Nil+L \| 54.86 \| -4.059 to 113.8 \| No \| ns \| 0.0728 \| K-L \| | | | |
| **Figure 8d GFAP Positive cells-Cortex** | | | |
| \| Number of families \| 1 \|  \|  \|  \|  \|  \| \| --- \| --- \| --- \| --- \| --- \| --- \| --- \| \| Number of comparisons per family \| 3 \|  \|  \|  \|  \|  \| \| Alpha \| 0.05 \|  \|  \|  \|  \|  \| \|  \|  \|  \|  \|  \|  \|  \| \| Tukey's multiple comparisons test \| Mean Diff. \| 95.00% CI of diff. \| Significant? \| Summary \| Adjusted P Value \|  \| \|  \|  \|  \|  \|  \|  \|  \| \| **C** vs. L \| -70.23 \| -164.6 to 24.17 \| No \| ns \| 0.1815 \| A-B \| \| **C** vs. Nil+L \| 2.948 \| -91.45 to 97.35 \| No \| ns \| 0.9969 \| A-C \| \| L vs. Nil+L \| 73.18 \| -6.135 to 152.5 \| No \| ns \| 0.0762 \| B-C \| | | | |
| **Figure 8d GFAP Positive cells-CA1** | | | |
| \| Number of families \| 1 \|  \|  \|  \|  \|  \| \| --- \| --- \| --- \| --- \| --- \| --- \| --- \| \| Number of comparisons per family \| 3 \|  \|  \|  \|  \|  \| \| Alpha \| 0.05 \|  \|  \|  \|  \|  \| \|  \|  \|  \|  \|  \|  \|  \| \| Tukey's multiple comparisons test \| Mean Diff. \| 95.00% CI of diff. \| Significant? \| Summary \| Adjusted P Value \|  \| \|  \|  \|  \|  \|  \|  \|  \| \| **C** vs. L \| -861710 \| -2347123 to 623704 \| No \| ns \| 0.3480 \| D-E \| \| **C** vs. Nil+L \| -1488786 \| -3031352 to 53780 \| No \| ns \| 0.0606 \| D-F \| \| L vs. Nil+L \| -627076 \| -2088017 to 833864 \| No \| ns \| 0.5575 \| E-F \| | | | |
| **Figure 8d GFAP Positive cells-DG** | | | |
| \| Number of families \| 1 \|  \|  \|  \|  \|  \| \| --- \| --- \| --- \| --- \| --- \| --- \| --- \| \| Number of comparisons per family \| 3 \|  \|  \|  \|  \|  \| \| Alpha \| 0.05 \|  \|  \|  \|  \|  \| \|  \|  \|  \|  \|  \|  \|  \| \| Tukey's multiple comparisons test \| Mean Diff. \| 95.00% CI of diff. \| Significant? \| Summary \| Adjusted P Value \|  \| \|  \|  \|  \|  \|  \|  \|  \| \| **C** vs. L \| -2407438 \| -4728531 to -86345 \| Yes \| * \| 0.0405 \| G-H \| \| **C** vs. Nil+L \| -1480151 \| -3857864 to 897562 \| No \| ns \| 0.2982 \| G-I \| \| L vs. Nil+L \| 927287 \| -1321026 to 3175600 \| No \| ns \| 0.5830 \| H-I \| | | | |
| **Figure 8d GFAP Positive cells-CA3** | | | |
| \| Number of families \| 1 \|  \|  \|  \|  \|  \| \| --- \| --- \| --- \| --- \| --- \| --- \| --- \| \| Number of comparisons per family \| 3 \|  \|  \|  \|  \|  \| \| Alpha \| 0.05 \|  \|  \|  \|  \|  \| \|  \|  \|  \|  \|  \|  \|  \| \| Tukey's multiple comparisons test \| Mean Diff. \| 95.00% CI of diff. \| Significant? \| Summary \| Adjusted P Value \|  \| \|  \|  \|  \|  \|  \|  \|  \| \| **C** vs. L \| -3134456 \| -5945349 to -323563 \| Yes \| * \| 0.0256 \| J-K \| \| **C** vs. Nil+L \| -186338 \| -3032701 to 2660025 \| No \| ns \| 0.9863 \| J-L \| \| L vs. Nil+L \| 2948118 \| 223316 to 5672920 \| Yes \| * \| 0.0312 \| K-L \| | | | |
| **Figure 8d GFAP % of area-Cortex** | | | |
| \| Number of families \| 1 \|  \|  \|  \|  \| \| --- \| --- \| --- \| --- \| --- \| --- \| \| Number of comparisons per family \| 3 \|  \|  \|  \|  \| \| Alpha \| 0.05 \|  \|  \|  \|  \| \|  \|  \|  \|  \|  \|  \| \| Newman-Keuls multiple comparisons test \| Mean Diff. \| Significant? \| Summary \|  \|  \| \|  \|  \|  \|  \|  \|  \| \| **C** vs. L \| -0.4035 \| Yes \| ** \|  \| A-B \| \| **C** vs. Nil+L \| -0.1487 \| No \| ns \|  \| A-C \| \| L vs. Nil+L \| 0.2548 \| Yes \| * \|  \| B-C \| | | | |
| **Figure 8d GFAP % of area-CA1** | | | |
| \| Number of families \| 1 \|  \|  \|  \|  \| \| --- \| --- \| --- \| --- \| --- \| --- \| \| Number of comparisons per family \| 3 \|  \|  \|  \|  \| \| Alpha \| 0.05 \|  \|  \|  \|  \| \|  \|  \|  \|  \|  \|  \| \| Newman-Keuls multiple comparisons test \| Mean Diff. \| Significant? \| Summary \|  \|  \| \|  \|  \|  \|  \|  \|  \| \| **C** vs. L \| -0.4035 \| Yes \| ** \|  \| A-B \| \| **C** vs. Nil+L \| -0.1487 \| No \| ns \|  \| A-C \| \| L vs. Nil+L \| 0.2548 \| Yes \| * \|  \| B-C \| | | | |
| **Figure 8d GFAP % of area-DG** | | | |
| \| Number of families \| 1 \|  \|  \|  \|  \| \| --- \| --- \| --- \| --- \| --- \| --- \| \| Number of comparisons per family \| 3 \|  \|  \|  \|  \| \| Alpha \| 0.05 \|  \|  \|  \|  \| \|  \|  \|  \|  \|  \|  \| \| Newman-Keuls multiple comparisons test \| Mean Diff. \| Significant? \| Summary \|  \|  \| \|  \|  \|  \|  \|  \|  \| \| **C** vs. L \| -8.241 \| Yes \| * \|  \| G-H \| \| **C** vs. Nil+L \| -1.694 \| No \| ns \|  \| G-I \| \| L vs. Nil+L \| 6.547 \| Yes \| * \|  \| H-I \| | | | |
| **Figure 8d GFAP % of area-CA3** | | | |
| \| Number of families \| 1 \|  \|  \|  \|  \|  \| \| --- \| --- \| --- \| --- \| --- \| --- \| --- \| \| Number of comparisons per family \| 3 \|  \|  \|  \|  \|  \| \| Alpha \| 0.05 \|  \|  \|  \|  \|  \| \|  \|  \|  \|  \|  \|  \|  \| \| Tukey's multiple comparisons test \| Mean Diff. \| 95.00% CI of diff. \| Significant? \| Summary \| Adjusted P Value \|  \| \|  \|  \|  \|  \|  \|  \|  \| \| **C** vs. L \| -0.7738 \| -6.498 to 4.951 \| No \| ns \| 0.9430 \| J-K \| \| **C** vs. Nil+L \| 5.181 \| -0.6156 to 10.98 \| No \| ns \| 0.0884 \| J-L \| \| L vs. Nil+L \| 5.955 \| 0.4057 to 11.5 \| Yes \| * \| 0.0328 \| K-L \| | | | |
| **Figure 9a IL-1β-Cortex** | | | |
| \| Number of families \| 1 \|  \|  \|  \|  \|  \| \| --- \| --- \| --- \| --- \| --- \| --- \| --- \| \| Number of comparisons per family \| 3 \|  \|  \|  \|  \|  \| \| Alpha \| 0.05 \|  \|  \|  \|  \|  \| \|  \|  \|  \|  \|  \|  \|  \| \| Tukey's multiple comparisons test \| Mean Diff. \| 95.00% CI of diff. \| Significant? \| Summary \| Adjusted P Value \|  \| \|  \|  \|  \|  \|  \|  \|  \| \| - vs. + \| -20.33 \| -35.6 to -5.062 \| Yes \| ** \| 0.0081 \| A-B \| \| - vs. + \| -24.28 \| -39.55 to -9.012 \| Yes \| ** \| 0.0018 \| A-C \| \| + vs. + \| -3.951 \| -19.22 to 11.32 \| No \| ns \| 0.7931 \| B-C \| | | | |
| **Figure 9a IL-1β-Hippocampus** | | | |
| \| Number of families \| 1 \|  \|  \|  \|  \|  \| \| --- \| --- \| --- \| --- \| --- \| --- \| --- \| \| Number of comparisons per family \| 3 \|  \|  \|  \|  \|  \| \| Alpha \| 0.05 \|  \|  \|  \|  \|  \| \|  \|  \|  \|  \|  \|  \|  \| \| Tukey's multiple comparisons test \| Mean Diff. \| 95.00% CI of diff. \| Significant? \| Summary \| Adjusted P Value \|  \| \|  \|  \|  \|  \|  \|  \|  \| \| - vs. + \| -80.83 \| -121.5 to -40.11 \| Yes \| *** \| 0.0002 \| E-F \| \| - vs. + \| -30.91 \| -71.63 to 9.801 \| No \| ns \| 0.1595 \| E-G \| \| + vs. + \| 49.91 \| 9.198 to 90.63 \| Yes \| * \| 0.0147 \| F-G \| | | | |
| **Figure 9b IL-6-Cortex** | | | |
| \| Number of families \| 1 \|  \|  \|  \|  \|  \| \| --- \| --- \| --- \| --- \| --- \| --- \| --- \| \| Number of comparisons per family \| 3 \|  \|  \|  \|  \|  \| \| Alpha \| 0.05 \|  \|  \|  \|  \|  \| \|  \|  \|  \|  \|  \|  \|  \| \| Tukey's multiple comparisons test \| Mean Diff. \| 95.00% CI of diff. \| Significant? \| Summary \| Adjusted P Value \|  \| \|  \|  \|  \|  \|  \|  \|  \| \| - vs. + \| -2.295 \| -3.695 to -0.896 \| Yes \| ** \| 0.0013 \| A-B \| \| - vs. + \| -2.857 \| -4.257 to -1.458 \| Yes \| *** \| 0.0001 \| A-C \| \| + vs. + \| -0.5619 \| -1.961 to 0.8375 \| No \| ns \| 0.5776 \| B-C \| | | | |
| **Figure 9b IL-6-Hippocampus** | | | |
| \| Number of families \| 1 \|  \|  \|  \|  \|  \| \| --- \| --- \| --- \| --- \| --- \| --- \| --- \| \| Number of comparisons per family \| 3 \|  \|  \|  \|  \|  \| \| Alpha \| 0.05 \|  \|  \|  \|  \|  \| \|  \|  \|  \|  \|  \|  \|  \| \| Tukey's multiple comparisons test \| Mean Diff. \| 95.00% CI of diff. \| Significant? \| Summary \| Adjusted P Value \|  \| \|  \|  \|  \|  \|  \|  \|  \| \| - vs. + \| -23.37 \| -30.95 to -15.79 \| Yes \| **** \| <0.0001 \| E-F \| \| - vs. + \| -16.87 \| -24.45 to -9.281 \| Yes \| **** \| <0.0001 \| E-G \| \| + vs. + \| 6.504 \| -1.079 to 14.09 \| No \| ns \| 0.1015 \| F-G \| | | | |
| **Figure 9c COX-2-Cortex** | | | |
| \| Number of families \| 1 \|  \|  \|  \|  \|  \| \| --- \| --- \| --- \| --- \| --- \| --- \| --- \| \| Number of comparisons per family \| 3 \|  \|  \|  \|  \|  \| \| Alpha \| 0.05 \|  \|  \|  \|  \|  \| \|  \|  \|  \|  \|  \|  \|  \| \| Tukey's multiple comparisons test \| Mean Diff. \| 95.00% CI of diff. \| Significant? \| Summary \| Adjusted P Value \|  \| \|  \|  \|  \|  \|  \|  \|  \| \| - vs. + \| -1.031 \| -1.694 to -0.3672 \| Yes \| ** \| 0.0022 \| A-B \| \| - vs. + \| -0.5019 \| -1.165 to 0.1615 \| No \| ns \| 0.1615 \| A-C \| \| + vs. + \| 0.5288 \| -0.1346 to 1.192 \| No \| ns \| 0.1347 \| B-C \| | | | |
| \| Table Analyzed \| COX-2 \| \| --- \| --- \| \|  \|  \| \| Column C \| + \| \| vs. \| vs. \| \| Column B \| + \| \|  \|  \| \| Unpaired t test \|  \| \| P value \| 0.0403 \| \| P value summary \| * \| \| Significantly different (P < 0.05)? \| Yes \| \| One- or two-tailed P value? \| One-tailed \| \| t, df \| t=1.884 df=14 \| | | | |
| **Figure 9c COX-2-Hippocampus** | | | |
| \| Number of families \| 1 \|  \|  \|  \|  \|  \| \| --- \| --- \| --- \| --- \| --- \| --- \| --- \| \| Number of comparisons per family \| 3 \|  \|  \|  \|  \|  \| \| Alpha \| 0.05 \|  \|  \|  \|  \|  \| \|  \|  \|  \|  \|  \|  \|  \| \| Tukey's multiple comparisons test \| Mean Diff. \| 95.00% CI of diff. \| Significant? \| Summary \| Adjusted P Value \|  \| \|  \|  \|  \|  \|  \|  \|  \| \| - vs. + \| -0.7337 \| -1.608 to 0.1409 \| No \| ns \| 0.1110 \| E-F \| \| - vs. + \| 0.1709 \| -0.7037 to 1.046 \| No \| ns \| 0.8756 \| E-G \| \| + vs. + \| 0.9046 \| 0.02998 to 1.779 \| Yes \| * \| 0.0418 \| F-G \| | | | |
| **Figure 9d IL-1β Fluorescence intensity-Cortex** | | | |
| \| Number of families \| 1 \|  \|  \|  \|  \|  \| \| --- \| --- \| --- \| --- \| --- \| --- \| --- \| \| Number of comparisons per family \| 3 \|  \|  \|  \|  \|  \| \| Alpha \| 0.05 \|  \|  \|  \|  \|  \| \|  \|  \|  \|  \|  \|  \|  \| \| Tukey's multiple comparisons test \| Mean Diff. \| 95.00% CI of diff. \| Significant? \| Summary \| Adjusted P Value \|  \| \|  \|  \|  \|  \|  \|  \|  \| \| **C** vs. L \| -37.45 \| -70.37 to -4.53 \| Yes \| * \| 0.0223 \| A-B \| \| **C** vs. Nil+L \| 10.58 \| -20.65 to 41.81 \| No \| ns \| 0.6934 \| A-C \| \| L vs. Nil+L \| 48.03 \| 16.8 to 79.26 \| Yes \| ** \| 0.0015 \| B-C \| | | | |
| **Figure 9d IL-1β Fluorescence intensity-CA1** | | | |
| \| Number of families \| 1 \|  \|  \|  \|  \| \| --- \| --- \| --- \| --- \| --- \| --- \| \| Number of comparisons per family \| 3 \|  \|  \|  \|  \| \| Alpha \| 0.05 \|  \|  \|  \|  \| \|  \|  \|  \|  \|  \|  \| \| Newman-Keuls multiple comparisons test \| Mean Diff. \| Significant? \| Summary \|  \|  \| \|  \|  \|  \|  \|  \|  \| \| **C** vs. L \| -51.24 \| Yes \| * \|  \| D-E \| \| **C** vs. Nil+L \| -11.24 \| No \| ns \|  \| D-F \| \| L vs. Nil+L \| 40 \| Yes \| * \|  \| E-F \| | | | |
| **Figure 9d IL-1β Fluorescence intensity-DG** | | | |
| \| Number of families \| 1 \|  \|  \|  \|  \| \| --- \| --- \| --- \| --- \| --- \| --- \| \| Number of comparisons per family \| 3 \|  \|  \|  \|  \| \| Alpha \| 0.05 \|  \|  \|  \|  \| \|  \|  \|  \|  \|  \|  \| \| Newman-Keuls multiple comparisons test \| Mean Diff. \| Significant? \| Summary \|  \|  \| \|  \|  \|  \|  \|  \|  \| \| **C** vs. L \| -38.4 \| Yes \| * \|  \| G-H \| \| **C** vs. Nil+L \| -11.65 \| No \| ns \|  \| G-I \| \| L vs. Nil+L \| 26.75 \| No \| ns \|  \| H-I \| | | | |
| **Figure 9d IL-1β Fluorescence intensity-CA3** | | | |
| \| Number of families \| 1 \|  \|  \|  \|  \| \| --- \| --- \| --- \| --- \| --- \| --- \| \| Number of comparisons per family \| 3 \|  \|  \|  \|  \| \| Alpha \| 0.05 \|  \|  \|  \|  \| \|  \|  \|  \|  \|  \|  \| \| Newman-Keuls multiple comparisons test \| Mean Diff. \| Significant? \| Summary \|  \|  \| \|  \|  \|  \|  \|  \|  \| \| **C** vs. L \| -43.73 \| Yes \| * \|  \| J-K \| \| **C** vs. Nil+L \| -11.38 \| No \| ns \|  \| J-L \| \| L vs. Nil+L \| 32.35 \| Yes \| * \|  \| K-L \| | | | |
| **Figure 9e IL-6 Fluorescence intensity-Cortex** | | | |
| \| Number of families \| 1 \|  \|  \|  \|  \|  \| \| --- \| --- \| --- \| --- \| --- \| --- \| --- \| \| Number of comparisons per family \| 3 \|  \|  \|  \|  \|  \| \| Alpha \| 0.05 \|  \|  \|  \|  \|  \| \|  \|  \|  \|  \|  \|  \|  \| \| Tukey's multiple comparisons test \| Mean Diff. \| 95.00% CI of diff. \| Significant? \| Summary \| Adjusted P Value \|  \| \|  \|  \|  \|  \|  \|  \|  \| \| **C** vs. L \| -57.07 \| -81.37 to -32.77 \| Yes \| **** \| <0.0001 \| A-B \| \| **C** vs. Nil+L \| -1.26 \| -25.29 to 22.77 \| No \| ns \| 0.9913 \| A-C \| \| L vs. Nil+L \| 55.81 \| 32.09 to 79.53 \| Yes \| **** \| <0.0001 \| B-C \| | | | |
| **Figure 9e IL-6 Fluorescence intensity-CA1** | | | |
| \| Number of families \| 1 \|  \|  \|  \|  \| \| --- \| --- \| --- \| --- \| --- \| --- \| \| Number of comparisons per family \| 3 \|  \|  \|  \|  \| \| Alpha \| 0.05 \|  \|  \|  \|  \| \|  \|  \|  \|  \|  \|  \| \| Newman-Keuls multiple comparisons test \| Mean Diff. \| Significant? \| Summary \|  \|  \| \|  \|  \|  \|  \|  \|  \| \| **C** vs. L \| -75.32 \| Yes \| **** \|  \| D-E \| \| **C** vs. Nil+L \| -17.3 \| No \| ns \|  \| D-F \| \| L vs. Nil+L \| 58.02 \| Yes \| **** \|  \| E-F \| | | | |
| **Figure 9e IL-6 Fluorescence intensity-DG** | | | |
| \| Number of families \| 1 \|  \|  \|  \|  \| \| --- \| --- \| --- \| --- \| --- \| --- \| \| Number of comparisons per family \| 3 \|  \|  \|  \|  \| \| Alpha \| 0.05 \|  \|  \|  \|  \| \|  \|  \|  \|  \|  \|  \| \| Newman-Keuls multiple comparisons test \| Mean Diff. \| Significant? \| Summary \|  \|  \| \|  \|  \|  \|  \|  \|  \| \| **C** vs. L \| -64.19 \| Yes \| **** \|  \| G-H \| \| **C** vs. Nil+L \| -16.9 \| No \| ns \|  \| G-I \| \| L vs. Nil+L \| 47.29 \| Yes \| **** \|  \| H-I \| | | | |
| **Figure 9e IL-6 Fluorescence intensity-CA3** | | | |
| \| Number of families \| 1 \|  \|  \|  \|  \| \| --- \| --- \| --- \| --- \| --- \| --- \| \| Number of comparisons per family \| 3 \|  \|  \|  \|  \| \| Alpha \| 0.05 \|  \|  \|  \|  \| \|  \|  \|  \|  \|  \|  \| \| Newman-Keuls multiple comparisons test \| Mean Diff. \| Significant? \| Summary \|  \|  \| \|  \|  \|  \|  \|  \|  \| \| **C** vs. L \| -58.92 \| Yes \| **** \|  \| J-K \| \| **C** vs. Nil+L \| -22.29 \| No \| ns \|  \| J-L \| \| L vs. Nil+L \| 36.62 \| Yes \| ** \|  \| K-L \| | | | |
| **Figure 9f COX-2 Fluorescence intensity-Cortex** | | | |
| \| Number of families \| 1 \|  \|  \|  \|  \|  \| \| --- \| --- \| --- \| --- \| --- \| --- \| --- \| \| Number of comparisons per family \| 3 \|  \|  \|  \|  \|  \| \| Alpha \| 0.05 \|  \|  \|  \|  \|  \| \|  \|  \|  \|  \|  \|  \|  \| \| Tukey's multiple comparisons test \| Mean Diff. \| 95.00% CI of diff. \| Significant? \| Summary \| Adjusted P Value \|  \| \|  \|  \|  \|  \|  \|  \|  \| \| **C** vs. L \| -42.14 \| -56.21 to -28.08 \| Yes \| **** \| <0.0001 \| A-B \| \| **C** vs. Nil+L \| 1.266 \| -13.69 to 16.22 \| No \| ns \| 0.9776 \| A-C \| \| L vs. Nil+L \| 43.41 \| 29.73 to 57.09 \| Yes \| **** \| <0.0001 \| B-C \| | | | |
| **Figure 9f COX-2 Fluorescence intensity-CA1** | | | |
| \| Number of families \| 1 \|  \|  \|  \|  \| \| --- \| --- \| --- \| --- \| --- \| --- \| \| Number of comparisons per family \| 3 \|  \|  \|  \|  \| \| Alpha \| 0.05 \|  \|  \|  \|  \| \|  \|  \|  \|  \|  \|  \| \| Newman-Keuls multiple comparisons test \| Mean Diff. \| Significant? \| Summary \|  \|  \| \|  \|  \|  \|  \|  \|  \| \| **C** vs. L \| -33.52 \| Yes \| **** \|  \| D-E \| \| **C** vs. Nil+L \| -0.7559 \| No \| ns \|  \| D-F \| \| L vs. Nil+L \| 32.76 \| Yes \| **** \|  \| E-F \| | | | |
| **Figure 9f COX-2 Fluorescence intensity-DG** | | | |
| \| Number of families \| 1 \|  \|  \|  \|  \| \| --- \| --- \| --- \| --- \| --- \| --- \| \| Number of comparisons per family \| 3 \|  \|  \|  \|  \| \| Alpha \| 0.05 \|  \|  \|  \|  \| \|  \|  \|  \|  \|  \|  \| \| Newman-Keuls multiple comparisons test \| Mean Diff. \| Significant? \| Summary \|  \|  \| \|  \|  \|  \|  \|  \|  \| \| **C** vs. L \| -46.8 \| Yes \| **** \|  \| G-H \| \| **C** vs. Nil+L \| -17.87 \| Yes \| * \|  \| G-I \| \| L vs. Nil+L \| 28.93 \| Yes \| **** \|  \| H-I \| | | | |
| **Figure 9f COX-2 Fluorescence intensity-CA3** | | | |
| \| Number of families \| 1 \|  \|  \|  \|  \| \| --- \| --- \| --- \| --- \| --- \| --- \| \| Number of comparisons per family \| 3 \|  \|  \|  \|  \| \| Alpha \| 0.05 \|  \|  \|  \|  \| \|  \|  \|  \|  \|  \|  \| \| Newman-Keuls multiple comparisons test \| Mean Diff. \| Significant? \| Summary \|  \|  \| \|  \|  \|  \|  \|  \|  \| \| **C** vs. L \| -33.05 \| Yes \| **** \|  \| J-K \| \| **C** vs. Nil+L \| -11.96 \| No \| ns \|  \| J-L \| \| L vs. Nil+L \| 21.09 \| Yes \| *** \|  \| K-L \| | | | |
| **Figure 9g IL-1β-Cortex** | | | |
| \| Number of families \| 1 \|  \|  \|  \|  \|  \| \| --- \| --- \| --- \| --- \| --- \| --- \| --- \| \| Number of comparisons per family \| 3 \|  \|  \|  \|  \|  \| \| Alpha \| 0.05 \|  \|  \|  \|  \|  \| \|  \|  \|  \|  \|  \|  \|  \| \| Tukey's multiple comparisons test \| Mean Diff. \| 95.00% CI of diff. \| Significant? \| Summary \| Adjusted P Value \|  \| \|  \|  \|  \|  \|  \|  \|  \| \| - vs. + \| -178.8 \| -233.6 to -123.9 \| Yes \| **** \| <0.0001 \| A-B \| \| - vs. + \| -111.6 \| -166.5 to -56.74 \| Yes \| *** \| 0.0001 \| A-C \| \| + vs. + \| 67.15 \| 12.28 to 122 \| Yes \| * \| 0.0149 \| B-C \| | | | |
| **Figure 9g IL-1β -Hippocampus** | | | |
| \| Number of families \| 1 \|  \|  \|  \|  \|  \| \| --- \| --- \| --- \| --- \| --- \| --- \| --- \| \| Number of comparisons per family \| 3 \|  \|  \|  \|  \|  \| \| Alpha \| 0.05 \|  \|  \|  \|  \|  \| \|  \|  \|  \|  \|  \|  \|  \| \| Tukey's multiple comparisons test \| Mean Diff. \| 95.00% CI of diff. \| Significant? \| Summary \| Adjusted P Value \|  \| \|  \|  \|  \|  \|  \|  \|  \| \| - vs. + \| -146.2 \| -199.6 to -92.78 \| Yes \| **** \| <0.0001 \| E-F \| \| - vs. + \| -85.1 \| -138.5 to -31.7 \| Yes \| ** \| 0.0017 \| E-G \| \| + vs. + \| 61.09 \| 7.677 to 114.5 \| Yes \| * \| 0.0232 \| F-G \| | | | |
| **Figure 9h IL-6-Cortex** | | | |
| \| Number of families \| 1 \|  \|  \|  \|  \|  \| \| --- \| --- \| --- \| --- \| --- \| --- \| --- \| \| Number of comparisons per family \| 3 \|  \|  \|  \|  \|  \| \| Alpha \| 0.05 \|  \|  \|  \|  \|  \| \|  \|  \|  \|  \|  \|  \|  \| \| Tukey's multiple comparisons test \| Mean Diff. \| 95.00% CI of diff. \| Significant? \| Summary \| Adjusted P Value \|  \| \|  \|  \|  \|  \|  \|  \|  \| \| - vs. + \| -590.7 \| -739.1 to -442.3 \| Yes \| **** \| <0.0001 \| A-B \| \| - vs. + \| -392.7 \| -541.1 to -244.3 \| Yes \| **** \| <0.0001 \| A-C \| \| + vs. + \| 198.1 \| 49.67 to 346.4 \| Yes \| ** \| 0.0079 \| B-C \| | | | |
| **Figure 9h IL-6-Hippocampus** | | | |
| \| Number of families \| 1 \|  \|  \|  \|  \|  \| \| --- \| --- \| --- \| --- \| --- \| --- \| --- \| \| Number of comparisons per family \| 3 \|  \|  \|  \|  \|  \| \| Alpha \| 0.05 \|  \|  \|  \|  \|  \| \|  \|  \|  \|  \|  \|  \|  \| \| Tukey's multiple comparisons test \| Mean Diff. \| 95.00% CI of diff. \| Significant? \| Summary \| Adjusted P Value \|  \| \|  \|  \|  \|  \|  \|  \|  \| \| - vs. + \| -528 \| -643.8 to -412.2 \| Yes \| **** \| <0.0001 \| E-F \| \| - vs. + \| -313.6 \| -429.4 to -197.8 \| Yes \| **** \| <0.0001 \| E-G \| \| + vs. + \| 214.4 \| 98.56 to 330.2 \| Yes \| *** \| 0.0004 \| F-G \| | | | |
| **Figure 9i COX-2-Cortex** | | | |
| \| Number of families \| 1 \|  \|  \|  \|  \|  \| \| --- \| --- \| --- \| --- \| --- \| --- \| --- \| \| Number of comparisons per family \| 3 \|  \|  \|  \|  \|  \| \| Alpha \| 0.05 \|  \|  \|  \|  \|  \| \|  \|  \|  \|  \|  \|  \|  \| \| Tukey's multiple comparisons test \| Mean Diff. \| 95.00% CI of diff. \| Significant? \| Summary \| Adjusted P Value \|  \| \|  \|  \|  \|  \|  \|  \|  \| \| - vs. + \| -19.87 \| -43.71 to 3.956 \| No \| ns \| 0.1136 \| A-B \| \| - vs. + \| 32.53 \| 8.7 to 56.36 \| Yes \| ** \| 0.0066 \| A-C \| \| + vs. + \| 52.41 \| 28.57 to 76.24 \| Yes \| **** \| <0.0001 \| B-C \| | | | |
| **Figure 9i COX-2-Hippocampus** | | | |
| \| Number of families \| 1 \|  \|  \|  \|  \|  \| \| --- \| --- \| --- \| --- \| --- \| --- \| --- \| \| Number of comparisons per family \| 3 \|  \|  \|  \|  \|  \| \| Alpha \| 0.05 \|  \|  \|  \|  \|  \| \|  \|  \|  \|  \|  \|  \|  \| \| Tukey's multiple comparisons test \| Mean Diff. \| 95.00% CI of diff. \| Significant? \| Summary \| Adjusted P Value \|  \| \|  \|  \|  \|  \|  \|  \|  \| \| - vs. + \| -30.5 \| -60.84 to -0.1637 \| Yes \| * \| 0.0486 \| E-F \| \| - vs. + \| 51.08 \| 20.74 to 81.42 \| Yes \| ** \| 0.0010 \| E-G \| \| + vs. + \| 81.59 \| 51.25 to 111.9 \| Yes \| **** \| <0.0001 \| F-G \| | | | |
| **Figure 10b p-P38 Fluorescence intensity-Cortex** | | | |
| \| Number of families \| 1 \|  \|  \|  \|  \|  \| \| --- \| --- \| --- \| --- \| --- \| --- \| --- \| \| Number of comparisons per family \| 3 \|  \|  \|  \|  \|  \| \| Alpha \| 0.05 \|  \|  \|  \|  \|  \| \|  \|  \|  \|  \|  \|  \|  \| \| Tukey's multiple comparisons test \| Mean Diff. \| 95.00% CI of diff. \| Significant? \| Summary \| Adjusted P Value \|  \| \|  \|  \|  \|  \|  \|  \|  \| \| **C** vs. L \| -102.6 \| -131.7 to -73.47 \| Yes \| **** \| <0.0001 \| A-B \| \| **C** vs. Nil+L \| -2.214 \| -30.57 to 26.14 \| No \| ns \| 0.9807 \| A-C \| \| L vs. Nil+L \| 100.3 \| 75.15 to 125.5 \| Yes \| **** \| <0.0001 \| B-C \| | | | |
| **Figure 10b p-P38 Fluorescence intensity-CA1** | | | |
| \| Number of families \| 1 \|  \|  \|  \|  \| \| --- \| --- \| --- \| --- \| --- \| --- \| \| Number of comparisons per family \| 3 \|  \|  \|  \|  \| \| Alpha \| 0.05 \|  \|  \|  \|  \| \|  \|  \|  \|  \|  \|  \| \| Newman-Keuls multiple comparisons test \| Mean Diff. \| Significant? \| Summary \|  \|  \| \|  \|  \|  \|  \|  \|  \| \| **C** vs. L \| -97.68 \| Yes \| **** \|  \| D-E \| \| **C** vs. Nil+L \| 8.796 \| No \| ns \|  \| D-F \| \| L vs. Nil+L \| 106.5 \| Yes \| **** \|  \| E-F \| | | | |
| **Figure 10b p-P38 Fluorescence intensity-DG** | | | |
| \| Number of families \| 1 \|  \|  \|  \|  \| \| --- \| --- \| --- \| --- \| --- \| --- \| \| Number of comparisons per family \| 3 \|  \|  \|  \|  \| \| Alpha \| 0.05 \|  \|  \|  \|  \| \|  \|  \|  \|  \|  \|  \| \| Newman-Keuls multiple comparisons test \| Mean Diff. \| Significant? \| Summary \|  \|  \| \|  \|  \|  \|  \|  \|  \| \| **C** vs. L \| -98.27 \| Yes \| **** \|  \| G-H \| \| **C** vs. Nil+L \| 5.275 \| No \| ns \|  \| G-I \| \| L vs. Nil+L \| 103.5 \| Yes \| **** \|  \| H-I \| | | | |
| **Figure 10b p-P38 Fluorescence intensity-CA3** | | | |
| \| Number of families \| 1 \|  \|  \|  \|  \| \| --- \| --- \| --- \| --- \| --- \| --- \| \| Number of comparisons per family \| 3 \|  \|  \|  \|  \| \| Alpha \| 0.05 \|  \|  \|  \|  \| \|  \|  \|  \|  \|  \|  \| \| Newman-Keuls multiple comparisons test \| Mean Diff. \| Significant? \| Summary \|  \|  \| \|  \|  \|  \|  \|  \|  \| \| **C** vs. L \| -91.7 \| Yes \| **** \|  \| J-K \| \| **C** vs. Nil+L \| 5.101 \| No \| ns \|  \| J-L \| \| L vs. Nil+L \| 96.8 \| Yes \| **** \|  \| K-L \| | | | |
| **Figure 10d p-STAT3^s727^ Fluorescence intensity-Cortex** | | | |
| \| Number of families \| 1 \|  \|  \|  \|  \|  \| \| --- \| --- \| --- \| --- \| --- \| --- \| --- \| \| Number of comparisons per family \| 3 \|  \|  \|  \|  \|  \| \| Alpha \| 0.05 \|  \|  \|  \|  \|  \| \|  \|  \|  \|  \|  \|  \|  \| \| Tukey's multiple comparisons test \| Mean Diff. \| 95.00% CI of diff. \| Significant? \| Summary \| Adjusted P Value \|  \| \|  \|  \|  \|  \|  \|  \|  \| \| **C** vs. L \| -228.5 \| -328.3 to -128.7 \| Yes \| **** \| <0.0001 \| A-B \| \| **C** vs. Nil+L \| -106.2 \| -204 to -8.467 \| Yes \| * \| 0.0301 \| A-C \| \| L vs. Nil+L \| 122.2 \| 25.67 to 218.8 \| Yes \| ** \| 0.0095 \| B-C \| | | | |
| **Figure 10d p-STAT3^s727^ Fluorescence intensity-CA1** | | | |
| \| Number of families \| 1 \|  \|  \|  \|  \| \| --- \| --- \| --- \| --- \| --- \| --- \| \| Number of comparisons per family \| 3 \|  \|  \|  \|  \| \| Alpha \| 0.05 \|  \|  \|  \|  \| \|  \|  \|  \|  \|  \|  \| \| Newman-Keuls multiple comparisons test \| Mean Diff. \| Significant? \| Summary \|  \|  \| \|  \|  \|  \|  \|  \|  \| \| **C** vs. L \| -178.1 \| Yes \| **** \|  \| D-E \| \| **C** vs. Nil+L \| -104.9 \| Yes \| ** \|  \| D-F \| \| L vs. Nil+L \| 73.14 \| Yes \| * \|  \| E-F \| | | | |
| **Figure 10d p-STAT3^s727^ Fluorescence intensity-DG** | | | |
| \| Number of families \| 1 \|  \|  \|  \|  \| \| --- \| --- \| --- \| --- \| --- \| --- \| \| Number of comparisons per family \| 3 \|  \|  \|  \|  \| \| Alpha \| 0.05 \|  \|  \|  \|  \| \|  \|  \|  \|  \|  \|  \| \| Newman-Keuls multiple comparisons test \| Mean Diff. \| Significant? \| Summary \|  \|  \| \|  \|  \|  \|  \|  \|  \| \| **C** vs. L \| -146 \| Yes \| **** \|  \| G-H \| \| **C** vs. Nil+L \| -105 \| Yes \| ** \|  \| G-I \| \| L vs. Nil+L \| 41.06 \| No \| ns \|  \| H-I \| | | | |
| **Figure 10d p-STAT3^s727^ Fluorescence intensity-CA3** | | | |
| \| Number of families \| 1 \|  \|  \|  \|  \| \| --- \| --- \| --- \| --- \| --- \| --- \| \| Number of comparisons per family \| 3 \|  \|  \|  \|  \| \| Alpha \| 0.05 \|  \|  \|  \|  \| \|  \|  \|  \|  \|  \|  \| \| Newman-Keuls multiple comparisons test \| Mean Diff. \| Significant? \| Summary \|  \|  \| \|  \|  \|  \|  \|  \|  \| \| **C** vs. L \| -102.1 \| Yes \| *** \|  \| J-K \| \| **C** vs. Nil+L \| -73.77 \| Yes \| ** \|  \| J-L \| \| L vs. Nil+L \| 28.31 \| No \| ns \|  \| K-L \| | | | |
| **Figure 10e p-P38-Cortex** | | | |
| \| Table Analyzed \| pp38-Cortex \| \| --- \| --- \| \|  \|  \| \| Column C \| L+Nil \| \| vs. \| vs. \| \| Column B \| L \| \|  \|  \| \| Paired t test \|  \| \| P value \| 0.0253 \| \| P value summary \| * \| \| Significantly different (P < 0.05)? \| Yes \| \| One- or two-tailed P value? \| One-tailed \| \| t, df \| t=3.166 df=3 \| \| Number of pairs \| 4 \| | | \| Table Analyzed \| pp38-Cortex \| \| --- \| --- \| \|  \|  \| \| Column B \| L \| \| vs. \| vs. \| \| Column A \| C \| \|  \|  \| \| Unpaired t test \|  \| \| P value \| 0.0306 \| \| P value summary \| * \| \| Significantly different (P < 0.05)? \| Yes \| \| One- or two-tailed P value? \| One-tailed \| \| t, df \| t=2.299 df=6 \| | |
| **Figure 10e p-P38-Hippocampus** | | | |
| \| Table Analyzed \| pp38-Hippo \| \| --- \| --- \| \|  \|  \| \| Column B \| L \| \| vs. \| vs. \| \| Column A \| C \| \|  \|  \| \| Paired t test \|  \| \| P value \| 0.0305 \| \| P value summary \| * \| \| Significantly different (P < 0.05)? \| Yes \| \| One- or two-tailed P value? \| Two-tailed \| \| t, df \| t=3.871 df=3 \| \| Number of pairs \| 4 \| | | \| Table Analyzed \| pp38-Hippo \| \| --- \| --- \| \|  \|  \| \| Column C \| L+Nil \| \| vs. \| vs. \| \| Column B \| L \| \|  \|  \| \| Paired t test \|  \| \| P value \| 0.0491 \| \| P value summary \| * \| \| Significantly different (P < 0.05)? \| Yes \| \| One- or two-tailed P value? \| One-tailed \| \| t, df \| t=2.374 df=3 \| \| Number of pairs \| 4 \| | |
| **Figure 10f p-STAT3^s727^-Cortex** | | | |
| \| Table Analyzed \| pSTAT3-Cortex \| \| --- \| --- \| \|  \|  \| \| Column B \| L \| \| vs. \| vs. \| \| Column A \| C \| \|  \|  \| \| Unpaired t test \|  \| \| P value \| 0.0352 \| \| P value summary \| * \| \| Significantly different (P < 0.05)? \| Yes \| \| One- or two-tailed P value? \| One-tailed \| \| t, df \| t=2.197 df=6 \| | \| Table Analyzed \| pSTAT3-Cortex \| \| --- \| --- \| \|  \|  \| \| Column C \| L+Nil \| \| vs. \| vs. \| \| Column B \| L \| \|  \|  \| \| Paired t test \|  \| \| P value \| 0.0304 \| \| P value summary \| * \| \| Significantly different (P < 0.05)? \| Yes \| \| One- or two-tailed P value? \| One-tailed \| \| t, df \| t=2.934 df=3 \| \| Number of pairs \| 4 \| | | |
| **Figure 10f p-STAT3^s727^-Hippocampus** | | | |
| \| Table Analyzed \| pSTAT3-Hippo \| \| --- \| --- \| \|  \|  \| \| Column B \| L \| \| vs. \| vs. \| \| Column A \| C \| \|  \|  \| \| Unpaired t test \|  \| \| P value \| 0.0034 \| \| P value summary \| ** \| \| Significantly different (P < 0.05)? \| Yes \| \| One- or two-tailed P value? \| Two-tailed \| \| t, df \| t=4.676 df=6 \| | | | \| Table Analyzed \| pSTAT3-Hippo \| \| --- \| --- \| \|  \|  \| \| Column C \| L+Nil \| \| vs. \| vs. \| \| Column B \| L \| \|  \|  \| \| Unpaired t test \|  \| \| P value \| 0.0418 \| \| P value summary \| * \| \| Significantly different (P < 0.05)? \| Yes \| \| One- or two-tailed P value? \| One-tailed \| \| t, df \| t=2.072 df=6 \| |
| **Figure 11a Y-maze Spontaneous alteration** | | | |
| \| Number of families \| 1 \|  \|  \|  \|  \|  \| \| --- \| --- \| --- \| --- \| --- \| --- \| --- \| \| Number of comparisons per family \| 3 \|  \|  \|  \|  \|  \| \| Alpha \| 0.05 \|  \|  \|  \|  \|  \| \|  \|  \|  \|  \|  \|  \|  \| \| Tukey's multiple comparisons test \| Mean Diff. \| 95.00% CI of diff. \| Significant? \| Summary \| Adjusted P Value \|  \| \|  \|  \|  \|  \|  \|  \|  \| \| C vs. L \| 14.18 \| 0.06809 to 28.28 \| Yes \| * \| 0.0488 \| A-B \| \| C vs. Nil+L \| -5.6 \| -18.98 to 7.783 \| No \| ns \| 0.5551 \| A-C \| \| L vs. Nil+L \| -19.78 \| -33.16 to -6.392 \| Yes \| ** \| 0.0033 \| B-C \| | | | |
| **Figure 11a Y-maze Number of total entries** | | | |
| \| Number of families \| 1 \|  \|  \|  \|  \|  \| \| --- \| --- \| --- \| --- \| --- \| --- \| --- \| \| Number of comparisons per family \| 3 \|  \|  \|  \|  \|  \| \| Alpha \| 0.05 \|  \|  \|  \|  \|  \| \|  \|  \|  \|  \|  \|  \|  \| \| Tukey's multiple comparisons test \| Mean Diff. \| 95.00% CI of diff. \| Significant? \| Summary \| Adjusted P Value \|  \| \|  \|  \|  \|  \|  \|  \|  \| \| C vs. L \| 4.75 \| -1.67 to 11.17 \| No \| ns \| 0.1752 \| A-B \| \| C vs. Nil+L \| 0.775 \| -5.316 to 6.866 \| No \| ns \| 0.9457 \| A-C \| \| L vs. Nil+L \| -3.975 \| -10.07 to 2.116 \| No \| ns \| 0.2518 \| B-C \| | | | |
| **Figure 11b NOR Training** | | | |
| \| Number of families \| 1 \|  \|  \|  \|  \|  \| \| --- \| --- \| --- \| --- \| --- \| --- \| --- \| \| Number of comparisons per family \| 3 \|  \|  \|  \|  \|  \| \| Alpha \| 0.05 \|  \|  \|  \|  \|  \| \|  \|  \|  \|  \|  \|  \|  \| \| Tukey's multiple comparisons test \| Mean Diff. \| 95.00% CI of diff. \| Significant? \| Summary \| Adjusted P Value \|  \| \|  \|  \|  \|  \|  \|  \|  \| \| C vs. L \| 8.829 \| -7.159 to 24.82 \| No \| ns \| 0.3707 \| A-B \| \| C vs. Nil+L \| 15.1 \| -0.1064 to 30.3 \| No \| ns \| 0.0519 \| A-C \| \| L vs. Nil+L \| 6.268 \| -9.372 to 21.91 \| No \| ns \| 0.5871 \| B-C \| | | | |
| **Figure 11b NOR Test** | | | |
| \| Number of families \| 1 \|  \|  \|  \|  \|  \| \| --- \| --- \| --- \| --- \| --- \| --- \| --- \| \| Number of comparisons per family \| 3 \|  \|  \|  \|  \|  \| \| Alpha \| 0.05 \|  \|  \|  \|  \|  \| \|  \|  \|  \|  \|  \|  \|  \| \| Tukey's multiple comparisons test \| Mean Diff. \| 95.00% CI of diff. \| Significant? \| Summary \| Adjusted P Value \|  \| \|  \|  \|  \|  \|  \|  \|  \| \| C vs. L \| 13.63 \| -4.201 to 31.45 \| No \| ns \| 0.1573 \| E-F \| \| C vs. Nil+L \| -0.8765 \| -18.17 to 16.42 \| No \| ns \| 0.9912 \| E-G \| \| L vs. Nil+L \| -14.5 \| -32.33 to 3.324 \| No \| ns \| 0.1259 \| F-G \| | | | |
| **Figure 11c Golgi Cortex AO** | | | |
| \| Number of families \| 1 \|  \|  \|  \|  \|  \| \| --- \| --- \| --- \| --- \| --- \| --- \| --- \| \| Number of comparisons per family \| 3 \|  \|  \|  \|  \|  \| \| Alpha \| 0.05 \|  \|  \|  \|  \|  \| \|  \|  \|  \|  \|  \|  \|  \| \| Tukey's multiple comparisons test \| Mean Diff. \| 95.00% CI of diff. \| Significant? \| Summary \| Adjusted P Value \|  \| \|  \|  \|  \|  \|  \|  \|  \| \| C vs. L \| 5.394 \| 2.853 to 7.934 \| Yes \| **** \| <0.0001 \| A-B \| \| C vs. Nil+L \| 1.302 \| -1.239 to 3.842 \| No \| ns \| 0.4431 \| A-C \| \| L vs. Nil+L \| -4.092 \| -6.761 to -1.423 \| Yes \| ** \| 0.0013 \| B-C \| \|  \|  \|  \|  \|  \|  \|  \| | | | |
| **Figure 11c Golgi Cortex BS** | | | |
| \| Number of families \| 1 \|  \|  \|  \|  \|  \| \| --- \| --- \| --- \| --- \| --- \| --- \| --- \| \| Number of comparisons per family \| 3 \|  \|  \|  \|  \|  \| \| Alpha \| 0.05 \|  \|  \|  \|  \|  \| \|  \|  \|  \|  \|  \|  \|  \| \| Tukey's multiple comparisons test \| Mean Diff. \| 95.00% CI of diff. \| Significant? \| Summary \| Adjusted P Value \|  \| \|  \|  \|  \|  \|  \|  \|  \| \| C vs. L \| 5.537 \| 3.107 to 7.967 \| Yes \| **** \| <0.0001 \| E-F \| \| C vs. Nil+L \| -1.799 \| -4.256 to 0.6581 \| No \| ns \| 0.1934 \| E-G \| \| L vs. Nil+L \| -7.336 \| -9.881 to -4.791 \| Yes \| **** \| <0.0001 \| F-G \| | | | |
| **Figure 11d Golgi Hippocampus AO** | | | |
| \| Number of families \| 1 \|  \|  \|  \|  \|  \| \| --- \| --- \| --- \| --- \| --- \| --- \| --- \| \| Number of comparisons per family \| 3 \|  \|  \|  \|  \|  \| \| Alpha \| 0.05 \|  \|  \|  \|  \|  \| \|  \|  \|  \|  \|  \|  \|  \| \| Tukey's multiple comparisons test \| Mean Diff. \| 95.00% CI of diff. \| Significant? \| Summary \| Adjusted P Value \|  \| \|  \|  \|  \|  \|  \|  \|  \| \| C vs. L \| 3.754 \| -0.04486 to 7.552 \| No \| ns \| 0.0535 \| A-B \| \| C vs. Nil+L \| 1.214 \| -2.584 to 5.013 \| No \| ns \| 0.7256 \| A-C \| \| L vs. Nil+L \| -2.54 \| -6.338 to 1.259 \| No \| ns \| 0.2523 \| B-C \| | | | |
| **Figure 11d Golgi Hippocampus BS** | | | |
| \| Number of families \| 1 \|  \|  \|  \|  \|  \| \| --- \| --- \| --- \| --- \| --- \| --- \| --- \| \| Number of comparisons per family \| 3 \|  \|  \|  \|  \|  \| \| Alpha \| 0.05 \|  \|  \|  \|  \|  \| \|  \|  \|  \|  \|  \|  \|  \| \| Tukey's multiple comparisons test \| Mean Diff. \| 95.00% CI of diff. \| Significant? \| Summary \| Adjusted P Value \|  \| \|  \|  \|  \|  \|  \|  \|  \| \| C vs. L \| 4.394 \| 0.8221 to 7.965 \| Yes \| * \| 0.0121 \| E-F \| \| C vs. Nil+L \| 2.771 \| -0.8724 to 6.415 \| No \| ns \| 0.1699 \| E-G \| \| L vs. Nil+L \| -1.623 \| -5.153 to 1.907 \| No \| ns \| 0.5164 \| F-G \| | | | |
